# DDQ in mechanochemical C–N coupling reactions

**DOI:** 10.3762/bjoc.18.64

**Published:** 2022-06-01

**Authors:** Shyamal Kanti Bera, Rosalin Bhanja, Prasenjit Mal

**Affiliations:** 1 School of Chemical Sciences, National Institute of Science Education and Research (NISER), An OCC of Homi Bhabha National Institute, Bhubaneswar, PO Bhimpur-Padanpur, Via Jatni, District Khurda, Odisha 752050, India

**Keywords:** ball mill, 1*H*-benzo[*d*]imidazole, C(sp^2^)–H amidation, DDQ, mechanochemistry, quinazolin-4(3*H*)-one

## Abstract

2,3-Dichloro-5,6-dicyano-1,4-benzoquinone (DDQ) is a commonly known oxidant. Herein, we report that DDQ can be used to synthesize 1,2-disubstituted benzimidazoles and quinazolin-4(3*H*)-ones via the intra- and intermolecular C–N coupling reaction under solvent-free mechanochemical (ball milling) conditions. In the presence of DDQ, the intramolecular C(sp^2^)–H amidation of *N*-(2-(arylideneamino)phenyl)-*p*-toluenesulfonamides leads to 1,2-disubstituted benzimidazoles and the one-pot coupling of 2-aminobenzamides with aryl/alkyl aldehydes resulted in substituted quinazolin-4(3*H*)-one derivatives in high yields.

## Introduction

The reawakening approaches to use solvent-free and environmentally benign conditions in organic synthesis have facilitated new opportunities [1–4]. The research area of mechanochemistry [5–6] mainly focuses on conducting synthetic transformations in solid-state or solvent-free conditions. Mechanochemistry is one of the emerging avenues in chemistry that can make the world more sustainable by following the “Twelve Principles of Green Chemistry” [2]. Mechanochemistry is one of the ten innovative technologies that IUPAC recognized [7]. To perform organic transformations in a greener way, the mechanochemical methods can also be considered as one of the alternative approaches [8–10]. The one-pot multicomponent synthesis of important heterocycles can be the state of art practice by applying the strategies like domino, cascade, or tandem [11–13]. These environmentally friendly approaches set forth the journey of facilitating sustainable systems by using mechanochemical methods to access small organic compounds [3].

Due to the high reduction potential of the quinone moiety in 2,3-dichloro-5,6-dicyano-1,4-benzoquinone (DDQ), it was well established as a hydride transfer reagent in various organic reactions [14–15]. Generally, DDQ assists in dehydrogenation reactions in organic synthesis [16]. In this context, various carbon–heteroatom bond formation reactions such as C–P [17], C–O [18–20], and C–S [21] were achieved using DDQ as an oxidant [22–23]. In addition, the utilization of DDQ as a photoredox catalyst [24] and co-catalyst [25–26] have also been documented in organic synthesis [27]. DDQ-mediated oxidative C–N cross-coupling reactions are well known, but limited reports are available for reactions carried out under solvent-free conditions [28–29].

**Figure 1:**
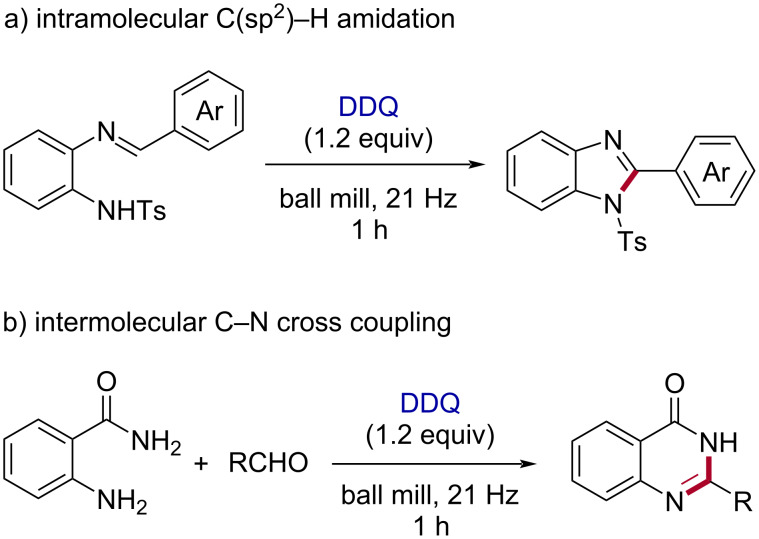
Our work on mechanochemical C–N coupling reactions using DDQ. The newly formed C–N bonds are shown as red lines for clarity. a) The intramolecular C(sp^2^)-H amidation leading to 1,2-disubstituted benzimidazoles. b) One-pot coupling to synthesize substituted quinazolin-4(3*H*)-ones.

However, improving environmentally benign methods [30–31] of C–N bond synthesis is of enormous significance [32–34]. In comparison to the metal-mediated C–N coupling reactions [35], the direct C–H amination is vital to provide many amine derivatives by sustainable methods [36–37]. The dehydrogenative C–N cross-coupling reactions from unreactive N–H and C–H bonds can lead to various nitrogen-containing heterocycles [32,38]. Herein, we disclose the DDQ-mediated oxidative C–N coupling toward the synthesis of 1,2-disubstituted benzimidazoles [39] under mechanochemical (ball milling) conditions (Figure 1a). In addition, the one-pot coupling of 2-aminobenzamides with aryl/alkyl aldehydes in the presence of DDQ resulted in substituted quinazolin-4(3*H*)-one [40] derivatives (Figure 1b).

## Results and Discussion

Towards the optimization study, (*E*)-*N*-(2-((2-bromobenzylidene)amino)phenyl)-4-methylbenzenesulfonamide (**1a**) was considered as a model substrate for the synthesis of 2-(2-bromophenyl)-1-tosyl-1*H*-benzo[*d*]imidazole (**2a**, Table 1). Initially, with 1.0 equiv of DDQ, product **2a** was obtained in 88% yield (Table 1, entry 1). However, in the presence of 1.2 equiv, the yield of **2a** increased to 97% (Table 1, entry 2). Further, with the increase of the amount of DDQ to 1.5 equiv, no further improvement was obtained (Table 1, entry 3). In addition, we have screened several iodine oxidation reagents, but none of them gave better yields (Table 1, entries 4–7). On the other hand, oxone as an oxidant yielded product **2a** with up to 43% yield (Table 1, entry 8). Similarly, we have optimized the reaction conditions for the synthesis of 2-phenylquinazolin-4(3*H*)-one (**5a**) from anthranilamide and benzaldehyde under the solvent-free conditions (Table S1, Supporting Information File 1). Notably, the use of 1.0 equiv of DDQ as oxidant, afforded the product 2-phenylquinazolin-4(3*H*)-one (**5a**) in 98% yield within 1 h. However, when reducing the amount of DDQ to 0.5 equiv the of product **5a** decreased to 48%. On the other hand, increasing the amount of DDQ to 1.2 equiv resulted in 98% yield of the product (Supporting Information File 1, Table S1, entries 1 and 3). Further studies revealed that other commonly used oxidants such as PIDA and oxone gave 30% and 61% the desired product, respectively (Supporting Information File 1, Table S1, entries 4 and 5). When molecular iodine or NIS were used as oxidants, product **5a** was obtained in 83% and 80% yield, respectively (Supporting Information File 1, Table S1, entries 6 and 7). However, the yield of the desired product **5a** slightly decreased to 92% with lowering of the operating milling frequency from 21 to 16 Hz (Supporting Information File 1, Table S1, entry 9). On the other hand, the yield of product **5a** was unaffected by increasing the operating frequency from 21 Hz to 25 Hz (Supporting Information File 1, Table S1, entry 8).

**Table 1:** Optimization of the reaction conditions.^a^

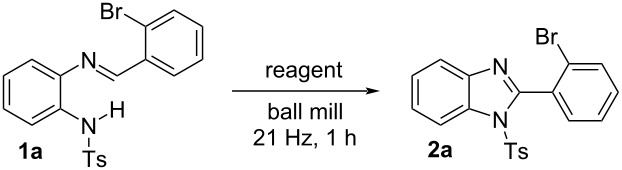		

Entry	Reagent (equiv)	Yield (%)^b^

1	DDQ (1.0)	88
2	DDQ (1.2)	97
3	DDQ (1.5)	96
4	NIS (1.2)	65
5	I_2_ (1.2)	32
6	I_2_ (1.2)/K_2_CO_3_ (1.2)	23
7	PIDA (1.2)	60
8	oxone (1.2)	43

^a^Reaction conditions: 0.14 mmol of **1a** and 0.167 mmol of DDQ (1.2 equiv) under solvent-free conditions were milled at 21 Hz in a 10 mL milling jar containing one stainless-steel grinding ball (15 mm in diameter) for 1 h. ^b^Yield of the isolated product after purification through silica-gel column chromatography.

**Figure 2:**
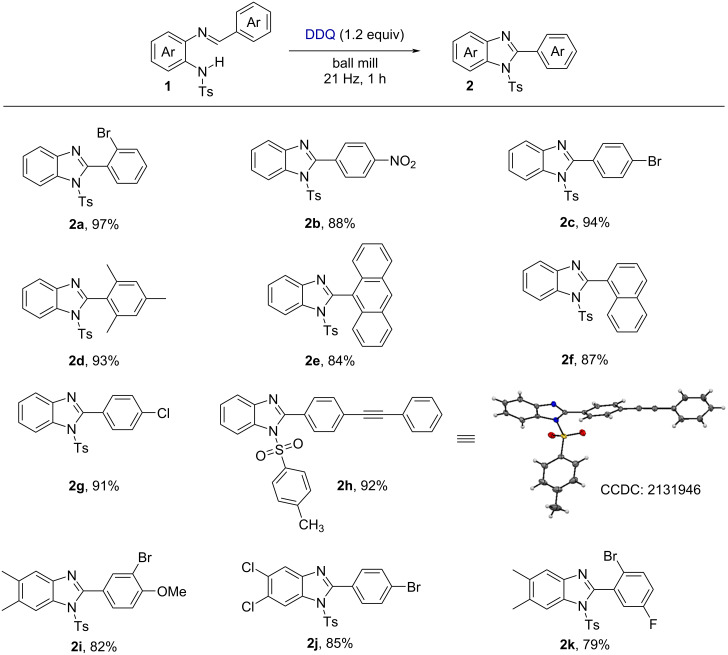
Scope of the mechanochemical synthesis of substituted benzimidazoles.

1,2-Disubstituted benzimidazoles are heterocyclic scaffolds holding a broad range of biological activities [41–43]. For example, telmisartan, a 1,2-disubstituted benzimidazole derivative, is extensively used as an antihypertensive agent [44]. The substrate scope for the synthesis of variously substituted benzimidazoles is shown in Figure 2. Benzimidazoles with a variety of aryl substituents (such as bromo, nitro, chloro, and 2,4,6-trimethyl) in the 2-position of the benzimidazole (**2a**–**d**,**g**) were obtained in good yields. Furthermore, the synthesis of the corresponding benzimidazoles with fused aromatic systems in the 2-position such as anthracenyl (**2e**) and naphthyl (**2f**) proved to be efficient. Similarly, the 5,6-dimethyl- or 5,6-dichloro-1,2-disubstituted benzimidazoles **2i**, **2j**, and **2k** were synthesized with 82, 85, and 79% yield, respectively. In addition, the structure of the synthesized 2-(4-(phenylethynyl)phenyl)-substituted product **2h** was established from the X-ray crystallography data.

**Figure 3:**
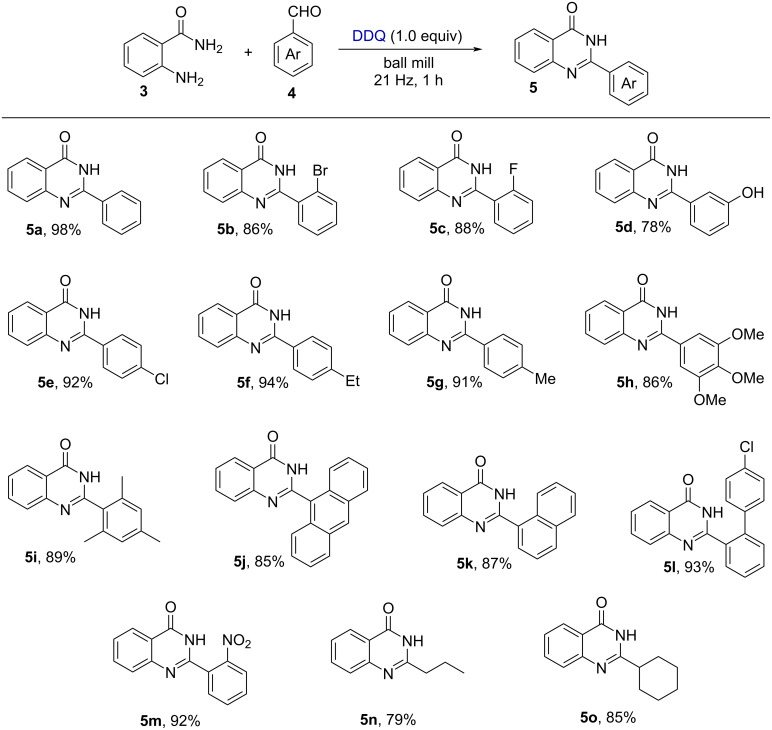
Synthesis of quinazolin-4(3*H*)-one derivatives.

Various methodologies are available in the literature toward constructing quinazolin-4(3*H*)-one derivatives [40,45–46]. Quinazolin-4(3*H*)-ones and its derivatives possess several biological activities such as antibacterial [47], antiviral [48], antitumor [49–50], antimalarial [51], anti-inflammatory [52], etc. We therefore investigated the scope of the one-pot coupling of 2-aminobenzamides with aryl/alkyl aldehydes in the presence of DDQ under the optimized mechanochemical conditions and the results are shown in Figure 3.

Unsubstituted anthranilamides reacted smoothly with various substituted aldehydes (containing a bromo, fluoro, hydroxy, chloro, ethyl, or methyl group) affording the corresponding products **5b**–**g** with good to excellent yields. Also, 3,4,5-trimethoxy-, 2,4,6-trimethyl-, anthracene-9-yl-, and naphthalene-1-yl-substituted benzaldehydes were well tolerated and gave the desired products **5h**–**k** with high yields. In this context, biphenyl aldehyde with a chloro group was efficiently converted to **5l** with 93% yield. Furthermore, aromatic aldehydes having a strong electron-withdrawing group (such as NO_2_) were smoothly converted to the corresponding products **5m** with 92% yield, respectively. In addition to this, aliphatic aldehydes (cyclohexanecarbaldehyde, butyraldehyde) were well tolerated under the standard reaction conditions to produce the product **5n** and **5o** with good yield.

**Figure 4:**
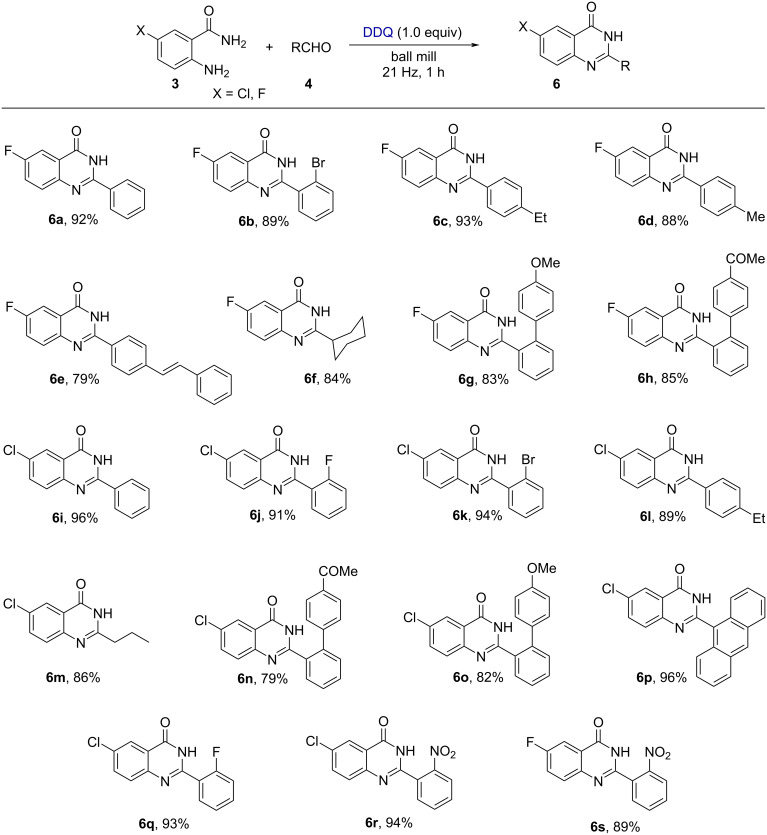
The substrate scope for the synthesis of quinazolin-4(3*H*)-one derivatives.

The substrate scope of this methodology was extended to chloro and fluoro-substituted anthranilamides and aldehydes (Figure 4). Initially, 2-amino-5-fluorobenzamide was reacted with various benzaldehydes having bromo, ethyl, methyl, styryl, and cyclohexyl groups to produce the respective cyclized products **6b–f** with good to excellent yields. Similarly, biphenyl aldehydes with -OMe and -COMe groups were well tolerated under the standard reaction conditions and delivered the corresponding products **6g** and **6h** with 83 and 85% yields, respectively. In addition, we have also explored the substrate scope with 2-amino-5-chlorobenzamide and various aldehydes. Benzaldehyde containing fluoro, bromo, ethyl, and anthryl groups led to the corresponding products **6j**, **6k**, **6l**, and **6p** in good to excellent yield. Aliphatic aldehydes such as butyraldehyde gave the cyclized product **6m** with an 86% yield. In this regard, an -OMe and -COMe group-containing biphenyl aldehyde resulted in the corresponding products **6n** and **6o** with 79 and 82% yields, respectively. Nitro and fluoro-substituted aromatic aldehydes efficiently reacted with the chloro and fluoro-substituted anthranilamides and delivered the corresponding products **6q** and **6r** with good yields.

**Figure 5:**
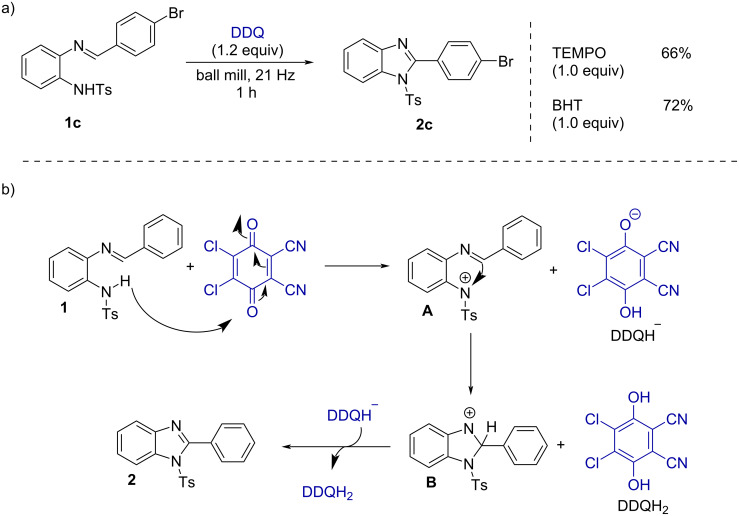
a) Control experiment and b) Plausible mechanism.

To understand the reaction mechanism, we have performed radical trapping experiments using TEMPO and BHT in the reaction of substrate **1c** (Figure 5a). Under the standard reaction conditions in the presence of TEMPO or BHT, the expected product **2c** was formed in 66 and 72% yields. These results indicate that a radical pathway may not be involved in the reaction. So, based on literature reports [53–55], we have proposed a reaction mechanism in Figure 5b. Initially, DDQ abstracts a hydride ion from substrate **1a** to generate the intermediate **A**. Then intermediate **A** undergoes an electrophilic intramolecular cyclization to form the cationic intermediate **B**, followed by hydride abstraction to generate the desired product **2a**. On the other hand, the formation of quinazolin-4(3*H*)-ones starts with the formation of an imine intermediate and then it will follow the similar mechanistic pathway.

**Figure 6:**
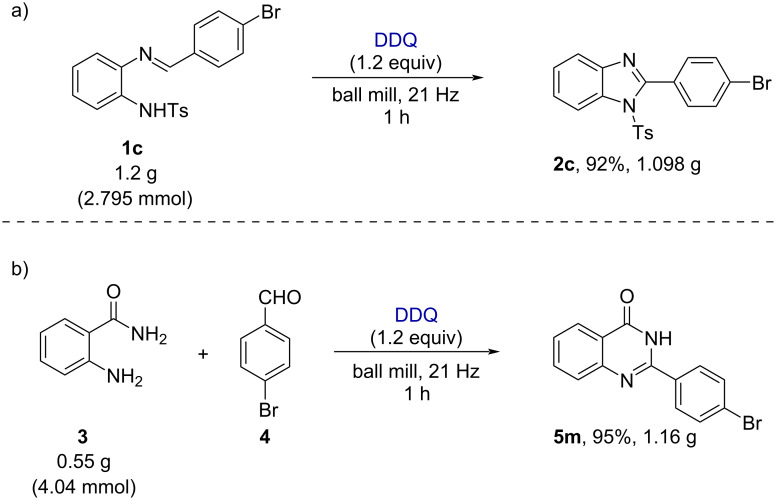
Large-scale synthesis. a) 1,2-Disubstituted benzimidazoles. b) Substituted quinazolin-4(3*H*)-ones. Reaction conditions: reactants were milled at 21 Hz in a 25 mL milling jar containing one stainless-steel grinding ball (15 mm in diameter).

To explore the synthetic utility of the oxidative C–N cross-coupling reaction, we have performed the large-scale synthesis under the solvent-free (ball milling) conditions as shown in Figure 6. In this context, milling of the substrate (*E*)-*N*-(2-((4-bromobenzylidene)amino)phenyl)-4-methylbenzenesulfonamide (**1c**, 2.795 mmol) in the presence of 1.2 equiv of DDQ delivered 1.098 g (92%) of the cyclized product 2-(4-bromophenyl)-1-tosyl-1*H*-benzo[*d*]imidazole (**2c**). Similarly, we also carried out the large-scale synthesis with 4.04 mmol each of anthranilamide and 4-bromobenzaldehyde (**4**), which produced 1.16 g (95%) of the desired product 2-(4-bromophenyl)quinazolin-4(3*H*)-one (**5m**).

## Conclusion

In summary, we have successfully developed efficient methodologies for synthesizing 1,2-disubstituted benzimidazoles and quinazolin-4(3*H*)-one derivatives under mechanochemical (ball milling at 21 Hz) conditions in the presence of DDQ. The developed methodology can be considered as a green and eco-friendly methodology due to its solvent-free and metal-free nature. So, it can also be regarded as an alternative pathway to the traditional solution-based protocols. We anticipate that our developed strategy will have a substantial impact on the field of organic synthesis.

## Supporting Information

File 1 Experimental details, characterization data, copies of NMR spectra and X-ray crystallography details.

## References

[1] Hernández J G, Bolm C (2017). J Org Chem.

[2] Ardila‐Fierro K J, Hernández J G (2021). ChemSusChem.

[3] Egorov I N, Santra S, Kopchuk D S, Kovalev I S, Zyryanov G V, Majee A, Ranu B C, Rusinov V L, Chupakhin O N (2020). Green Chem.

[4] Tan D, Friščić T (2018). Eur J Org Chem.

[5] Wang G-W (2013). Chem Soc Rev.

[6] Mateti S, Mathesh M, Liu Z, Tao T, Ramireddy T, Glushenkov A M, Yang W, Chen Y I (2021). Chem Commun.

[7] Gomollón-Bel F (2019). Chem Int.

[8] Bose A, Mal P (2019). Beilstein J Org Chem.

[9] Achar T K, Bose A, Mal P (2017). Beilstein J Org Chem.

[10] O’Neill R T, Boulatov R (2021). Nat Rev Chem.

[11] Lou S-J, Mao Y-J, Xu D-Q, He J-Q, Chen Q, Xu Z-Y (2016). ACS Catal.

[12] Das D, Bhosle A A, Panjikar P C, Chatterjee A, Banerjee M (2020). ACS Sustainable Chem Eng.

[13] Bera S K, Mal P (2021). J Org Chem.

[14] Alsharif M A, Raja Q A, Majeed N A, Jassas R S, Alsimaree A A, Sadiq A, Naeem N, Mughal E U, Alsantali R I, Moussa Z (2021). RSC Adv.

[15] Wendlandt A E, Stahl S S (2015). Angew Chem, Int Ed.

[16] Cheng D, Wu L, Deng Z, Xu X, Yan J (2017). Adv Synth Catal.

[17] Chen Q, Wen C, Wang X, Yu G, Ou Y, Huo Y, Zhang K (2018). Adv Synth Catal.

[18] Li J-S, Xue Y, Fu D-M, Li D-L, Li Z-W, Liu W-D, Pang H-L, Zhang Y-F, Cao Z, Zhang L (2014). RSC Adv.

[19] Yi H, Liu Q, Liu J, Zeng Z, Yang Y, Lei A (2012). ChemSusChem.

[20] Pan D, Pan Z, Hu Z, Li M, Hu X, Jin L, Sun N, Hu B, Shen Z (2019). Eur J Org Chem.

[21] Li C, Li J, Tan C, Wu W, Jiang H (2018). Org Chem Front.

[22] Yan B, Fu Y, Zhu H, Chen Z (2019). J Org Chem.

[23] Zhai L, Shukla R, Rathore R (2009). Org Lett.

[24] Natarajan P, König B (2021). Eur J Org Chem.

[25] Song C, Dong X, Yi H, Chiang C-W, Lei A (2018). ACS Catal.

[26] Shen Z, Dai J, Xiong J, He X, Mo W, Hu B, Sun N, Hu X (2011). Adv Synth Catal.

[27] Mandal T, Azim A, Das S, De Sarkar S (2022). Asian J Org Chem.

[28] Sun C, Zheng L, Xu W, Dushkin A V, Su W (2020). Green Chem.

[29] Hernández J G, Turberg M, Schiffers I, Bolm C (2016). Chem – Eur J.

[30] Li C-J (2016). Chem.

[31] Hermann G N, Bolm C (2017). ACS Catal.

[32] Bariwal J, Van der Eycken E (2013). Chem Soc Rev.

[33] Hartwig J F (2008). Acc Chem Res.

[34] Majumdar B, Sarma D, Bhattacharya T, Sarma T K (2017). ACS Sustainable Chem Eng.

[35] Ruiz-Castillo P, Buchwald S L (2016). Chem Rev.

[36] Sun C-L, Shi Z-J (2014). Chem Rev.

[37] Louillat M-L, Patureau F W (2014). Chem Soc Rev.

[38] Carvalho L C R, Fernandes E, Marques M M B (2011). Chem – Eur J.

[39] Maiti S, Mal P (2015). Adv Synth Catal.

[40] Alam M T, Maiti S, Mal P (2018). Beilstein J Org Chem.

[41] Li Y-F, Wang G-F, He P-L, Huang W-G, Zhu F-H, Gao H-Y, Tang W, Luo Y, Feng C-L, Shi L-P (2006). J Med Chem.

[42] Valdez J, Cedillo R, Hernández-Campos A, Yépez L, Hernández-Luis F, Navarrete-Vázquez G, Tapia A, Cortés R, Hernández M, Castillo R (2002). Bioorg Med Chem Lett.

[43] Sondhi S M, Rajvanshi S, Johar M, Bharti N, Azam A, Singh A K (2002). Eur J Med Chem.

[44] Sharpe M, Jarvis B, Goa K L (2001). Drugs.

[45] Parua S, Das S, Sikari R, Sinha S, Paul N D (2017). J Org Chem.

[46] Sahoo S, Pal S (2021). J Org Chem.

[47] Kung P-P, Casper M D, Cook K L, Wilson-Lingardo L, Risen L M, Vickers T A, Ranken R, Blyn L B, Wyatt J R, Cook P D (1999). J Med Chem.

[48] Chen M, Li P, Hu D, Zeng S, Li T, Jin L, Xue W, Song B (2016). Bioorg Med Chem Lett.

[49] Chandrika P M, Yakaiah T, Rao A R R, Narsaiah B, Reddy N C, Sridhar V, Rao J V (2008). Eur J Med Chem.

[50] Cao S-L, Feng Y-P, Jiang Y-Y, Liu S-Y, Ding G-Y, Li R-T (2005). Bioorg Med Chem Lett.

[51] Kikuchi H, Yamamoto K, Horoiwa S, Hirai S, Kasahara R, Hariguchi N, Matsumoto M, Oshima Y (2006). J Med Chem.

[52] Ozaki K-i, Yamada Y, Oine T, Ishizuka T, Iwasawa Y (1985). J Med Chem.

[53] Batra A, Singh K N (2020). Eur J Org Chem.

[54] Cheng D, Bao W (2008). J Org Chem.

[55] Guo X, Zipse H, Mayr H (2014). J Am Chem Soc.

