# Supplementary material for: DDQ in mechanochemical C–N coupling reactions
Source: Beilstein J Org Chem. 2022 Jun 1;18:639–46. doi: 10.3762/bjoc.18.64 (PMC9174842; doi:10.3762/bjoc.18.64)
Supplement: File 1 — Experimental details, characterization data, copies of NMR spectra and X-ray crystallography details. [file Beilstein_J_Org_Chem-18-639-s001.pdf]

BEILSTEIN JOURNAL OF ORGANIC CHEMISTRY

Supporting Information

for

DDQ in mechanochemical C–N coupling reactions

Shyamal Kanti Bera, Rosalin Bhanja and Prasenjit Mal

Beilstein J. Org. Chem. **2022,** *18,* 639–646. doi:10.3762/bjoc.18.64

Experimental details, characterization data, copies of NMR spectra and X-ray crystallography details

License and Terms: This is a supporting information file under the terms of the Creative Commons Attribution License (https://creativecommons.org/licenses/by/4.0). Please note that the reuse, redistribution and reproduction in particular requires that the author(s) and source are credited and that individual graphics may be subject to special legal provisions.
The license is subject to the *Beilstein Journal of Organic Chemistry* terms and conditions: (https://www.beilstein-journals.org/bjoc/terms)

Table of contents

General methods and experimental procedures S2–S4

Optimization of the reaction conditions S5

X-ray crystallography analysis S6–S7

Characterization data of synthesized compounds S8–S24

References S24–S25

Copies of NMR spectra S26–S71

General methods and experimental procedures

General Information. Commercially available reagents and solvents were used as received. Column chromatographic purifications of the compounds were performed using silica gel (mesh 230–400, 100–200) and hexane/ethyl acetate solvent mixtures. NMR spectra were recorded on a 400 MHz or 700 MHz instrument at 25 °C. The chemical shift values are reported in parts per million (ppm) with respect to residual trichloromethane (7.26 ppm for ^{1}H NMR and 77.16 ppm for ^{13}C NMR) or dimethyl sulfoxide (2.50 ppm for ^{1}H NMR and 39.52 ppm for ^{13}C NMR). The peak patterns are designated as follows: s: singlet; d: doublet; t: triplet; q: quartet; m: multiplet; dd: doublet of doublets; td: triplet of doublets; brs: broad singlet. The coupling constants (*J*) are reported in hertz (Hz). High-resolution mass spectra (HRMS) were recorded on an ESI-TOF (time of flight) mass spectrometer. Infrared spectral data are reported in wave numbers (cm^{-1}). All reactions were carried out in a Mixer Mill MM 200 having a maximum operating frequency of 25 Hz. A 10 mL milling jar with one grinding ball (15 mm diameter, stainless steel) was used for the optimization of the reaction and substrate scopes. On the other hand, a 25 mL milling jar with one grinding ball (15 mm diameter, stainless steel) was used for the large-scale synthesis. FTIR spectra were recorded using thin layers of the compounds on the surface of NaCl crystal using dichloromethane. Melting points of the compounds were determined using a digital melting point apparatus and are uncorrected.

General procedure for the preparation of (*E*)-*N*-(2-(benzylideneamino)phenyl)-4-methylbenzenesulfonamide derivatives.

Synthesis of *N*-(2-aminophenyl)-4-methylbenzenesulfonamide [1]: *p*-Toluenesulfonyl chloride (1.849 g, 9.702 mmol) in THF (10 mL) was added dropwise to a stirred solution of *o*-phenylenediamine (1 g, 9.24 mmol) and pyridine (1.12 mL**,** 13.86 mmol) in THF (12 mL) at 0 °C. Then, the reaction mixture was allowed to stir at room temperature for 10 to12 h and the

progress of the reaction was monitored by thin layer chromatography (TLC). After completion of the reaction, the solvent was evaporated in vacuo. A saturated solution of ammonium chloride was added to residue and ethyl acetate was used to separate the organic layer. Then, the organic layer was washed, dried over Na_2SO_4, evaporated, and purified by silica gel column chromatography with hexane/ethyl acetate mixture as eluent to get the pure product.

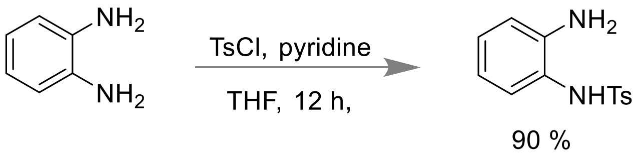

Scheme S1: Preparation of *N*-(2-aminophenyl)-4-methylbenzenesulfonamide.

Preparation of (*E*)-*N*-(2-((2-bromobenzylidene)amino)phenyl)-4-methylbenzene-sulfonamide [1]: To an oven-dried 100 mL seal-tube, 2-bromobenzaldehyde (3.812 mmol, 1 equiv) was added to a stirred solution of *N*-(2-aminophenyl)-4-methylbenzenesulfonamide (1 g, 3.812 mmol, 1 equiv) in 15 mL ethanol as solvent. The reaction mixture was refluxed at 80 °C for typically 2–8 h and the progress of the reaction was monitored by TLC. Then, the reaction mixture was cooled at room temperature followed by filtration and recrystallization using ethanol as solvent to obtain the desired product.

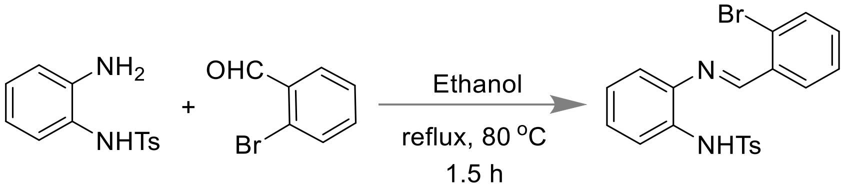

Scheme S2: Synthesis of (*E*)-*N*-(2-((2-bromobenzylidene)amino)phenyl)-4-methylbenzene-sulfonamide.

Synthesis of 2-(2-bromophenyl)-1-tosyl-1*H*-benzo[*d*]imidazole: (*E*)-*N*-(2-((2-bromo-benzylidene)amino)phenyl)-4-methylbenzenesulfonamide (**1a**, 60 mg 0.14 mmol) and DDQ (38 mg, 0.167 mmol) were placed in a 10 mL of stainless-steel jar containing one stainless-steel milling ball. The milling process was carried out for 1 h and the progress of the reaction was monitored by TLC. After complete consumption of the starting material, the reaction mixture was extracted with 15 mL (3 × 5 mL) dichloromethane. Then, the extract was evaporated to dryness in vacuo and the residue purified by silica gel column chromatography using hexane/ethyl acetate mixture as eluent to afford the desired product.

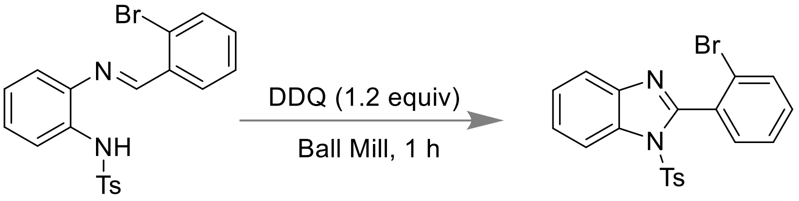

Scheme S3. Preparation of 2-(2-bromophenyl)-1-tosyl-1*H*-benzo[*d*]imidazole.

Synthesis of 2-phenylquinazolin-4(3*H*)-one: In a 10 mL of stainless-steel jar, 2-aminobenzamide (60 mg, 0.441 mmol), benzaldehyde (0.441 mmol, 1 equiv), DDQ (100 mg, 0.441 mmol) and one stainless-steel milling ball were added. Then, the milling process was carried out for 1 h at 21 Hz and the progress of the reaction was monitored by TLC. After consumption of all starting material, the reaction mixture was extracted with 15 mL (3 × 5 mL) dichloromethane. The organic extract was evaporated to dryness in vacuum and the residue purified by silica gel column chromatography with hexane/ethyl acetate mixture as eluent.

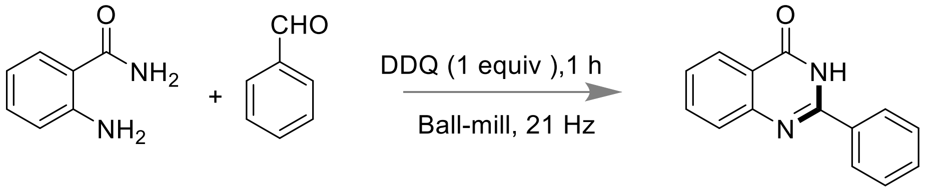

Scheme S4. Procedure for the synthesis of 2-phenylquinazolin-4(3*H*)-one under ball-milling.

Optimization of the reaction conditions

Table S1. Optimization of the reaction conditions[a].

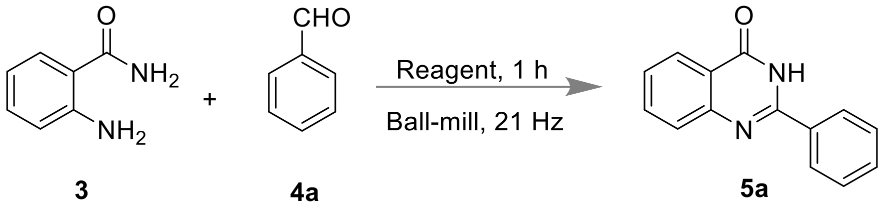

Entry	Reagent (equiv)	Yield (%) [b]
1	DDQ (0.5)	48%
2	DDQ (1)	98%
3	DDQ (1.2)	98%
4	PIDA (1)	30%
5	Oxone (1)	61%
6	I_2 (1)	83%
7	NIS (1)	80%
8[c]	DDQ (1)	98%
9[d]	DDQ (1)	92%

[a]Reaction conditions: 0.44 mmol of **1a**, 0.44 mmol of **2a** and 0.44 mmol of DDQ (1 equiv) under solvent free conditions for 1 h. [b]Yield of isolated product after purification through silica gel column chromatography. [c]Reaction was performed at 25 Hz for 1 h. [d]Reaction was performed at 16 Hz for 1 h.

X-ray crystallography analysis

Procedure for preparing the crystal sample. In a 10 mL round-bottomed flask 20 mg of 2-(4-(phenylethynyl)phenyl)-1-tosyl-1*H*-benzo[*d*]imidazole (**2h**) was dissolved in an ethyl acetate/hexane 1:1 mixture (6 mL). After that the solution was allowed for slow evaporation to obtain a good quality of crystal.

Crystal measurement

The crystals data were collected with a Bruker SMART D8 goniometer equipped with an APEX CCD detector and with an INCOATEC micro source (MoKα radiation, λ = 0.71073 Å). SAINT+ [2] and SADABS [3] were used to integrate the intensities and to correct the absorption, respectively The structure was resolved by direct methods and refined on F^2 with SHELXL-97 [4]. ORTEP drawing of the compounds show ellipsoid contour at the 50% probability level.

Crystallographic data

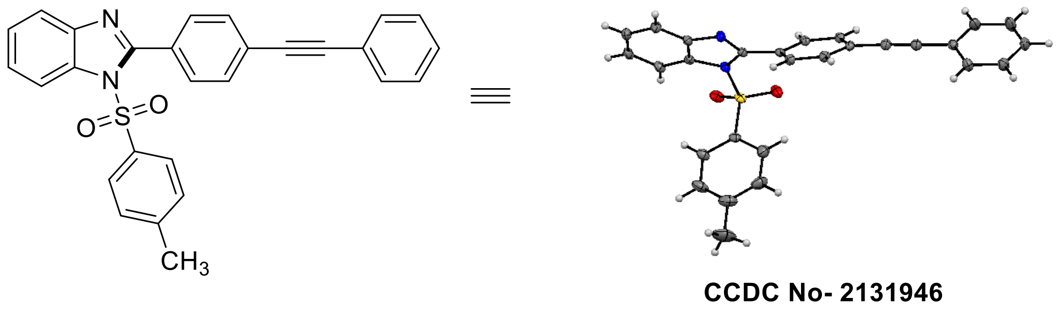

Figure S1. Crystal structure of **2h** (50% ellipsoid probability).

Table S1. Crystal data and structure refinement for **2h.**

CCDC No	2131946
Empirical formula	$C_{28}H_{20}N_2O_2S$
Formula weight	448.52
Temperature/K	100.00(10)
Crystal system	monoclinic

Space group	$P2_1/n$
a/Å	11.4045(2)
b/Å	17.0759(3)
c/Å	12.4829(3)
α/°	90
β/°	111.254(2)
γ/°	90
Volume/Å^3	2265.60(8)
Z	4
ρ_{calc}g/cm^3	1.315
μ/mm^{-1}	0.171
F(000)	936.0
Crystal size/mm^3	0.2 × 0.18 × 0.18
Radiation	Mo Kα (λ = 0.71073)
2Θ range for data collection/°	6.51 to 60.856
Index ranges	-15 ≤ h ≤ 15, -23 ≤ k ≤ 22, -17 ≤ l ≤ 15
Reflections collected	40751
Independent reflections	5879 [R_{int} = 0.0404, R_{sigma} = 0.0239]
Data/restraints/parameters	5879/0/299
Goodness-of-fit on F^2	1.051
Final R indexes [I>=2σ (I)]	R_1 = 0.0375, wR_2 = 0.0970
Final R indexes [all data]	R_1 = 0.0433, wR_2 = 0.1007
Largest diff. peak/hole / e Å^{-3}	0.56/-0.39

Characterization data of synthesized compounds

2-(2-Bromophenyl)-1-tosyl-1*H*-benzo[*d*]imidazole (2a) [5]: R_f = 0.5 (hexane:ethyl acetate 4:1); white solid; Yield 97% ; ^{1}H NMR (700 MHz, $CDCl_3$) δ 8.16 (d, *J* = 8.4 Hz, 1H), 7.79 (d, *J* = 7.7 Hz, 1H), 7.66 (d, *J* = 7.7 Hz, 1H), 7.54 (d, *J* = 8.4 Hz, 2H), 7.47 (t, *J* = 7.7 Hz, 1H), 7.45 – 7.39 (m, 4H), 7.20 (d, *J* = 8.4 Hz, 2H), 2.37 (s, 3H); ^{13}C NMR (175 MHz, $CDCl_3$) δ 150.8, 146.2, 142.2, 135.2, 132.9, 132.7, 132.6, 132.1, 131.8, 130.0, 127.6, 126.6, 125.9, 125.1, 124.8, 120.9, 114.3, 21.8.

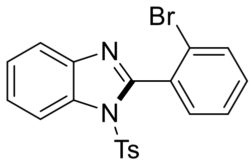

2-(4-Nitrophenyl)-1-tosyl-1*H*-benzo[*d*]imidazole (2b) [1]: R_f = 0.4 (hexane:ethyl acetate 4:1); yellow solid; Yield 88% ; ^{1}H NMR (400 MHz, $CDCl_3$) δ 8.33 (d, *J* = 8.4 Hz, 2H), 8.19 (d, *J* = 8.0 Hz, 1H), 7.85 (d, *J* = 8.4 Hz, 2H), 7.75 (d, *J* = 8.0 Hz, 1H), 7.49 (t, *J* = 7.6 Hz, 1H), 7.43 (t, *J* = 7.6 Hz, 1H), 7.36 (d, *J* = 8.0 Hz, 2H), 7.14 (d, *J* = 8.0 Hz, 2H), 2.34 (s, 3H); ^{13}C NMR (100 MHz, $CDCl_3$) δ 151.7, 149.1, 146.4, 142.8, 136.5, 134.8, 133.9, 132.1, 130.1, 126.9, 126.4, 125.9, 122.9, 120.9, 115.3, 21.8.

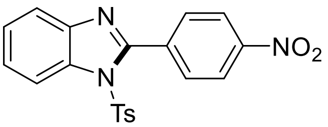

2-(4-Bromophenyl)-1-tosyl-1*H*-benzo[*d*]imidazole (2c) [1]: R_f = 0.5 (hexane:ethyl acetate 4:1); white solid; Yield 94% ; ^{1}H NMR (400 MHz, $CDCl_3$) δ 8.19 (d, *J* = 8.0 Hz, 1H), 7.72 (d, *J* = 7.6 Hz, 1H), 7.62 (s, 1H), 7.60 (s, 1H), 7.52 (s, 1H), 7.50 (s, 1H), 7.45 (t, *J* = 7.6 Hz, 1H), 7.40 (t, *J* = 7.6 Hz, 1H), 7.35 (s, 1H), 7.33 (s, 1H), 7.13 (s, 1H), 7.11 (s, 1H), 2.33 (s, 3H); ^{13}C NMR (100 MHz, $CDCl_3$) δ 153.2, 146.1, 142.8, 135.0, 134.0, 132.5, 131.1, 129.9, 129.1, 127.0, 125.8, 125.6, 125.5, 120.6, 115.4, 21.8.

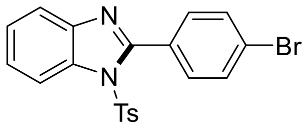

2-Mesityl-1-tosyl-1*H*-benzo[*d*]imidazole (2d) [5]: R_f = 0.6 (hexane:ethyl acetate 4:1); white solid; Yield 93% ; ^{1}H NMR (400 MHz, $CDCl_3$) δ 8.27 (d, *J* = 8.0 Hz, 1H), 7.78 (d, *J* = 8.0 Hz, 1H), 7.46 (t, *J* = 7.2 Hz, 3H), 7.41 (t, *J* = 7.6 Hz, 1H), 7.18 (d, *J* = 8.0 Hz, 2H), 6.88 (s, 2H), 2.38 (s, 3H), 2.37 (s, 3H), 1.77 (s, 6H); ^{13}C NMR (100 MHz, $CDCl_3$) δ 151.9, 145.9, 142.4, 139.9, 138.8, 135.4, 133.2, 129.8, 127.9, 127.8, 126.9, 125.3, 124.7, 120.6, 114.3, 21.8, 21.5, 20.0 (×2).

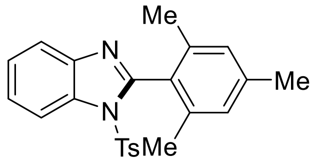

2-(Anthracen-9-yl)-1-tosyl-1*H*-benzo[*d*]imidazole (2e) [1]: R_f = 0.45 (hexane:ethyl acetate 4:1); yellow solid; Yield 84% ; ^{1}H NMR (400 MHz, $CDCl_3$) δ 8.50 (s, 1H), 8.38 (d, *J* = 8.0 Hz, 1H), 7.96 (s, 1H), 7.94 (s, 1H), 7.61 (t, *J* = 7.6 Hz, 1H), 7.56 (t, *J* = 7.6 Hz, 1H), 7.47 – 7.35 (m, 3H), 7.22 – 7.14 (m, 2H), 7.08 (s, 1H), 7.06 (s, 1H), 6.95 (s, 1H), 6.93 (s, 1H), 6.77 (s, 1H), 6.75 (s, 1H), 2.22 (s, 3H); ^{13}C NMR (100 MHz, $CDCl_3$) δ 150.6, 149.4, 145.9, 134.0, 133.3, 131.7, 130.7, 130.4, 129.6, 128.5, 128.3, 127.6, 127.0, 126.2, 125.5, 125.3, 120.4, 114.6, 112.2, 21.7.

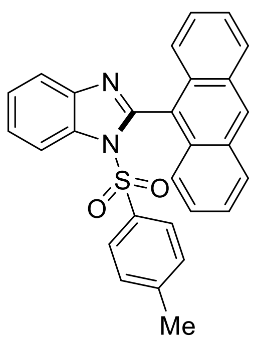

2-(Naphthalen-1-yl)-1-tosyl-1*H*-benzo[*d*]imidazole (2f) [5]: R_f = 0.5 (hexane:ethyl acetate 4:1); white solid; Yield 87% ; ^{1}H NMR (400 MHz, $CDCl_3$) δ 8.31 (d, *J* = 8.0 Hz, 1H), 7.97 (d, *J* = 8.0 Hz, 1H), 7.85 (d, *J* = 8.0 Hz, 1H), 7.81 (d, *J* = 8.0 Hz, 1H), 7.55 (dt, *J* = 11.2, 7.2 Hz, 3H), 7.48 (t, *J* = 7.6 Hz, 1H), 7.40 (t, *J* = 7.2 Hz, 1H), 7.21 – 7.11 (m, 4H), 6.88 (s, 1H), 6.86 (s, 1H), 2.22 (s, 3H); ^{13}C NMR (100 MHz, $CDCl_3$) δ 151.58, 145.92, 141.71, 134.72, 133.47, 132.96, 132.36, 131.01, 130.19, 129.64, 128.19, 127.4, 126.9, 126.8, 126.1, 125.9, 125.3, 125.1, 124.5, 120.4, 114.8, 21.6.

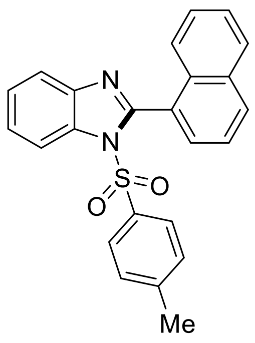

2-(4-Chlorophenyl)-1-tosyl-1*H*-benzo[*d*]imidazole (2g) [1]: R_f = 0.5 (hexane:ethyl acetate 4:1); white solid; Yield 91% ; ^{1}H NMR (400 MHz, $CDCl_3$) δ 8.20 (d, *J* = 8.0 Hz, 1H), 7.72 (d, *J* = 7.6 Hz, 1H), 7.59 (s, 1H), 7.57 (s, 1H), 7.46 (s, 1H), 7.44 (s, 1H), 7.44 -7.36 (m, 2H), 7.34 (s, 1H), 7.32 (s, 1H), 7.12 (s, 1H), 7.10 (s, 1H), 2.33 (s, 3H); ^{13}C NMR (100 MHz, $CDCl_3$) δ 153.1, 146.0, 142.8, 137.1, 135.0, 134.0, 132.3, 129.9, 128.7, 128.2, 127.0, 125.8, 125.6, 120.6, 115.3, 21.8.

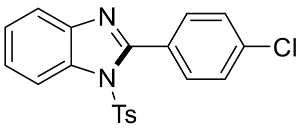

2-(4-(Phenylethynyl)phenyl)-1-tosyl-1*H*-benzo[*d*]imidazole (2h) [1]: R_f = 0.4 (hexane:ethyl acetate 4:1); white solid; Yield 92% ; ^{1}H NMR (400 MHz, $CDCl_3$) δ 8.22 (d, *J* = 8.0 Hz, 1H), 7.73 (d, *J* = 7.6 Hz, 1H), 7.63 (s, 4H), 7.61 – 7.56 (m, 2H), 7.45 (s, 1H), 7.44 – 7.36 (m, 4H), 7.34 (d, *J* = 8.0 Hz, 2H), 7.11 (d, *J* = 8.0 Hz, 2H), 2.33 (s, 3H); ^{13}C NMR (100 MHz, $CDCl_3$) δ 153.7, 145.9, 142.9, 135.1, 134.1, 131.9, 131.0, 130.9, 129.9, 129.8, 128.8, 128.6, 127.1, 125.8, 125.6, 123.0, 120.6, 115.4, 91.7, 89.0, 21.8.

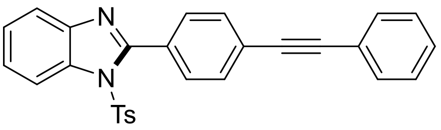

2-(3-Bromo-4-methoxyphenyl)-5,6-dimethyl-1-tosyl-1*H*-benzo[*d*]imidazole (2i) [1]: R_f = 0.4 (hexane:ethyl acetate 4:1); white solid; Yield 82% ; ^{1}H NMR (400 MHz, $CDCl_3$) δ 7.95 (s, 1H), 7.63 (d, *J* = 6.4 Hz, 2H), 7.44 (s, 1H), 7.31 (d, *J* = 8.0 Hz, 2H), 7.11 (d, *J* = 8.0 Hz, 2H), 6.98 (d, *J* = 9.2 Hz, 1H), 3.99 (s, 3H), 2.44 (s, 3H), 2.35 (s, 3H), 2.33 (s, 3H). ^{13}C NMR (100 MHz, $CDCl_3$) δ 157.6, 151.9, 145.8, 141.2, 135.3, 135.2, 134.9, 134.6, 132.5, 131.9, 129.8, 126.9, 123.8, 120.5, 115.5, 110.9, 110.7, 56.5, 21.7, 20.9, 20.3.

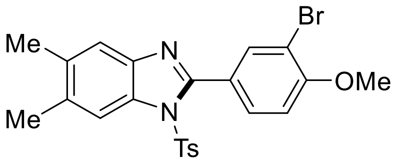

2-(4-Bromophenyl)-5,6-dichloro-1-tosyl-1*H*-benzo[*d*]imidazole (2j) [1]: R_f = 0.6 (hexane:ethyl acetate 4:1); white solid; Yield 85% ; ^{1}H NMR (400 MHz, $CDCl_3$) δ 8.33 (s,

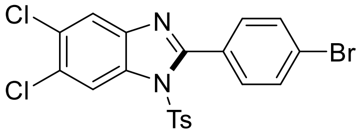

1H), 7.79 (s, 1H), 7.63 (s, 1H), 7.61 (s, 1H), 7.48 (s, 1H), 7.46 (s, 1H), 7.30 (s, 1H), 7.28 (s, 1H), 7.16 (s, 1H), 7.14 (s, 1H), 2.36 (s, 3H); ^{13}C NMR (100 MHz, $CDCl_3$) δ 154.8, 146.7, 141.9, 134.5, 133.1, 132.5, 131.3, 130.2, 130.0, 129.9, 128.2, 127.1, 126.1, 121.7, 116.8, 21.8.

2-(2-Bromo-5-fluorophenyl)-5,6-dimethyl-1-tosyl-1*H*-benzo[*d*]imidazole (2k) [1]: R_f = 0.5 (hexane:ethyl acetate 4:1); white solid; Yield 79% ; ^{1}H NMR (400 MHz, $CDCl_3$) δ 7.92 (s, 1H), 7.63 – 7.59 (m, 1H), 7.56 (s, 1H), 7.54 (d, *J* = 4.4 Hz, 2H), 7.21 (d, *J* = 8.0 Hz, 2H), 7.16

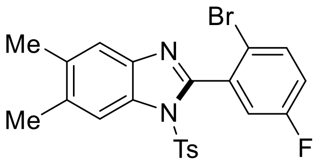

– 7.04 (m, 2H), 2.45 (s, 3H), 2.37 (s, 6H); ^{13}C NMR (100 MHz, $CDCl_3$) δ 160.9 (d, *J* = 248.6 Hz), 148.6 (d, *J* = 2.0 Hz), 146.1, 140.7, 135.5, 135.2, 134.4, 133.9 (d, *J* = 7.9 Hz), 133.8, 131.2, 130.1, 127.4, 120.9, 119.8 (d, *J* = 24.1 Hz), 119.5 (d, *J* = 3.5 Hz), 118.9 (d, *J* = 22.1 Hz), 114.3, 21.8, 20.9, 20.3.

2-Phenylquinazolin-4(3*H*)-one (5a) [6]: R_f = 0.4 (hexane:ethyl acetate 4:1); white solid; Yield

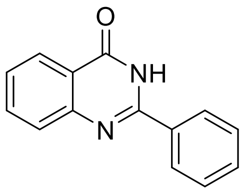

98% ; ^{1}H NMR (400 MHz, DMSO-d_6) δ 12.53 (s, 1H), 8.16 (s, 3H), 7.82 (d, *J* = 7.2 Hz, 1H), 7.74 (d, *J* = 7.6 Hz, 1H), 7.62 – 7.46 (m, 4H); ^{13}C NMR (100 MHz, DMSO-d_6) δ 162.4, 152.5, 148.8, 134.7, 132.8, 131.5, 128.7, 127.9, 127.5, 126.7, 125.9, 121.0.

2-(2-Bromophenyl)quinazolin-4(3*H*)-one (5b) [7]: R_f = 0.5 (hexane:ethyl acetate 7:3); white solid; Yield 86% ; ^{1}H NMR (400 MHz, DMSO-d_6) δ 12.61 (s, 1H), 8.20 – 8.13 (m, 1H), 7.84 (t, *J* = 7.6 Hz, 1H), 7.76 (d, *J* = 8.0 Hz, 1H), 7.70 (d, *J* = 8.0 Hz, 1H), 7.63 (dd, *J* = 7.6, 1.6 Hz, 1H), 7.57 (d, *J* = 7.6 Hz, 1H), 7.52 (d, *J* = 7.6 Hz, 1H), 7.48 (dd, *J* = 7.6, 1.6 Hz, 1H); ^{13}C NMR (100 MHz, DMSO-d_6) δ 161.5, 153.4, 148.6, 135.9, 134.7, 132.7, 131.8, 130.8, 127.8, 127.5, 127.1, 125.9, 121.3, 121.0.

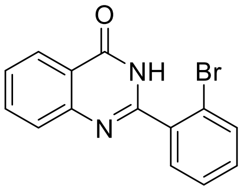

2-(2-Fluorophenyl)quinazolin-4(3*H*)-one (5c) [7]: R_f = 0.4 (hexane:ethyl acetate 4:1); white solid; Yield 88%; ^{1}H NMR (400 MHz, DMSO-d_6) δ 12.56 (s, 1H), 8.17 (d, *J* = 8.0 Hz, 1H), 7.85 (t, *J* = 7.6 Hz, 1H), 7.78 (td, *J* = 7.6, 1.6 Hz, 1H), 7.73 (d, *J* = 8.0 Hz, 1H), 7.61 (dd, *J* = 10.4, 4.8 Hz, 1H), 7.56 (t, *J* = 7.6 Hz, 1H), 7.40 (d, *J* = 9.2 Hz, 1H), 7.36 (d, *J* = 7.2 Hz, 1H); ^{13}C NMR (100 MHz, DMSO-d_6) δ 161.5, 159.6 (d, *J* = 250.3 Hz), 149.9, 148.7, 134.6, 132.9 (d, *J* = 8.5 Hz), 131.0 (d, *J* = 2.1 Hz), 127.5, 127.1, 125.9, 124.6 (d, *J* = 3.3 Hz), 122.3 (d, *J* = 13.1 Hz), 121.1, 116.2 (d, *J* = 21.3 Hz).

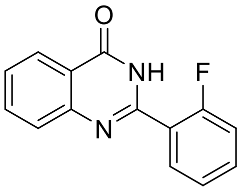

2-(3-Hydroxyphenyl)quinazolin-4(3*H*)-one (5d) [8]: R_f = 0.45 (hexane:ethyl acetate 7:3); white solid; Yield 78% ; ^{1}H NMR (400 MHz, DMSO-d_6) δ 12.41 (s, 1H), 9.74 (s, 1H), 8.14 (d, *J* = 8.0 Hz, 1H), 7.83 (t, *J* = 7.6 Hz, 1H), 7.71 (d, *J* = 8.0 Hz, 1H), 7.58 (d, *J* = 6.8 Hz, 2H), 7.51 (t, *J* = 7.6 Hz, 1H), 7.33 (t, *J* = 8.0 Hz, 1H), 6.97 (d, *J* = 7.6 Hz, 1H); ^{13}C NMR (100 MHz, DMSO-d_6) δ 162.2, 157.5, 152.3, 148.7, 134.6, 134.0, 129.7, 127.5, 126.6, 125.8, 120.9, 118.5, 118.3, 114.6.

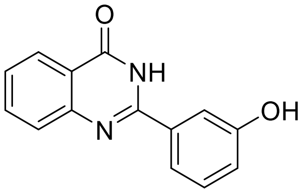

2-(4-Chlorophenyl)quinazolin-4(3*H*)-one (5e) [9]: R_f = 0.5 (hexane:ethyl acetate 7:3); white

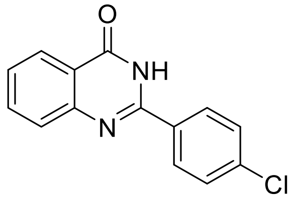

solid; Yield 92% ; ^{1}H NMR (400 MHz, DMSO-d$_6$) δ 12.60 (s, 1H), 8.20 (d, *J* = 8.4 Hz, 2H), 8.15 (d, *J* = 8.0 Hz, 1H), 7.84 (t, *J* = 7.2 Hz, 1H), 7.74 (d, *J* = 8.0 Hz, 1H), 7.62 (d, *J* = 8.4 Hz, 2H), 7.53 (t, *J* = 7.6 Hz, 1H); ^{13}C NMR (100 MHz, DMSO-d$_6$) δ 162.6, 151.8, 149.0, 136.8, 135.1, 132.0, 130.1, 129.2, 127.9, 127.2, 126.3, 121.5.

2-(4-Ethylphenyl)quinazolin-4(3*H*)-one (5f) [6]: R_f = 0.5 (hexane:ethyl acetate 7:3); white

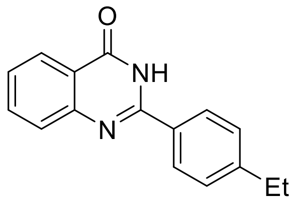

solid; Yield 94% ; ^{1}H NMR (400 MHz, DMSO-d$_6$) δ 12.44 (s, 1H), 8.14 (dd, *J* = 8.0, 1.2 Hz, 1H), 8.10 (d, *J* = 8.4 Hz, 2H), 7.85 – 7.79 (m, 1H), 7.72 (d, *J* = 8.0 Hz, 1H), 7.53 – 7.48 (m, 1H), 7.37 (d, *J* = 8.4 Hz, 2H), 2.68 (q, *J* = 7.6 Hz, 2H), 1.21 (t, *J* = 7.6 Hz, 3H); ^{13}C NMR (100 MHz, DMSO-d$_6$) δ 162.4, 152.4, 148.9, 147.7, 134.7, 130.2, 128.1, 127.9, 127.5, 126.5, 125.9, 120.9, 28.1, 15.4.

2-(*p*-Tolyl)quinazolin-4(3*H*)-one (5g) [10]: R_f = 0.45 (hexane:ethyl acetate 4:1); white solid;

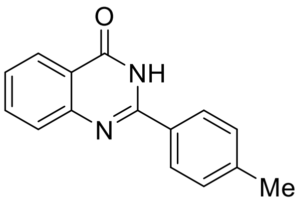

Yield 91% ; ^{1}H NMR (400 MHz, CDCl$_3$) δ 11.82 (s, 1H), 8.33 (d, *J* = 7.2 Hz, 1H), 8.17 (d, *J* = 7.2 Hz, 2H), 7.81 (s, 2H), 7.54 – 7.44 (m, 1H), 7.38 (d, *J* = 7.2 Hz, 2H), 2.46 (s, 3H); ^{13}C NMR (100 MHz, CDCl$_3$) δ 164.2, 151.9, 149.8, 142.3, 134.9, 130.1, 129.9, 128.0, 127.5, 126.7, 126.5, 120.9, 21.7.

2-(3,4,5-Trimethoxyphenyl)quinazolin-4(3*H*)-one (5h) [11]: R_f = 0.5 (hexane:ethyl acetate 7:3); white solid; Yield 86% ; ^{1}H NMR (400 MHz, $CDCl_3$) δ 11.81 (s, 1H), 8.25 (d, *J* = 8.0 Hz, 1H), 7.87 – 7.78 (m, 2H), 7.51 (d, *J* = 8.4 Hz, 3H), 4.05 (s, 6H), 3.96 (s, 3H); ^{13}C NMR (100 MHz, $CDCl_3$) δ 164.3, 153.8, 153.7, 151.8, 149.7, 141.3, 135.1, 128.2, 126.9, 126.3, 120.8, 104.9, 61.2, 56.6 (×2).

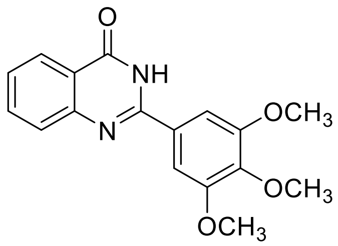

2-Mesitylquinazolin-4(3*H*)-one (5i) [12]: R_f = 0.5 (hexane:ethyl acetate 4:1); white solid; Yield 89% ; ^{1}H NMR (400 MHz, DMSO-d_6) δ 12.40 (s, 1H), 8.18 (d, *J* = 8.0 Hz, 1H), 7.83 (t, *J* = 7.6 Hz, 1H), 7.68 (d, *J* = 8.0 Hz, 1H), 7.54 (t, *J* = 7.6 Hz, 1H), 6.97 (s, 2H), 2.29 (s, 3H), 2.13 (s, 6H); ^{13}C NMR (100 MHz, DMSO-d_6) δ 161.8, 154.2, 148.9, 138.4, 135.4, 134.4, 131.9, 127.9, 127.3, 126.6, 125.8, 121.0, 20.7, 19.1 (×2).

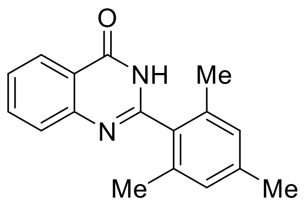

2-(Anthracen-9-yl)quinazolin-4(3*H*)-one (5j) [13]: R_f = 0.45 (hexane:ethyl acetate 7:3); white solid; Yield 85% ; ^{1}H NMR (400 MHz, $CDCl_3$) δ 9.28 (s, 1H), 8.62 (s, 1H), 8.39 (d, *J* = 8.0 Hz, 1H), 8.11 – 8.02 (m, 2H), 7.90 – 7.87 (m, 2H), 7.87 – 7.84 (m, 2H), 7.64 – 7.59 (m, 1H), 7.52 – 7.48 (m, 4H); ^{13}C NMR (100 MHz, $CDCl_3$) δ 161.9, 152.2, 149.1, 135.2, 131.3, 130.2, 129.8, 128.9, 128.3, 127.8, 127.7, 127.2, 126.8, 125.9, 124.6, 121.5.

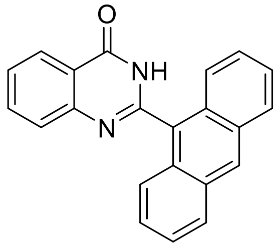

2-(Naphthalen-1-yl)quinazolin-4(3*H*)-one (5k) [7]: R_f = 0.4 (hexane:ethyl acetate 7:3); white solid; Yield 87% ; ^{1}H NMR (400 MHz, DMSO-d_6) δ 12.67 (s, 1H), 8.22 (d, *J* = 8.0 Hz, 1H), 8.15 (d, *J* = 8.0 Hz, 1H), 8.11 (d, *J* = 8.0 Hz, 1H), 8.04 (d, *J* = 8.0 Hz, 1H), 7.86 (t, *J* = 7.6 Hz, 1H), 7.79 (d, *J* = 7.2 Hz, 1H), 7.74 (d, *J* =

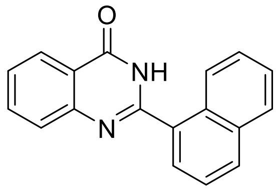

8.0 Hz, 1H), 7.65 (d, *J* = 7.6 Hz, 1H), 7.58 (dd, *J* = 8.0, 6.0 Hz, 3H); ^{13}C NMR (100 MHz, DMSO-d$_6$) δ 162.1, 153.8, 148.9, 134.8, 133.3, 131.8, 130.6, 130.4, 128.5, 127.8, 127.6, 127.3, 127.0, 126.6, 126.0, 125.3, 125.3, 121.3.

2-(4'-Chloro-[1,1'-biphenyl]-2-yl)quinazolin-4(3*H*)-one (5l) [14]: R$_f$ = 0.4 (hexane:ethyl acetate 4:1); white solid; Yield 93% ; ^{1}H NMR (400 MHz, CDCl$_3$) δ 9.85 (s, 1H), 8.18 (d, *J* = 8.0 Hz, 1H), 7.80 (t, *J* = 8.0 Hz, 2H), 7.75 (d, *J* = 8.0 Hz, 1H), 7.57 (d, *J* = 7.6 Hz, 1H), 7.51 (d, *J* = 7.2 Hz, 2H), 7.45 (d, *J* = 7.6 Hz, 1H), 7.44 - 7.35 (m, 1H), 7.24 – 7.17 (m, 3H); ^{13}C NMR (100 MHz, CDCl$_3$) δ 162.2, 153.5, 149.2, 139.5, 137.9, 134.9, 134.3, 132.9, 131.2, 130.9, 130.5, 130.4, 129.0, 128.4, 128.0, 127.2, 126.5, 120.8.

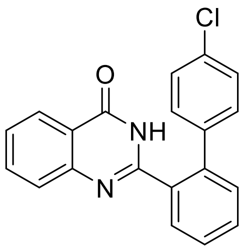

2-(2-Nitrophenyl)quinazolin-4(3*H*)-one (5m) [15]: R$_f$ = 0.3 (hexane:ethyl acetate 7:3); white solid; Yield 92% ; ^{1}H NMR (400 MHz, DMSO-d$_6$) δ 12.83 (s, 1H), 8.25 – 8.16 (m, 2H), 7.94 – 7.88 (m, 1H), 7.89 – 7.79 (m, 3H), 7.66 (d, *J* = 8.0 Hz, 1H), 7.58 (t, *J* = 7.6 Hz, 1H); ^{13}C NMR (100 MHz, DMSO-d$_6$) δ 161.5, 151.7, 148.6, 147.5, 134.7, 133.9, 131.6, 131.5, 129.2, 127.4, 127.2, 125.9, 124.6, 121.2.

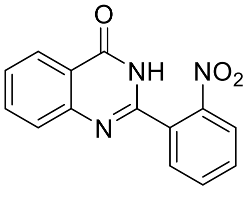

2-Propylquinazolin-4(3*H*)-one (5n) [16]: R$_f$ = 0.3 (hexane:ethyl acetate 7:3); white solid; Yield 79% ; ^{1}H NMR (400 MHz, CDCl$_3$) δ 12.07 (s, 1H), 8.29 (dd, *J* = 8.0, 1.2 Hz, 1H), 7.81 – 7.73 (m, 1H), 7.73 – 7.67 (m, 1H), 7.47 (dd, *J* = 80, 7.2 Hz, 1H), 2.92 – 2.43 (m, 2H), 2.00 – 1.64 (m, 2H), 1.08 (t, *J* = 7.6 Hz, 3H); ^{13}C NMR (100 MHz, CDCl$_3$) δ 164.6, 156.9, 149.6, 134.9, 127.4, 126.5, 126.4, 120.6, 37.9, 21.1, 13.9.

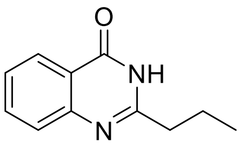

2-Cyclohexylquinazolin-4(3*H*)-one (5o) [16]: R_f = 0.4 (hexane:ethyl acetate 4:1); white solid; Yield 85% ; ^{1}H NMR (400 MHz, $CDCl_3$) δ 11.72 (s, 1H), 8.28 (dd, *J* = 8.0, 1.2 Hz, 1H), 7.75 (dd, *J* = 6.8, 1.6 Hz, 1H), 7.72 – 7.68 (m, 1H), 7.49 – 7.43 (m, 1H), 2.78 – 2.70 (m, 1H), 2.06 (d, *J* = 13.2 Hz, 2H), 1.98 – 1.86 (m, 2H), 1.84 – 1.78 (m, 2H), 1.74 (dd, *J* = 12.0, 2.8 Hz, 2H), 1.51 – 1.34 (m, 2H); ^{13}C NMR (100 MHz, $CDCl_3$) δ 164.4, 160.4, 149.7, 134.8, 127.5, 126.4, 126.3, 120.9, 45.0, 30.6, 26.2, 25.9.

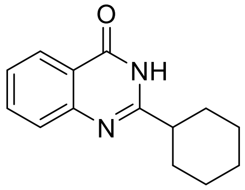

6-Fluoro-2-phenylquinazolin-4(3*H*)-one (6a) [17]: R_f = 0.5 (hexane:ethyl acetate 4:1); white solid; Yield 92% ; ^{1}H NMR (700 MHz, $CDCl_3$ + TFA-D) δ 8.08 (d, *J* = 7.7 Hz, 2H), 8.00 (dd, *J* = 9.1, 4.2 Hz, 1H), 7.95 (dd, *J* = 7.7, 2.8 Hz, 1H), 7.71 (t, *J* = 7.0 Hz, 1H), 7.69 – 7.65 (m, 1H), 7.63 (t, *J* = 7.7 Hz, 2H), 5.25 (s, 1H); ^{13}C NMR (175 MHz, $CDCl_3$ + TFA-D) δ 162.4, 161.6 (d, *J* = 249.7 Hz), 153.7, 141.4, 133.9, 129.7, 128.8, 127.9, 127.5 (d, *J* = 8.3 Hz), 125.5 (d, *J* = 24.4 Hz), 120.7 (d, *J* = 8.9 Hz), 112.2 (d, *J* = 24.2 Hz).

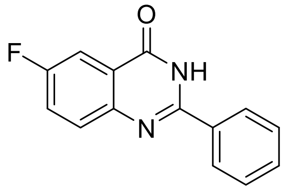

2-(2-Bromophenyl)-6-fluoroquinazolin-4(3*H*)-one (6b): R_f = 0.5 (hexane:ethyl acetate 4:1); white solid; Yield 89% ; mp 189−191 °C; ^{1}H NMR (700 MHz, $CDCl_3$ + TFA-d) δ 7.91 (dd, *J* = 8.4, 4.2 Hz, 1H), 7.88 (dd, *J* = 7.7, 2.8 Hz, 1H), 7.74 (d, *J* = 8.4 Hz, 1H), 7.67 (d, *J* = 7.7 Hz, 1H), 7.61 (td, *J* = 8.4, 2.8 Hz, 1H), 7.51 (t, *J* = 7.7 Hz, 1H), 7.45 (dd, *J* = 10.5, 4.2 Hz, 1H), 7.16 (s, 1H); ^{13}C NMR (175 MHz, $CDCl_3$ + TFA-d) δ 162.3, 161.5 (d, *J* = 247.6 Hz), 152.2, 144.1, 133.9, 133.1, 132.8, 131.1, 129.4 (d, *J* = 8.3 Hz), 128.1, 124.53 (d, *J* = 24.2 Hz), 121.66 (d, *J* = 8.9 Hz), 121.0, 111.76 (d, *J* = 23.9 Hz); IR (KBr) $\tilde{\nu}$ = 3379, 3172, 2921, 2852, 1676, 1621, 1605, 1478, 1350,

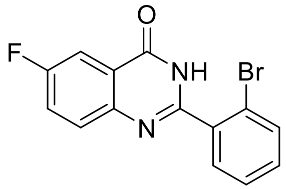

1305, 1253, 1229, 1124, 926, 830 cm^{-1}; HR-MS (ESI-TOF) m/z calcd for $C_{14}H_9BrFN_2O$ [M + H]$^+$ 318.9877, found 318.9875.

2-(4-Ethylphenyl)-6-fluoroquinazolin-4(3*H*)-one (6c): R_f = 0.5 (hexane:ethyl acetate 4:1); white solid; Yield 93% ; mp 243-245 °C; ^{1}H NMR (400 MHz, $CDCl_3$ + TFA-d) δ 8.04 – 7.98 (m, 3H), 7.91 (dd, *J* = 7.6, 2.8 Hz, 1H), 7.69 – 7.61 (m, 1H), 7.45 (d, *J* = 8.4 Hz, 2H), 5.07 (s, 1H), 2.76 (q, *J* = 7.6 Hz, 2H), 1.29 (t, *J* = 7.6 Hz, 3H); ^{13}C NMR (100 MHz, $CDCl_3$ + TFA-d) δ 161.9, 161.8 (d, *J* = 253.1 Hz), 154.4, 152.1, 140.3, 129.5, 128.3, 126.8 (d, *J* = 8.4 Hz), 125.7, 125.5, 120.7 (d, *J* = 8.9 Hz), 116.6, 113.7, 112.4 (d, *J* = 24.2 Hz), 29.1, 14.9; IR (KBr) $\tilde{\nu}$ = 3342, 3162, 3024, 2925, 1677, 1601, 1482, 1467, 1450, 1413, 1308, 1292, 1227, 1172, 1121, 1086, 889, 855 cm^{-1}; HR-MS (ESI-TOF) m/z calcd for $C_{16}H_{14}FN_2O$ [M + H]$^+$ 269.1085, found 269.1169.

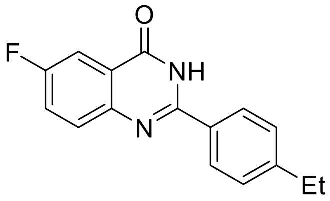

6-Fluoro-2-(*p*-tolyl)quinazolin-4(3*H*)-one (6d) [18]: R_f = 0.5 (hexane:ethyl acetate 4:1); white solid; Yield 88% ; ^{1}H NMR (700 MHz, $CDCl_3$) δ 10.66 (s, 1H), 8.03 (d, *J* = 4.9 Hz, 2H), 7.96 – 7.90 (m, 1H), 7.84 – 7.78 (m, 1H), 7.52 (dd, *J* = 10.5, 5.6 Hz, 1H), 7.38 (d, *J* = 4.9 Hz, 1H), 7.25 (d, *J* = 4.9 Hz, 1H), 2.46 (s, 3H); ^{13}C NMR (175 MHz, $CDCl_3$) δ 162.7, 160.9 (d, *J* = 248.4 Hz), 151.0, 146.3, 142.6, 130.4 (d, *J* = 7.8 Hz), 130.1, 129.8, 127.1, 123.6 (d, *J* = 24.0 Hz), 122.2 (d, *J* = 8.7 Hz), 111.5 (d, *J* = 23.6 Hz), 21.7.

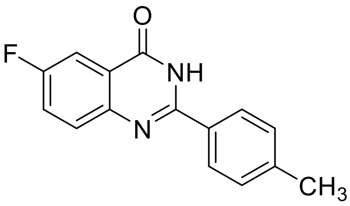

(*E*)-6-Fluoro-2-styrylquinazolin-4(3*H*)-one (6e) [19]: R_f = 0.6 (hexane:ethyl acetate 4:1); white solid; Yield 79% ; ^{1}H NMR (400 MHz, DMSO-d$_6$) δ 12.46 (s, 1H), 7.94 (d, *J* = 16.0 Hz, 1H), 7.80 – 7.77 (m, 1H), 7.76 – 7.73 (m, 1H), 7.71 (dd, *J* = 8.4, 2.8 Hz, 1H), 7.66 (d, *J* = 7.6 Hz, 2H), 7.50 – 7.39 (m, 3H), 7.00 (d, *J* = 16.0 Hz, 1H); ^{13}C NMR (100 MHz, DMSO-d$_6$) δ 161.2, 159.8 (d, *J* = 245.3 Hz), 150.9, 145.9, 138.3, 134.9, 129.9 (d, *J* = 8.3 Hz), 129.8, 129.1, 127.6, 123.0 (d, *J* = 24.0 Hz), 122.3, 120.9, 110.5 (d, *J* = 23.2 Hz).

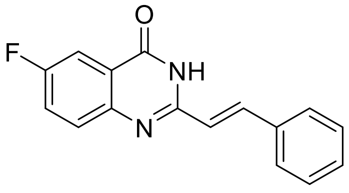

2-Cyclohexyl-6-fluoroquinazolin-4(3*H*)-one (6f): R_f = 0.5 (hexane:ethyl acetate 4:1); white solid; Yield 84% ; mp 236-237 °C; ^{1}H NMR (700 MHz, DMSO-d$_6$) δ 12.22 (s, 1H), 7.79 - 7.71 (m, 1H), 7.71 – 7.62 (m, 2H), 2.58 (d, *J* = 8.4 Hz, 1H), 1.90 (s, 2H), 1.79 (s, 2H), 1.68 (s, 1H), 1.63 – 1.50 (m, 2H), 1.30 (d, *J* = 9.1 Hz, 2H), 1.23 (d, *J* = 9.1 Hz, 1H); ^{13}C NMR (175 MHz, DMSO-d$_6$) δ 161.3, 160.3, 159.6 (d, *J* = 244.1 Hz), 145.8, 129.8, 129.7, 122.7 (d, *J* = 24.0 Hz), 122.1 (d, *J* = 8.1 Hz), 110.2 (d, *J* = 23.2 Hz), 42.7, 30.2, 25.5, 25.3; IR (KBr) $\tilde{\nu}$ = 2934, 2853, 1673, 1611, 1481, 1462, 1352, 1254, 1254, 1210, 1191, 979, 925 cm^{-1}; HR-MS (ESI-TOF) m/z calcd for $C_{14}H_{16}FN_2O$ $[M + H]^+$ 247.1241, found 247.1254.

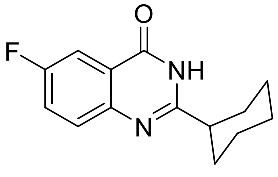

6-Fluoro-2-(4'-methoxy-[1,1'-biphenyl]-2-yl)quinazolin-4(3*H*)-one (6g): R_f = 0.4 (hexane:ethyl acetate 7:3); white solid; Yield 83% ; mp 200−201 °C; ^{1}H NMR (400 MHz, $CDCl_3$) δ 9.58 (s, 1H), 7.83 – 7.75 (m, 3H), 7.58 – 7.48 (m, 2H), 7.47 – 7.38 (m, 2H), 7.21 (d, *J* = 8.4 Hz, 2H), 6.77 (d, *J* = 8.4 Hz, 2H), 3.74 (s, 3H); ^{13}C NMR (100 MHz, $CDCl_3$) δ 161.3, 161.0 (d, *J* = 248.9 Hz), 159.6, 153.3, 145.9, 140.2, 132.3, 131.3, 131.2, 130.9, 130.5, 130.4 (d, *J* = 8.1 Hz), 130.3, 127.7, 123.4 (d, *J* = 24.1 Hz), 122.1 (d, *J* = 8.7 Hz), 114.4, 111.5

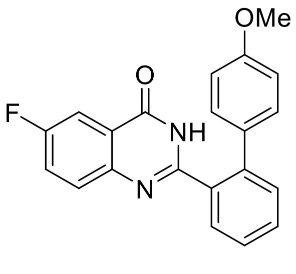

(d, J = 23.5 Hz), 55.3; IR (KBr) $\tilde{\nu}$ = 3359, 3028, 3001, 2929, 2838, 1685, 1667, 1599, 1515, 1483, 1461, 1439, 1304, 1247, 1176, 1142, 1128, 946, 877 cm^{-1}; HR-MS (ESI-TOF) m/z calcd for $C_{21}H_{15}FN_2O_2Na$ $[M + Na]^+$ 369.1010, found 369.1040.

2-(4'-Acetyl-[1,1'-biphenyl]-2-yl)-6-fluoroquinazolin-4(3*H*)-one (6h): R_f = 0.4 (hexane:ethyl acetate 7:3); white solid; Yield 85% ; mp 206−208 °C; ^{1}H NMR (400 MHz, CDCl$_3$) δ 10.81 (s, 1H), 7.82 – 7.66 (m, 5H), 7.58 (d, J = 7.2 Hz, 1H), 7.54 – 7.44 (m, 3H), 7.35 (d, J = 8.0 Hz, 2H), 2.49 (s, 3H); ^{13}C NMR (100 MHz, CDCl$_3$) δ 197.6, 161.1 (d, J = 249.2 Hz), 152.8, 145.8, 144.4, 139.8, 136.0, 132.6, 131.2, 130.9, 130.4 (d, J = 8.1 Hz), 130.3, 129.3, 128.7, 128.5, 123.8, 123.5, 121.8 (d, J = 8.7 Hz), 111.3 (d, J = 23.4 Hz), 26.7; IR (KBr) $\tilde{\nu}$ = 3152, 3110, 3001, 2836, 1662, 1603, 1590, 1478, 1440, 1298, 1283, 1266, 1253, 1133, 1003, 953, 828 cm^{-1}; HR-MS (ESI-TOF) m/z calcd for $C_{22}H_{15}FN_2O_2Na$ $[M + Na]^+$ 381.1010, found 381.1003.

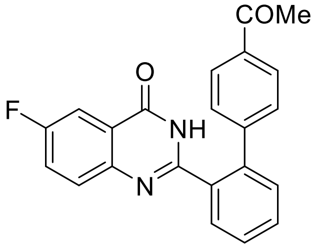

6-Chloro-2-phenylquinazolin-4(3*H*)-one (6i) [20]: R_f = 0.4 (hexane:ethyl acetate 4:1); white solid; Yield 96% ; ^{1}H NMR (700 MHz, DMSO-d$_6$) δ 12.70 (s, 1H), 8.17 (d, J = 7.7 Hz, 2H), 8.08 (d, J = 2.1 Hz, 1H), 7.85 (dd, J = 8.4, 2.1 Hz, 1H), 7.76 (d, J = 8.4 Hz, 1H), 7.59 (d, J = 7.0 Hz, 1H), 7.55 (t, J = 7.7 Hz, 2H); ^{13}C NMR (175 MHz, DMSO-d$_6$) δ 161.3, 152.9, 147.5, 134.7, 132.5, 131.6, 130.8, 129.2, 128.6, 127.9, 124.9, 122.2.

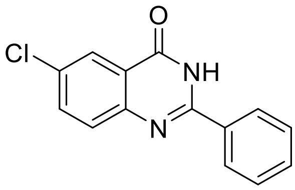

6-Chloro-2-(2-fluorophenyl)quinazolin-4(3*H*)-one (6j) [21]: R_f = 0.5 (hexane:ethyl acetate 4:1); white solid; Yield 91% ; ^{1}H NMR (400 MHz, $CDCl_3$ + DMSO-d_6) δ 12.06 (s, 1H), 9.50 (s, 1H), 9.43 (t, *J* = 7.2 Hz, 1H), 9.03 – 8.94 (m, 2H), 8.80 (dd, *J* = 12.4, 6.4 Hz, 1H), 8.64 – 8.55 (m, 1H), 8.48 (dd, *J* = 11.2, 8.4 Hz, 1H); ^{13}C NMR (100 MHz, $CDCl_3$ + DMSO-d_6) δ 161.1, 160.6 (d, *J* = 250.4 Hz), 149.1, 147.5, 135.1, 133.6 (d, *J* = 9.1 Hz), 132.9, 131.2, 129.6, 125.8, 125.2, 122.3, 120.4 (d, *J* = 10.6 Hz), 116.6 (d, *J* = 22.8 Hz).

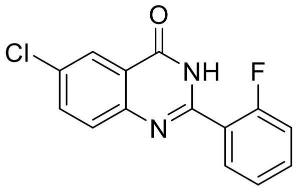

2-(2-Bromophenyl)-6-chloroquinazolin-4(3*H*)-one (6k) [22]: R_f = 0.5 (hexane:ethyl acetate 4:1); white solid; Yield 94% ; ^{1}H NMR (400 MHz, $CDCl_3$ + DMSO-d_6) δ 8.15 (s, 1H), 7.69 – 7.50 (m, 4H), 7.44 (s, 1H), 7.38 (d, *J* = 7.2 Hz, 1H), 7.32 (d, *J* = 7.2 Hz, 1H); ^{13}C NMR (100 MHz, $CDCl_3$ + DMSO-d_6) δ 160.9, 153.1, 146.9, 135.1, 134.2, 132.6, 132.0, 131.1, 130.2, 128.9, 127.2, 125.1, 122.2, 120.8.

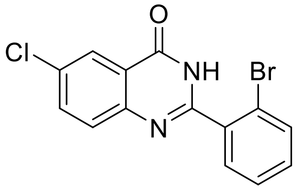

6-Chloro-2-(4-ethylphenyl)quinazolin-4(3*H*)-one (6l): R_f = 0.5 (hexane:ethyl acetate 4:1); white solid; Yield 89% ; mp 268−270 °C; ^{1}H NMR (400 MHz, DMSO-d_6) δ 12.66 (s, 1H), 8.11 (s, 1H), 8.09 (s, 1H), 8.07 (d, *J* = 2.0 Hz, 1H), 7.85 (dd, *J* = 8.8, 2.0 Hz, 1H), 7.74 (d, *J* = 8.8 Hz, 1H), 7.39 (d, *J* = 8.0 Hz, 2H), 2.69 (q, *J* = 7.6 Hz, 2H), 1.21 (t, *J* = 7.6 Hz, 3H); ^{13}C NMR (100 MHz, DMSO-d_6) δ 161.5, 152.9, 147.9, 147.6, 134.8, 130.7, 129.9, 129.7, 128.2, 127.9, 124.9, 122.2, 28.1, 15.4; IR (KBr) $\tilde{\nu}$ = 3174, 3104, 2959, 2868, 1660, 1596, 1554, 1474, 1463, 1308, 1289, 1191, 1114, 1077, 944, 897 cm^{-1}; HR-MS (ESI-TOF) m/z calcd for $C_{16}H_{13}ClN_2ONa$ $[M + Na]^+$ 307.0609, found 307.0602.

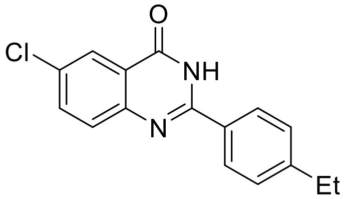

6-Chloro-2-propylquinazolin-4(3*H*)-one (6m) [23]: R_f = 0.5 (hexane:ethyl acetate 4:1); white solid; Yield 86% ; ^{1}H NMR (400 MHz, DMSO-d$_6$) δ 12.34 (s, 1H), 8.00 (d, *J* = 2.0 Hz, 1H), 7.78 (dd, *J* = 8.8, 2.4 Hz, 1H), 7.61 (d, *J* = 8.8 Hz, 1H), 2.57 (t, *J* = 7.6 Hz, 2H), 1.82 – 1.66 (m, 2H), 0.92 (t, *J* = 7.6 Hz, 3H); ^{13}C NMR (100 MHz, DMSO-d$_6$) δ 160.8, 157.9, 147.7, 134.4, 130.1, 129.1, 124.7, 122.0, 36.3, 20.1, 13.5.

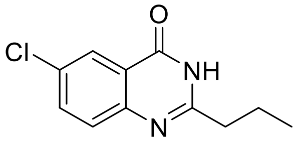

2-(4'-Acetyl-[1,1'-biphenyl]-2-yl)-6-chloroquinazolin-4(3*H*)-one (6n): R_f = 0.4 (hexane:ethyl acetate 7:3); white solid; Yield 79% ; mp 203−204 °C; ^{1}H NMR (400 MHz, CDCl$_3$) δ 10.35 (s, 1H), 8.08 (d, *J* = 1.2 Hz, 1H), 7.79 (t, *J* = 10.0 Hz, 3H), 7.71 (dd, *J* = 8.8, 2.0 Hz, 1H), 7.67 – 7.59 (m, 2H), 7.56 – 7.48 (m, 2H), 7.37 (d, *J* = 8.0 Hz, 2H), 2.52 (s, 3H); ^{13}C NMR (100 MHz, CDCl$_3$) δ 197.6, 161.6, 153.6, 147.6, 144.3, 139.7, 136.2, 135.5, 133.1, 132.6, 131.4, 130.9, 130.3, 129.7, 129.3, 128.8, 128.7, 125.8, 121.7, 26.7; IR (KBr) $\tilde{\nu}$ = 3162, 3095, 3004, 2920, 1664, 1602, 1586, 1570, 1493, 1461, 1301, 1285, 1266, 1188, 1159, 1023, 960, 942 cm^{-1}; HR-MS (ESI-TOF) m/z calcd for $C_{22}H_{15}ClN_2O_2Na$ [M + Na]$^+$ 397.0714, found 397.0932.

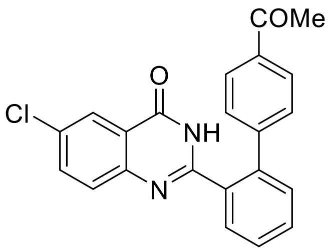

6-Chloro-2-(4'-methoxy-[1,1'-biphenyl]-2-yl)quinazolin-4(3*H*)-one (6o): R_f = 0.4 (hexane:ethyl acetate 7:3); white solid; Yield 82% ; mp 178−180 °C; ^{1}H NMR (400 MHz, CDCl$_3$) δ 9.92 (s, 1H), 8.10 (s, 1H), 7.76 (d, *J* = 7.6 Hz, 1H), 7.71 (s, 2H), 7.52 (t, *J* = 7.2 Hz, 1H), 7.47 – 7.35 (m, 2H), 7.19 (t, *J* = 8.8 Hz, 2H), 6.74 (t, *J* = 8.0 Hz, 2H), 3.72 (s, 3H); ^{13}C NMR (100 MHz, CDCl$_3$) δ 161.2, 159.5, 154.3, 147.8, 140.3, 135.2, 132.8, 132.2, 131.3, 131.2, 130.9, 130.4, 130.3, 129.6, 127.6, 125.9, 121.9, 114.3, 55.3 IR (KBr) $\tilde{\nu}$ = 3153,

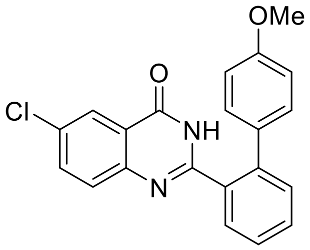

3.33, 2952, 2834, 1665, 1604, 1586, 1492, 1462, 1301, 1237, 1179, 1121, 1032, 963, 942 cm^{-1}; HR-MS (ESI-TOF) m/z calcd for $C_{21}H_{15}ClN_2O_2Na$ $[M + Na]^+$ 385.0714, found 385.0695.

2-(Anthracen-9-yl)-6-chloroquinazolin-4(3*H*)-one (6p): R_f = 0.4 (hexane:ethyl acetate 4:1);

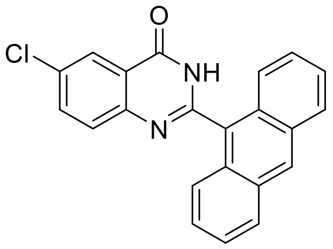

yellowish white solid; Yield 96% ; mp 244−245 °C; ^1H NMR (400 MHz, $CDCl_3$) δ 9.73 (s, 1H), 8.59 (s, 1H), 8.19 (s, 1H), 8.09 – 7.97 (m, 2H), 7.84 – 7.74 (m, 4H), 7.52 – 7.43 (m, 4H); ^{13}C NMR (100 MHz, $CDCl_3$) δ 161.2, 152.5, 147.6, 135.6, 133.6, 131.2, 130.3, 129.9, 129.7, 129.0, 127.7, 126.8, 126.2, 125.9, 124.5, 122.5; IR (KBr) $\tilde{\nu}$ = 3148, 2921, 2851, 1664, 1596, 1479, 1462, 1377, 1261, 1241, 1169, 1151, 1016, 905, 838 cm^{-1}; HR-MS (ESI-TOF) m/z calcd for $C_{22}H_{14}ClN_2O$ $[M + H]^+$ 357.0789, found 357.0780.

6-Chloro-2-(2-fluorophenyl)quinazolin-4(3*H*)-one (6q) [21]: R_f = 0.3 (hexane:ethyl acetate 7:3); white solid; Yield 93% ; ^1H NMR (400 MHz, DMSO-d_6) δ 12.74 (s, 1H), 8.08 (d, *J* = 2.4

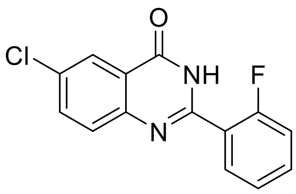

Hz, 1H), 7.85 (dd, *J* = 8.8, 2.4 Hz, 1H), 7.76 – 7.65 (m, 2H), 7.67 – 7.56 (m, 1H), 7.43 – 7.32 (m, 2H); ^{13}C NMR (100 MHz, DMSO-d_6) δ 160.6, 159.6 (d, *J* = 250.7 Hz), 150.5, 147.4, 134.7, 133.1 (d, *J* = 8.5 Hz), 131.3, 131.1, 129.8, 124.9, 124.7 (d, *J* = 3.3 Hz), 122.4, 122.0 (d, *J* = 13.0 Hz), 116.3 (d, *J* = 21.2 Hz).

6-Chloro-2-(2-nitrophenyl)quinazolin-4(3*H*)-one (6r): R_f = 0.4 (hexane:ethyl acetate 7:3); white solid; Yield 94%; mp 274−276 °C; ^1H NMR (400 MHz, DMSO-d_6) δ 13.02 (s, 1H), 8.22

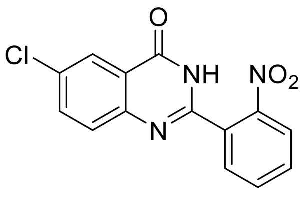

(d, *J* = 8.0 Hz, 1H), 8.12 (d, *J* = 2.4 Hz, 1H), 7.94 – 7.88 (m, 2H), 7.88 – 7.80 (m, 2H), 7.69 (d, *J* = 8.8 Hz, 1H); ^{13}C NMR (100 MHz, DMSO-d_6) δ 161.0, 152.7, 147.8, 147.7, 135.3, 134.5, 132.2, 131.9, 131.9, 130.1, 129.4, 125.4, 125.1, 122.9; IR (KBr) $\tilde{\nu}$ = 3026, 2870, 2797, 1668, 1623, 1527,

1350, 1175, 853 cm^{-1}; HR-MS (ESI-TOF) m/z calcd for $C_{14}H_8ClN_3O_3Na$ [M + Na]$^+$ 324.0146, found 324.0175.

6-Fluoro-2-(2-nitrophenyl)quinazolin-4(3*H*)-one (6s): R$_f$ = 0.3 (hexane:ethyl acetate 7:3); white solid; Yield 89%; mp 246−248 °C; ^{1}H NMR (400 MHz, DMSO-d$_6$) δ 12.89 (s, 1H), 8.20

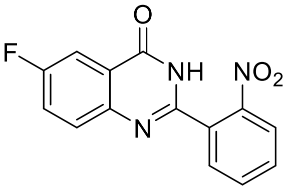

(d, *J* = 8.0 Hz, 1H), 7.92– 7.86 (m, 2H), 7.85 – 7.79 (m, 2H), 7.74 (s, 1H), 7.72 (s, 1H); ^{13}C NMR (100 MHz, DMSO-d$_6$) δ 161.4, 160.3 (d, *J* = 246.1 Hz), 151.6, 147.5, 145.5, 133.9, 131.6, 131.6, 130.2 (d, *J* = 8.3 Hz), 129.3, 124.6, 123.1 (d, *J* = 24.1 Hz), 122.5 (d, *J* = 8.3 Hz), 110.7 (d, *J* = 23.3 Hz) ; IR (KBr) $\tilde{\nu}$ = 3011, 2874, 1668, 1621, 1528, 1254, 949 cm^{-1}; HR-MS (ESI-TOF) m/z calcd for $C_{14}H_8FN_3O_3Na$ [M + Na]$^+$ 308.0442, found 308.0455.

2-(4-Bromophenyl)quinazolin-4(3*H*)-one (5m) [9]: R$_f$ = 0.5 (hexane:ethyl acetate 4:1); white

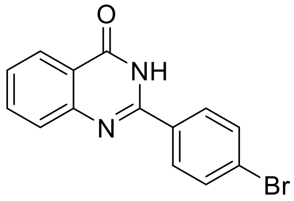

solid; Yield 95% ; ^{1}H NMR (400 MHz, DMSO-d$_6$) δ 12.61 (s, 1H), 8.14 (t, *J* = 8.0 Hz, 3H), 7.83 (t, *J* = 7.6 Hz, 1H), 7.74 (t, *J* = 8.0 Hz, 3H), 7.52 (t, *J* = 7.6 Hz, 1H); ^{13}C NMR (100 MHz, DMSO-d$_6$) δ 162.9, 152.2, 149.1, 135.0, 132.1, 131.7, 130.3, 127.9, 127.2, 126.4, 125.6, 121.5.

References

1. Deo, C.; Bogliotti, N.; Métivier, R.; Retailleau, P.; Xie, J. *Organometallics* **2015,** *34*, 5775-5784. doi: 10.1021/acs.organomet.5b00871.

2. SAINT+, Bruker AXS Inc., Madison, Wisconsin, USA, 1999 (Program for Reduction of Data collected on Bruker CCD Area Detector Diffractometer V. 6.02.)

3. Sheldrick, G. In *SADABS, Program for Empirical Absorption Correction of Area Detector Data*, 1996.

4. Sheldrick, G. *Acta Crystallogr. Sect. A* **2008,** *64*, 112-122. doi: doi:10.1107/S0108767307043930.

5. Maiti, S.; Mal, P. *Adv. Synth. Catal.* **2015,** *357*, 1416-1424. doi: 10.1002/adsc.201401110.

6. Huang, J.; Chen, W.; Liang, J.; Yang, Q.; Fan, Y.; Chen, M.-W.; Peng, Y. *J. Org. Chem.* **2021,** *86*, 14866-14882. doi: 10.1021/acs.joc.1c01497.

7. Sahoo, S.; Pal, S. *J. Org. Chem.* **2021,** *86*, 18067-18080. doi: 10.1021/acs.joc.1c02343.

8. Karuturi, R.; Al-Horani, R. A.; Mehta, S. C.; Gailani, D.; Desai, U. R. *J. Med. Chem.* **2013,** *56*, 2415-2428. doi: 10.1021/jm301757v.

9. Rohokale, R. S.; Kalshetti, R. G.; Ramana, C. V. *J. Org. Chem.* **2019,** *84*, 2951-2961. doi: 10.1021/acs.joc.8b02738.

10. Das, S.; Sinha, S.; Samanta, D.; Mondal, R.; Chakraborty, G.; Brandaõ, P.; Paul, N. D. *J. Org. Chem.* **2019,** *84*, 10160-10171. doi: 10.1021/acs.joc.9b01343.

11. Upadhyaya, K.; Thakur, R. K.; Shukla, S. K.; Tripathi, R. P. *J. Org. Chem.* **2016,** *81*, 5046-5055. doi: 10.1021/acs.joc.6b00599.

12. Laha, J. K.; Satyanarayana Tummalapalli, K. S.; Jethava, K. P. *Org. Biomol. Chem.* **2016,** *14*, 2473-2479. doi: 10.1039/C5OB02670A.

13. Parua, S.; Das, S.; Sikari, R.; Sinha, S.; Paul, N. D. *J. Org. Chem.* **2017,** *82*, 7165-7175. doi: 10.1021/acs.joc.7b00643.

14. Banerji, B.; Bera, S.; Chatterjee, S.; Killi, S. K.; Adhikary, S. *Chem. Eur. J.* **2016,** *22*, 3506-3512. doi: 10.1002/chem.201504186.

15. Ghosh, T.; Mandal, I.; Basak, S. J.; Dash, J. *J. Org. Chem.* **2021,** *86*, 14695-14704. doi: 10.1021/acs.joc.1c01510.

16. Wang, K.; Chen, H.; Dai, X.; Huang, X.; Feng, Z. *RSC Adv.* **2021,** *11*, 13119-13123. doi: 10.1039/D1RA01755A.

17. Hernández, J. G.; Bolm, C. *J. Org. Chem.* **2017,** *82*, 4007-4019. doi: 10.1021/acs.joc.6b02887.

18. Dong, Y.; Zhang, J.; Yang, J.; Yan, C.; Wu, Y. *New J. Chem.* **2021,** *45*, 15344-15349. doi: 10.1039/D1NJ03179A.

19. Jiang, J. B.; Hesson, D. P.; Dusak, B. A.; Dexter, D. L.; Kang, G. J.; Hamel, E. *J. Med. Chem.* **1990,** *33*, 1721-1728. doi: 10.1021/jm00168a029.

20. Jia, F.-C.; Zhou, Z.-W.; Xu, C.; Wu, Y.-D.; Wu, A.-X. *Org. Lett.* **2016,** *18,* 2942-2945. doi: 10.1021/acs.orglett.6b01291.

21. Kumar, M.; Richa; Sharma, S.; Bhatt, V.; Kumar, N. *Adv. Synth. Catal.* **2015,** *357*, 2862-2868. doi: https://doi.org/10.1002/adsc.201500335.

22. Liu, J.-Q.; Ma, Y.-G.; Zhang, M.-M.; Wang, X.-S. *J. Org. Chem.* **2017,** *82*, 4918-4923. doi: 10.1021/acs.joc.7b00259.

23. Xu, W.; Fu, H. *J. Org. Chem.* **2011,** *76*, 3846-3852. doi: 10.1021/jo2002227.

Copies of NMR spectra

^{1}H NMR (700 MHz, CDCl$_3$)

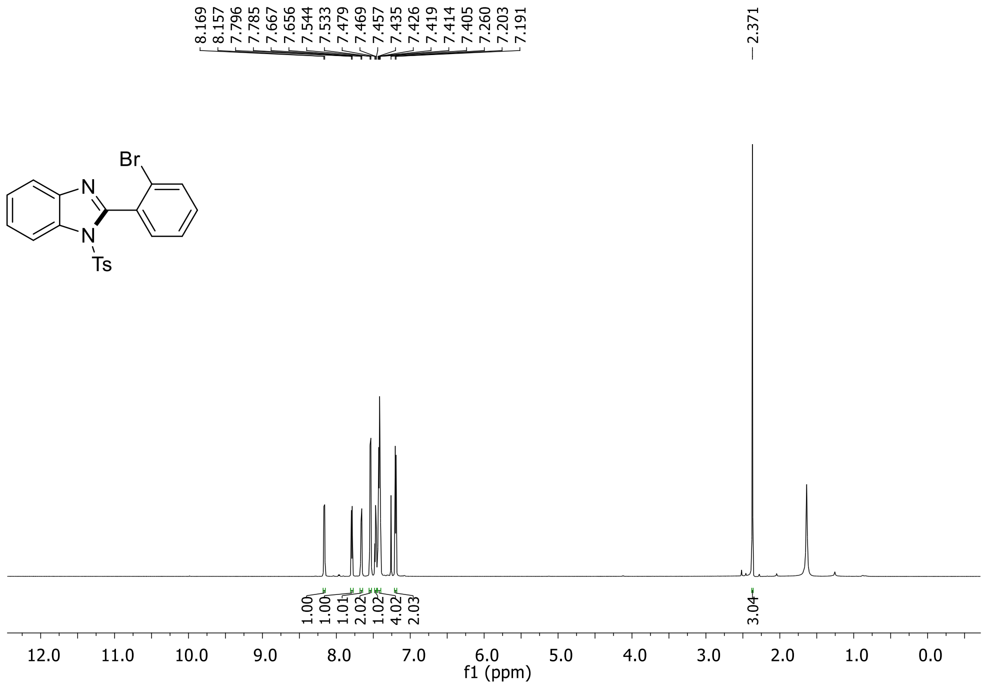

Figure S2. ^{1}H NMR of 2-(2-bromophenyl)-1-tosyl-1*H*-benzo[*d*]imidazole **(2a)**

^{13}C NMR (175 MHz, CDCl$_3$)

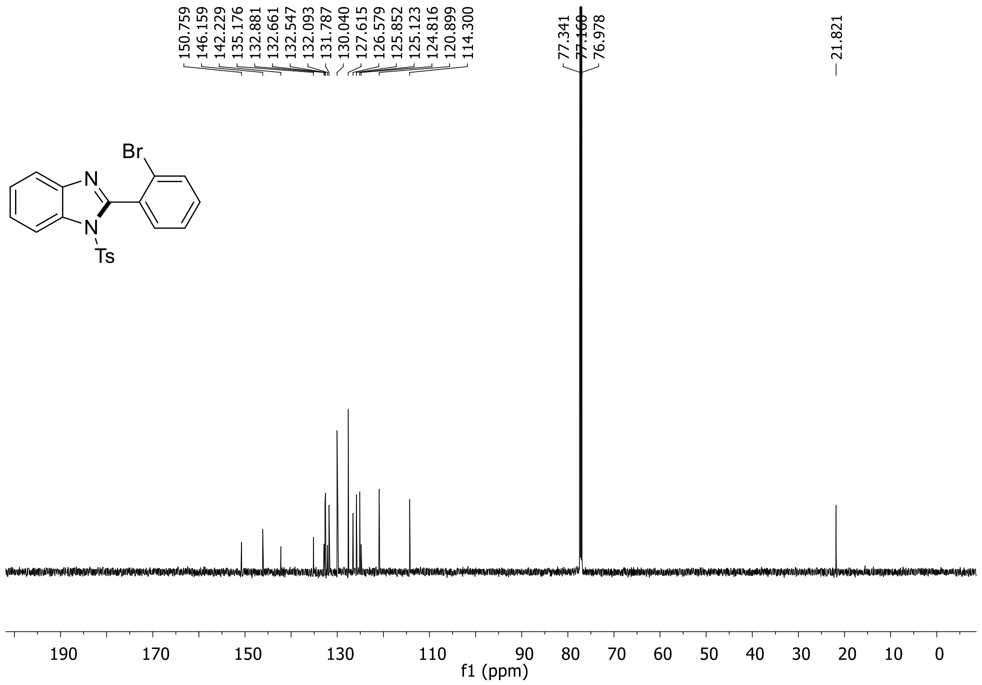

Figure S3. ^{13}C NMR of 2-(2-bromophenyl)-1-tosyl-1*H*-benzo[*d*]imidazole **(2a)**

^{1}H NMR (400 MHz, CDCl$_3$)

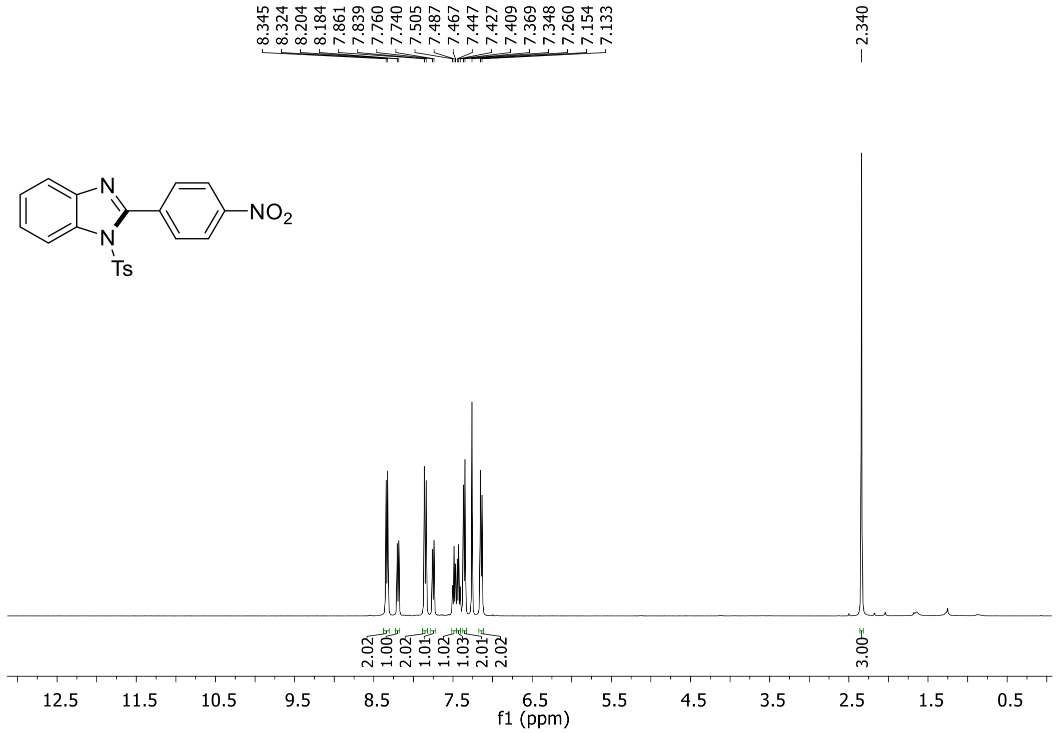

Figure S4. ^{1}H NMR of 2-(4-nitrophenyl)-1-tosyl-1*H*-benzo[*d*]imidazole **(2b)**

^{13}C NMR (100 MHz, CDCl$_3$)

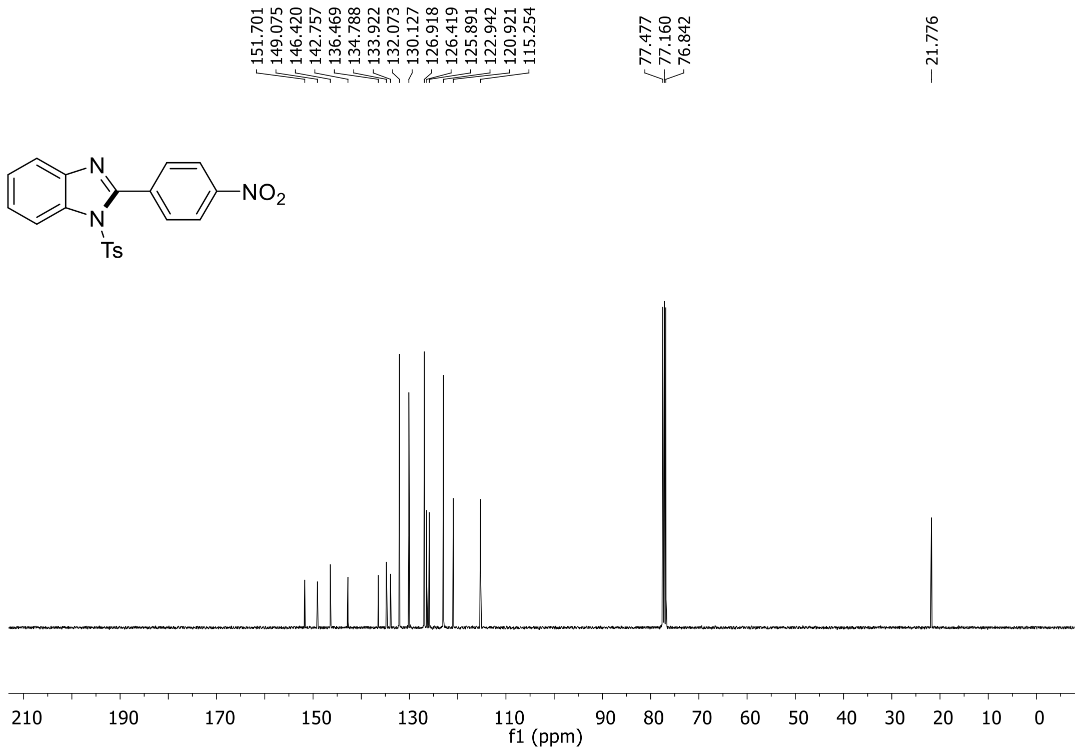

Figure S5. ^{13}C NMR of 2-(4-nitrophenyl)-1-tosyl-1*H*-benzo[*d*]imidazole **(2b)**

^{1}H **NMR (400 MHz, $CDCl_3$)**

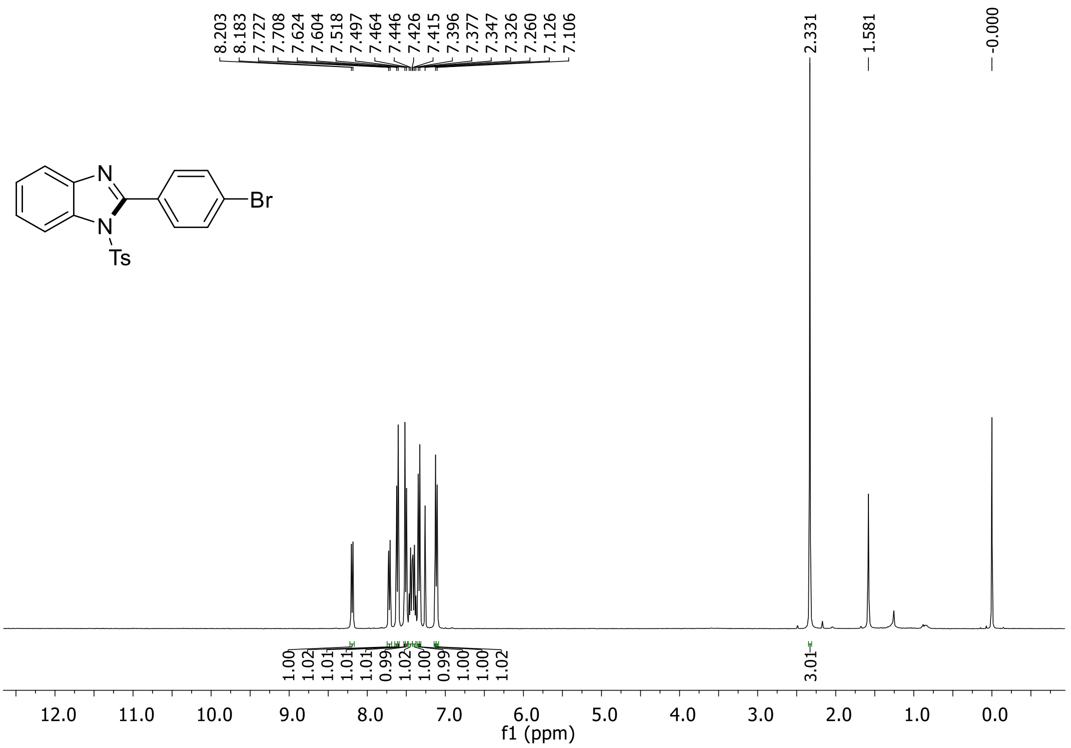

Figure S6. ^{1}H NMR of 2-(4-bromophenyl)-1-tosyl-1*H*-benzo[*d*]imidazole **(2c)**

^{13}C **NMR (100 MHz, $CDCl_3$)**

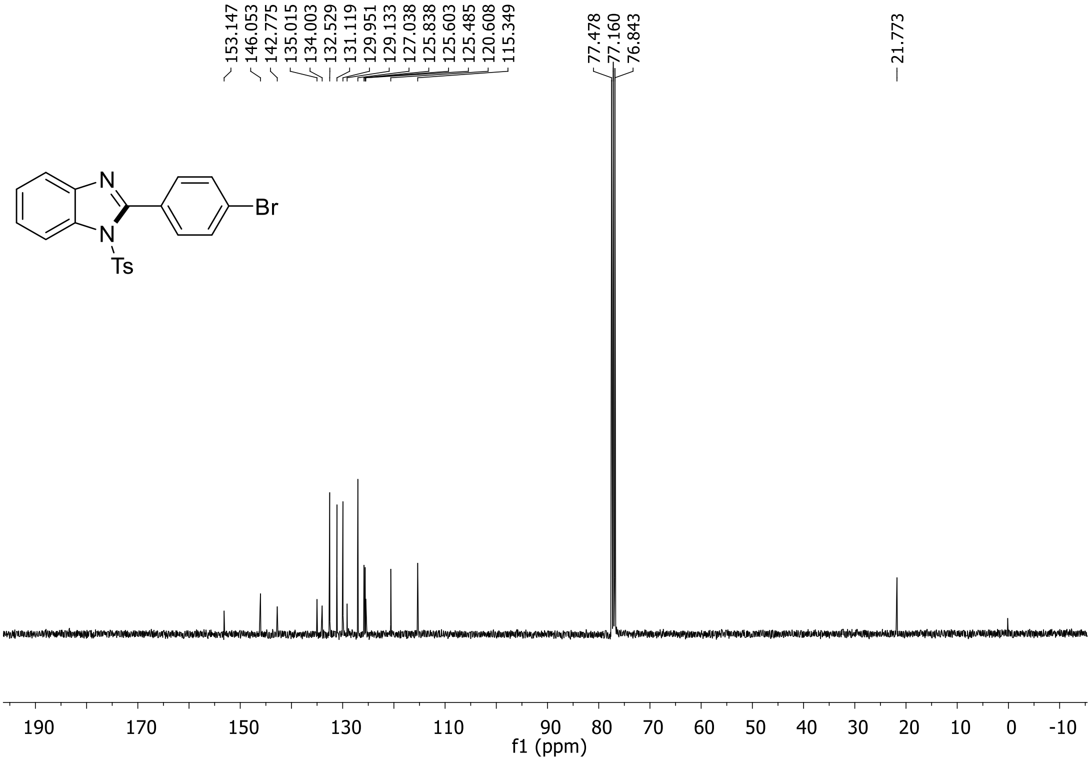

Figure S7. ^{13}C NMR of 2-(4-bromophenyl)-1-tosyl-1*H*-benzo[*d*]imidazole **(2c)**

^{1}H NMR (400 MHz, $CDCl_3$)

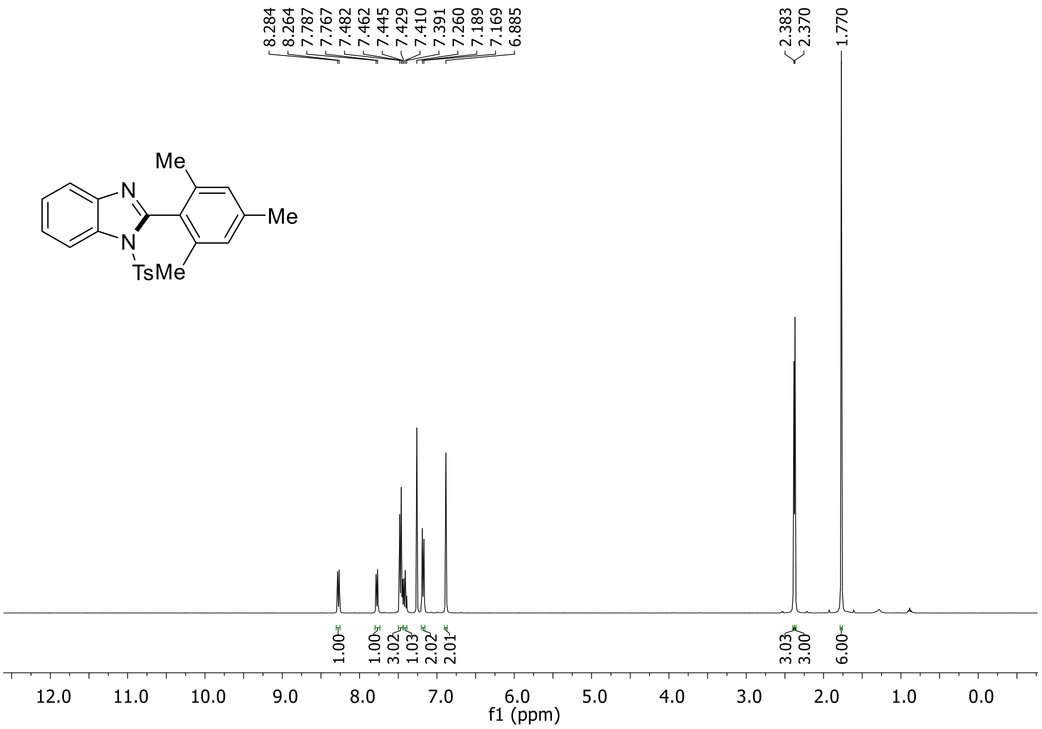

Figure S8. ^{1}H NMR of 2-mesityl-1-tosyl-1*H*-benzo[*d*]imidazole **(2d)**

^{13}C NMR (100 MHz, $CDCl_3$)

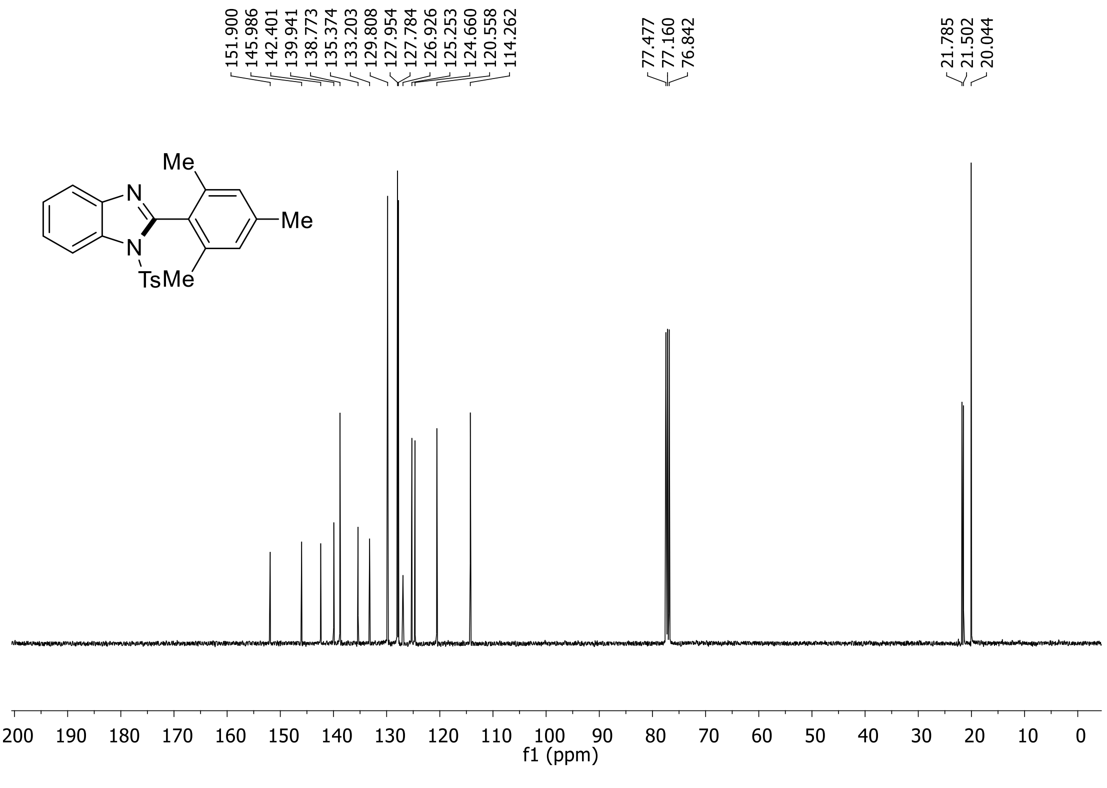

Figure S9. ^{13}C NMR of 2-mesityl-1-tosyl-1*H*-benzo[*d*]imidazole **(2d)**

^{1}H NMR (400 MHz, $CDCl_3$)

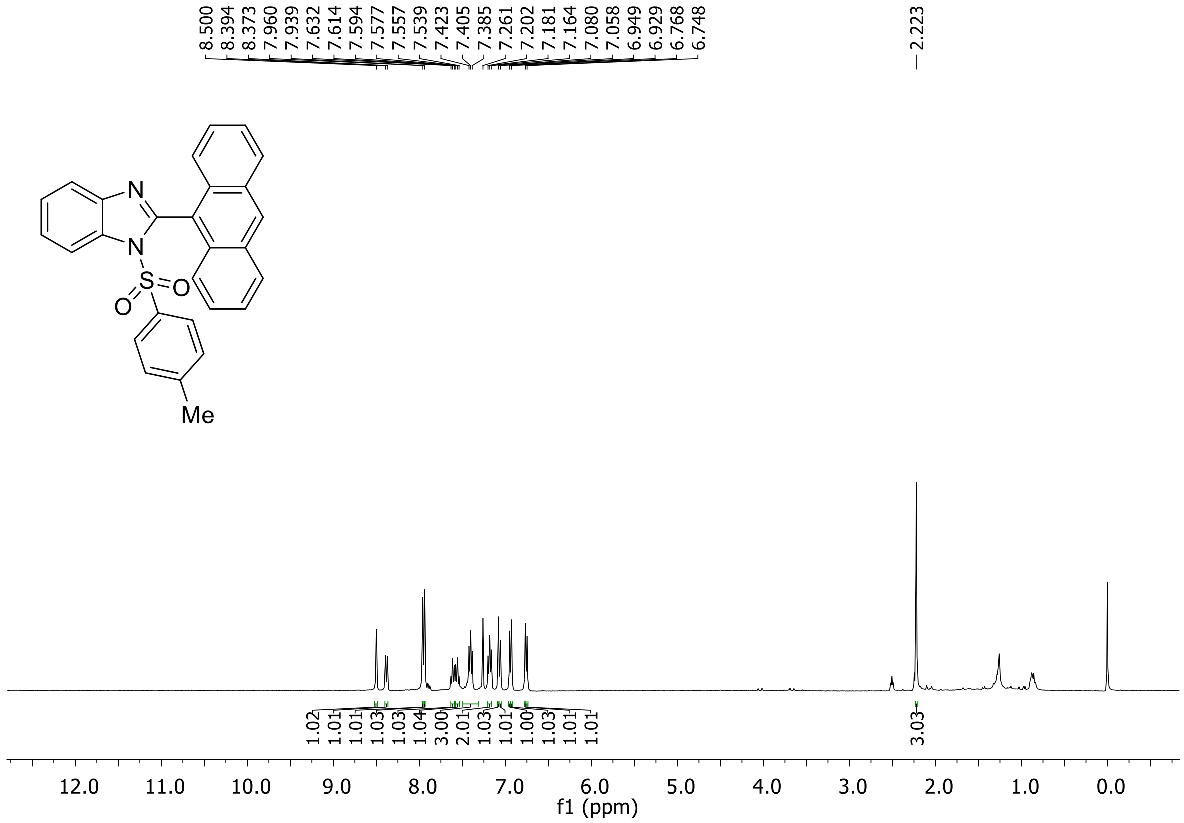

Figure S10. ^{1}H NMR of 2-(anthracen-9-yl)-1-tosyl-1*H*-benzo[*d*]imidazole **(2e)**

^{13}C NMR (100 MHz, $CDCl_3$)

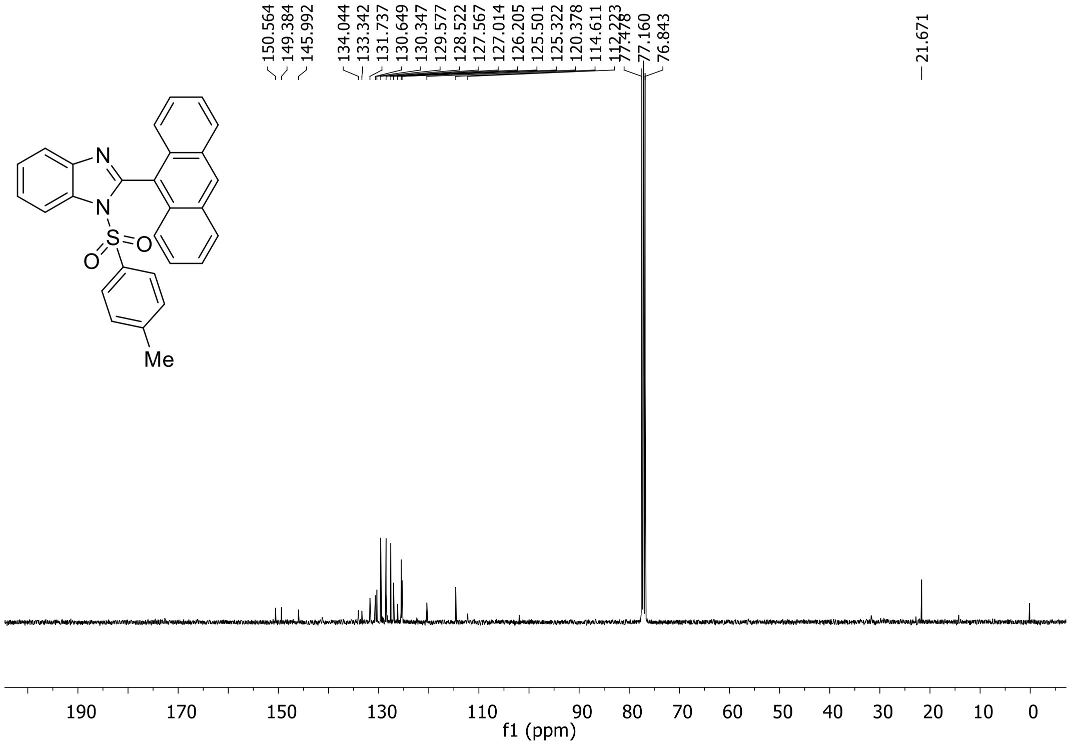

Figure S11. ^{13}C NMR of 2-(anthracen-9-yl)-1-tosyl-1*H*-benzo[*d*]imidazole **(2e)**

^{1}H NMR (400 MHz, $CDCl_3$)

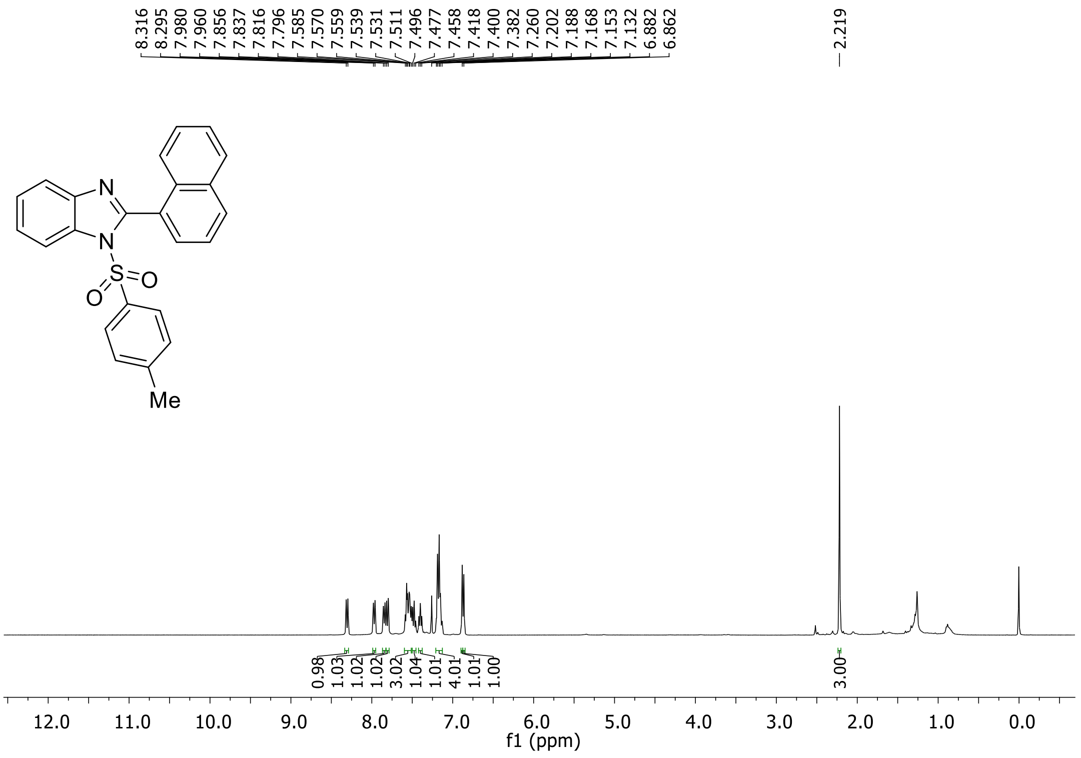

Figure S12. ^{1}H NMR of 2-(naphthalen-1-yl)-1-tosyl-1*H*-benzo[*d*]imidazole **(2f)**

^{13}C NMR (100 MHz, $CDCl_3$)

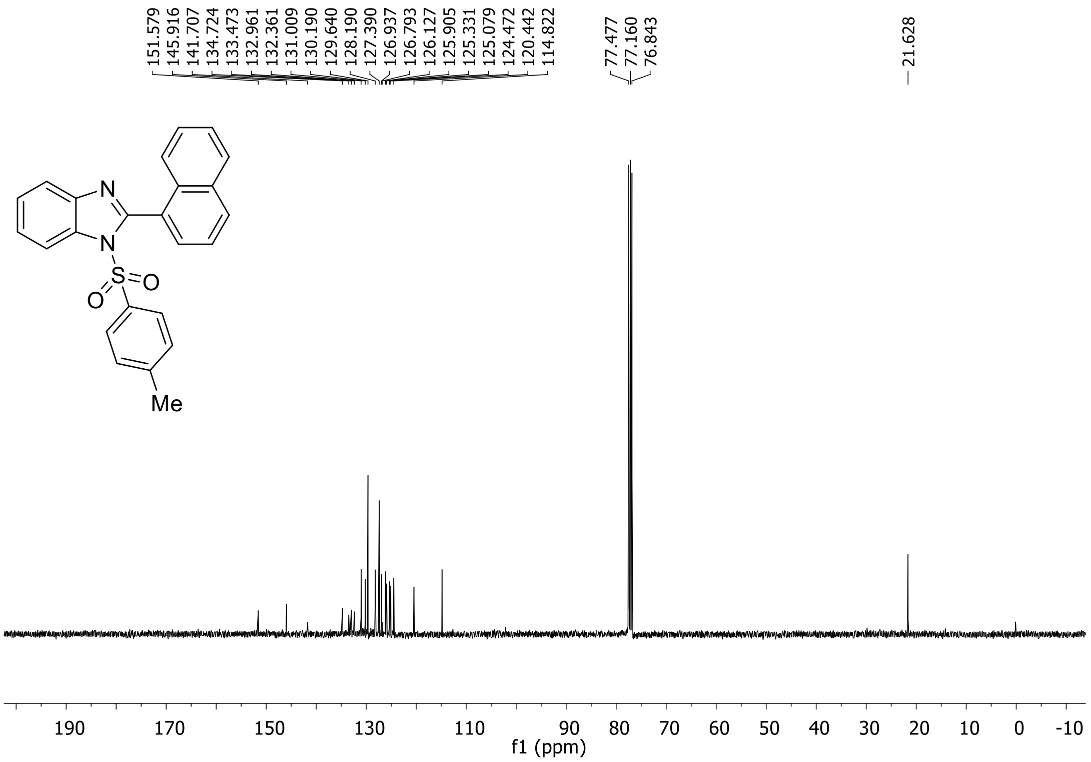

Figure S13. ^{13}C NMR of 2-(naphthalen-1-yl)-1-tosyl-1*H*-benzo[*d*]imidazole **(2f)**

^{1}H NMR (400 MHz, CDCl$_3$)

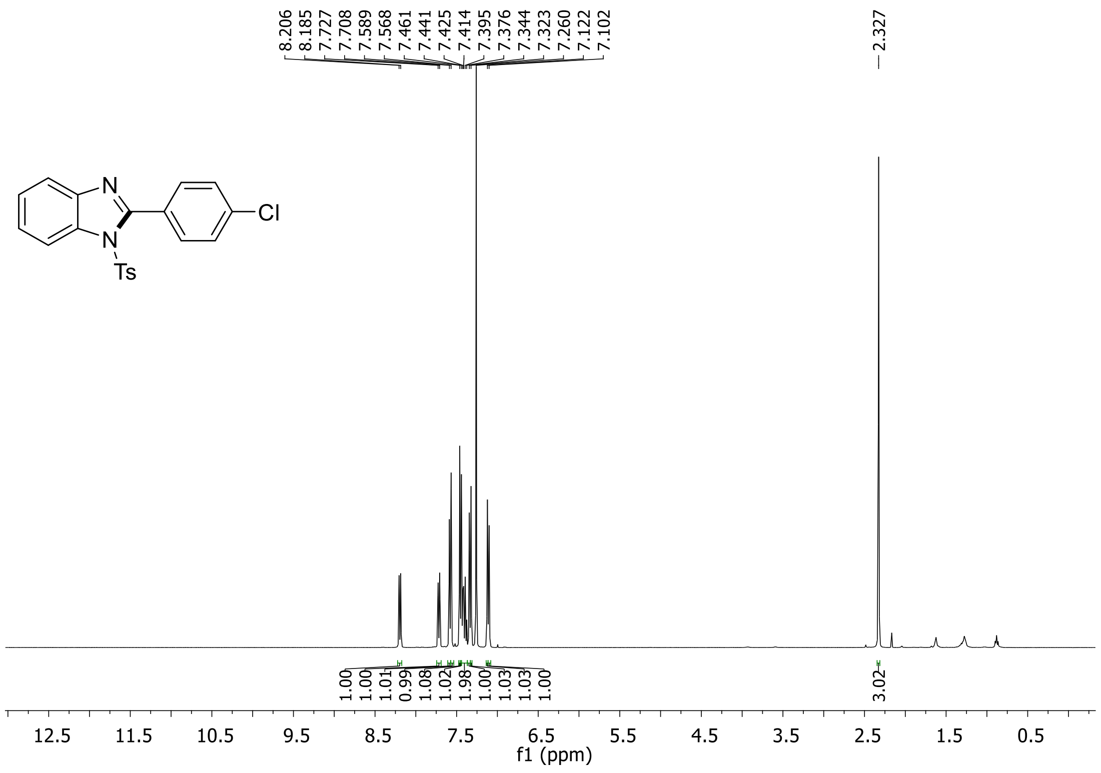

Figure S14. ^{1}H NMR of 2-(4-chlorophenyl)-1-tosyl-1*H*-benzo[*d*]imidazole **(2g)**

^{13}C NMR (100 MHz, CDCl$_3$)

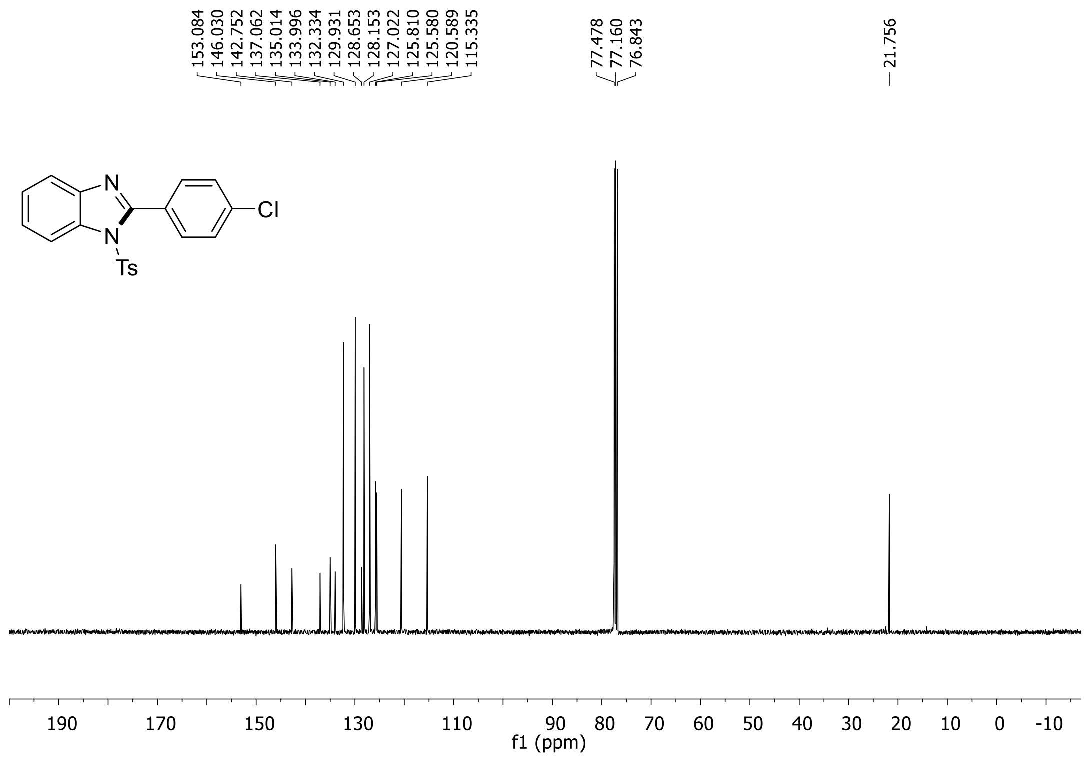

Figure S15. ^{13}C NMR of 2-(4-chlorophenyl)-1-tosyl-1*H*-benzo[*d*]imidazole **(2g)**

^{1}H NMR (400 MHz, $CDCl_3$)

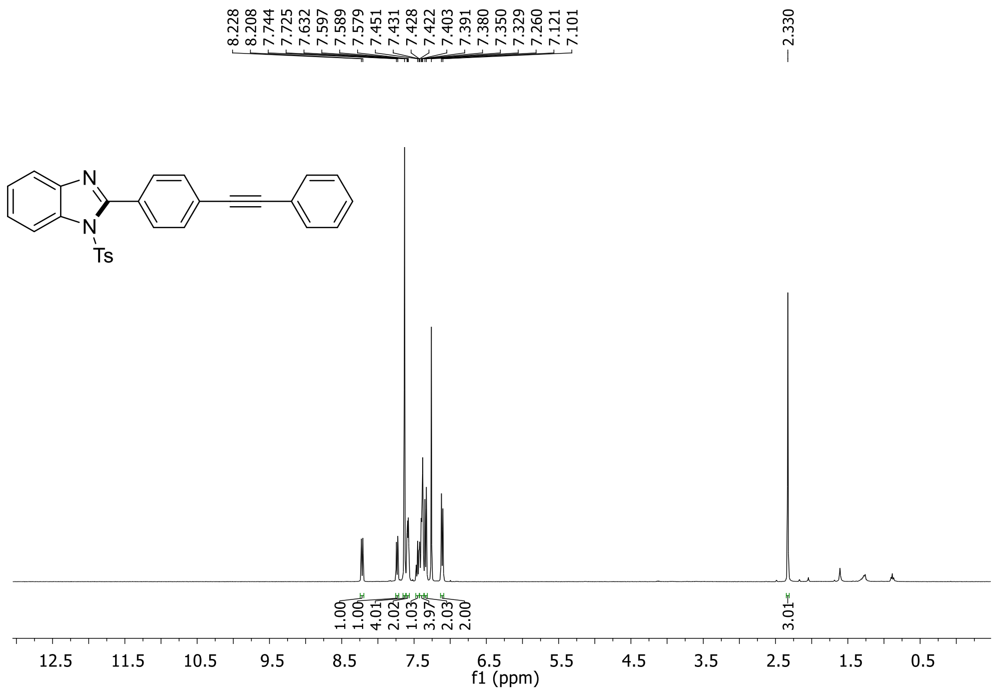

Figure S16. ^{1}H NMR of 2-(4-(phenylethynyl)phenyl)-1-tosyl-1*H*-benzo[*d*]imidazole **(2h)**

^{13}C NMR (100 MHz, $CDCl_3$)

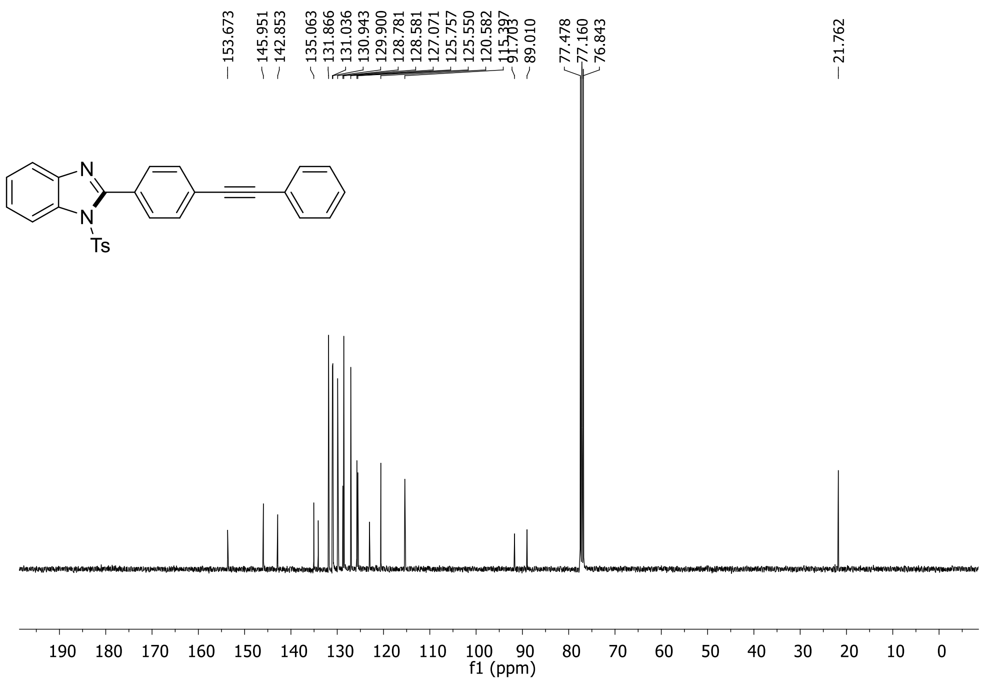

Figure S17. ^{13}C NMR of 2-(4-(phenylethynyl)phenyl)-1-tosyl-1*H*-benzo[*d*]imidazole **(2h)**

^{1}H NMR (400 MHz, CDCl$_3$)

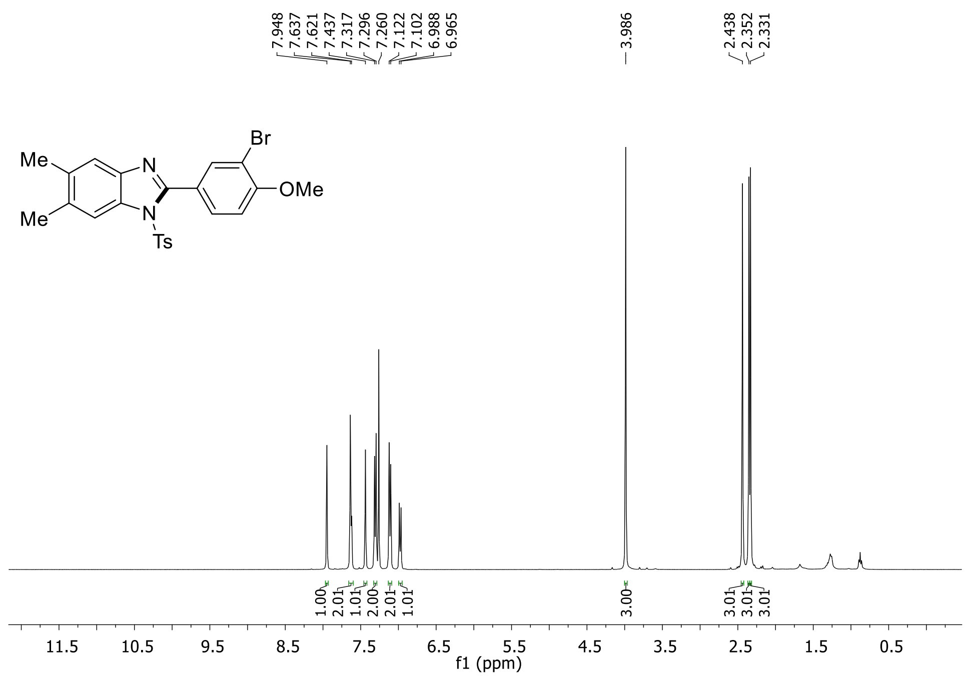

Figure S18. ^{1}H NMR of 2-(3-bromo-4-methoxyphenyl)-1-tosyl-1*H*-benzo[*d*]imidazole **(2i)**

^{13}C NMR (100 MHz, CDCl$_3$)

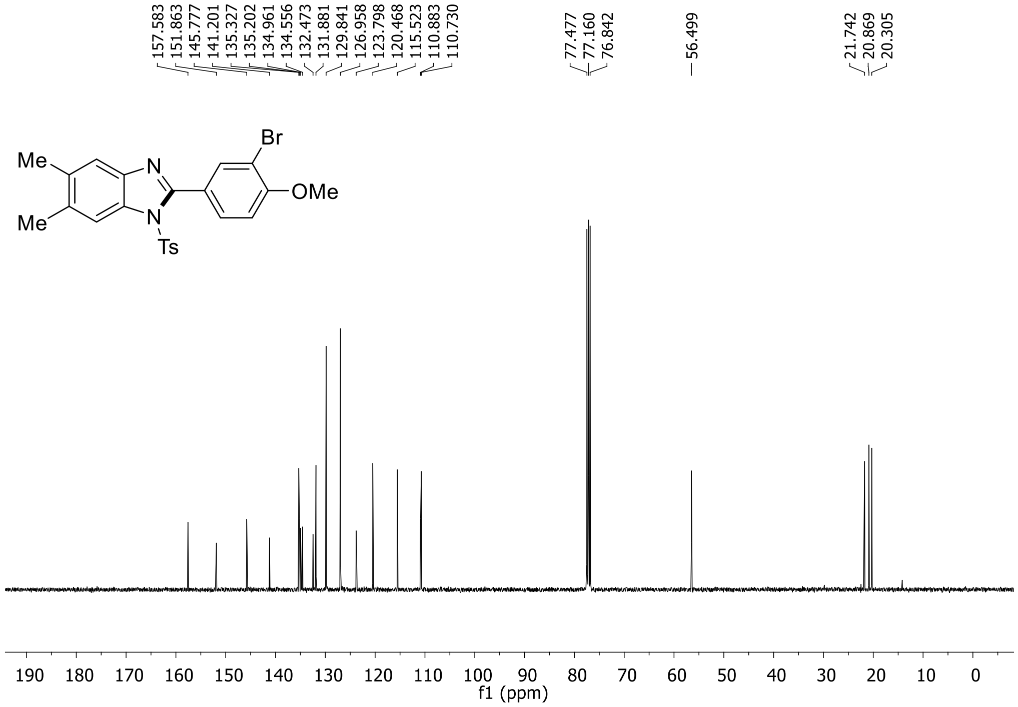

Figure S19. ^{13}C NMR of 2-(3-bromo-4-methoxyphenyl)-1-tosyl-1*H*-benzo[*d*]imidazole **(2i)**

^{1}H NMR (400 MHz, $CDCl_3$)

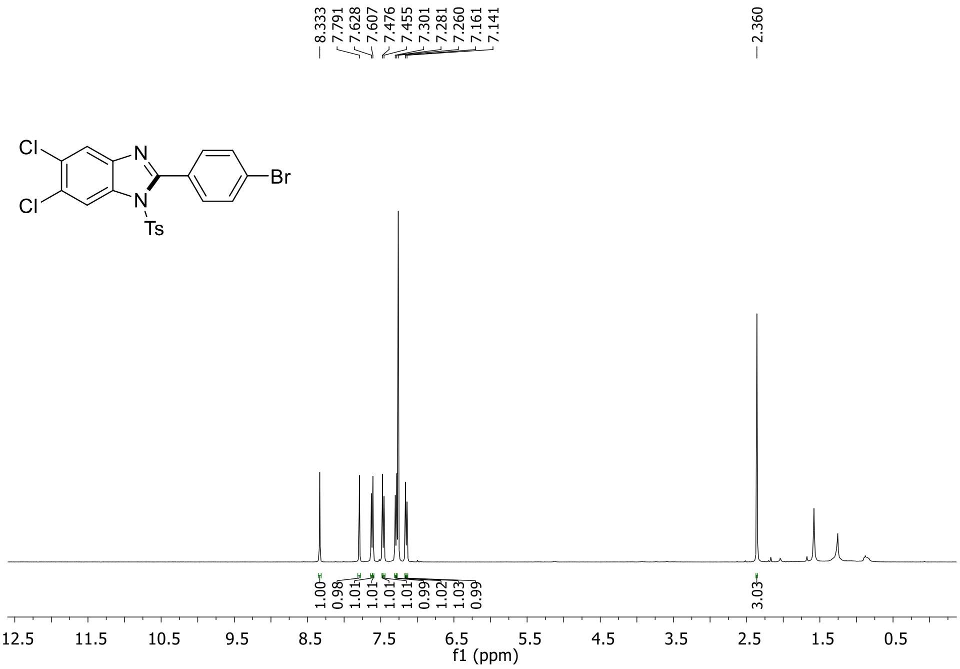

Figure S20. ^{1}H NMR of 2-(4-bromophenyl)-5,6-dichloro-1-tosyl-1*H*-benzo[*d*]imidazole **(2j)**

^{13}C NMR (100 MHz, $CDCl_3$)

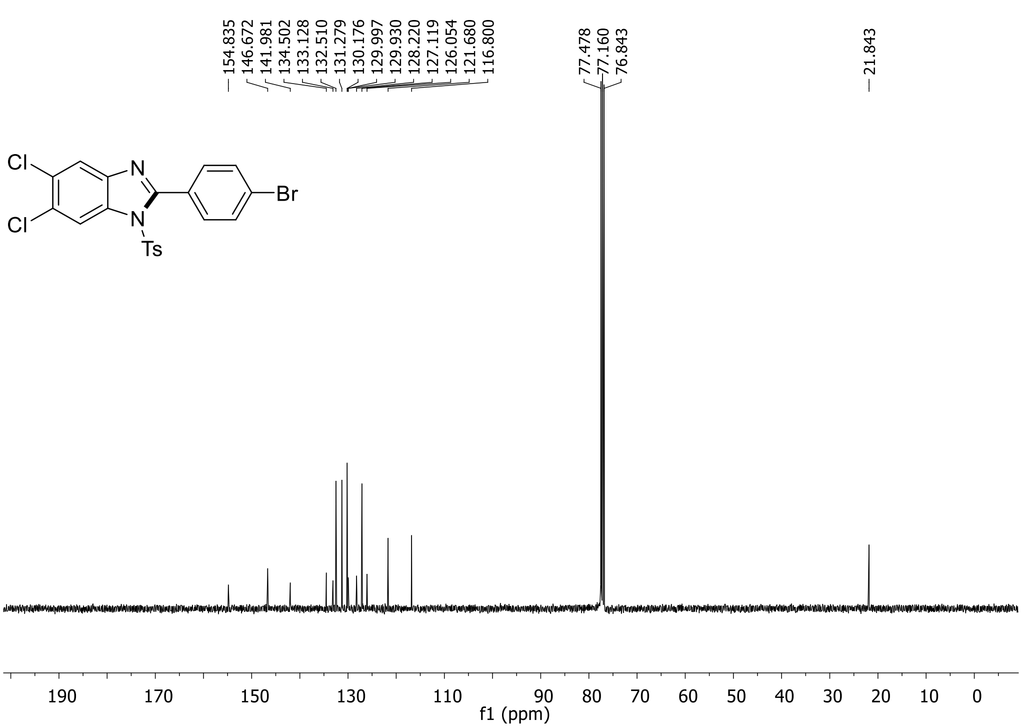

Figure S21. ^{13}C NMR of 2-(4-bromophenyl)-5,6-dichloro-1-tosyl-1*H*-benzo[*d*]imidazole **(2j)**

^{1}H NMR (400 MHz, CDCl$_3$)

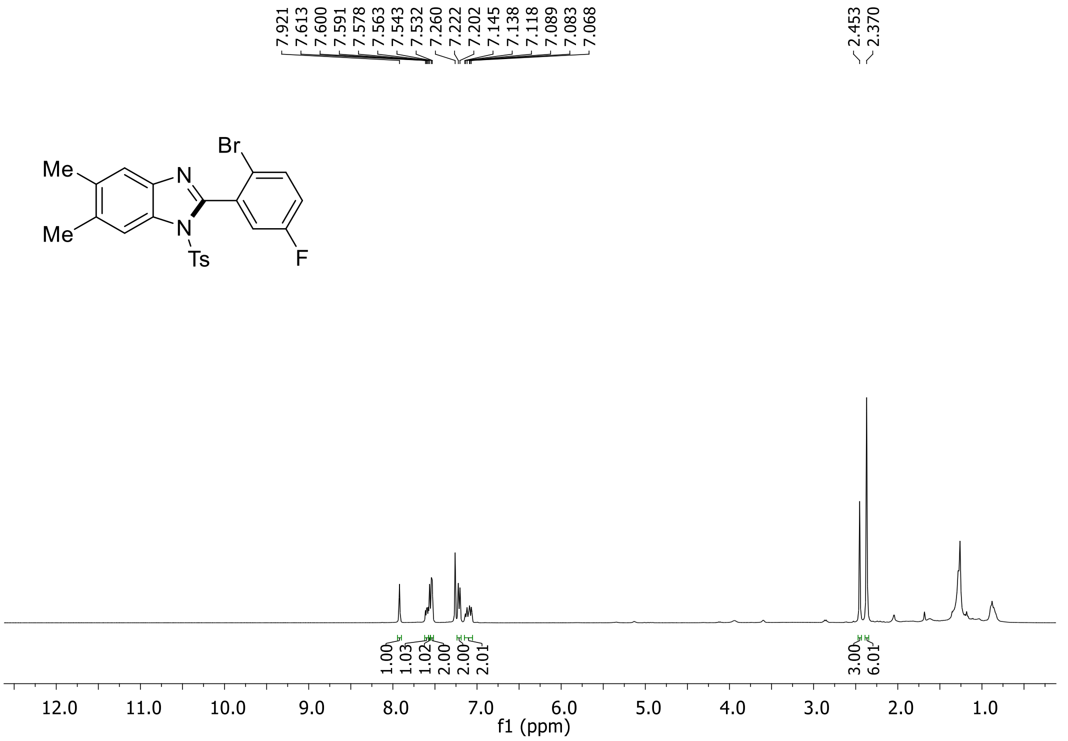

Figure S22. ^{1}H NMR of 2-(2-bromo-5-fluorophenyl)-5,6-dimethyl-1-tosyl-1*H*-benzo[*d*]imidazole **(2k)**

^{13}C NMR (100 MHz, CDCl$_3$)

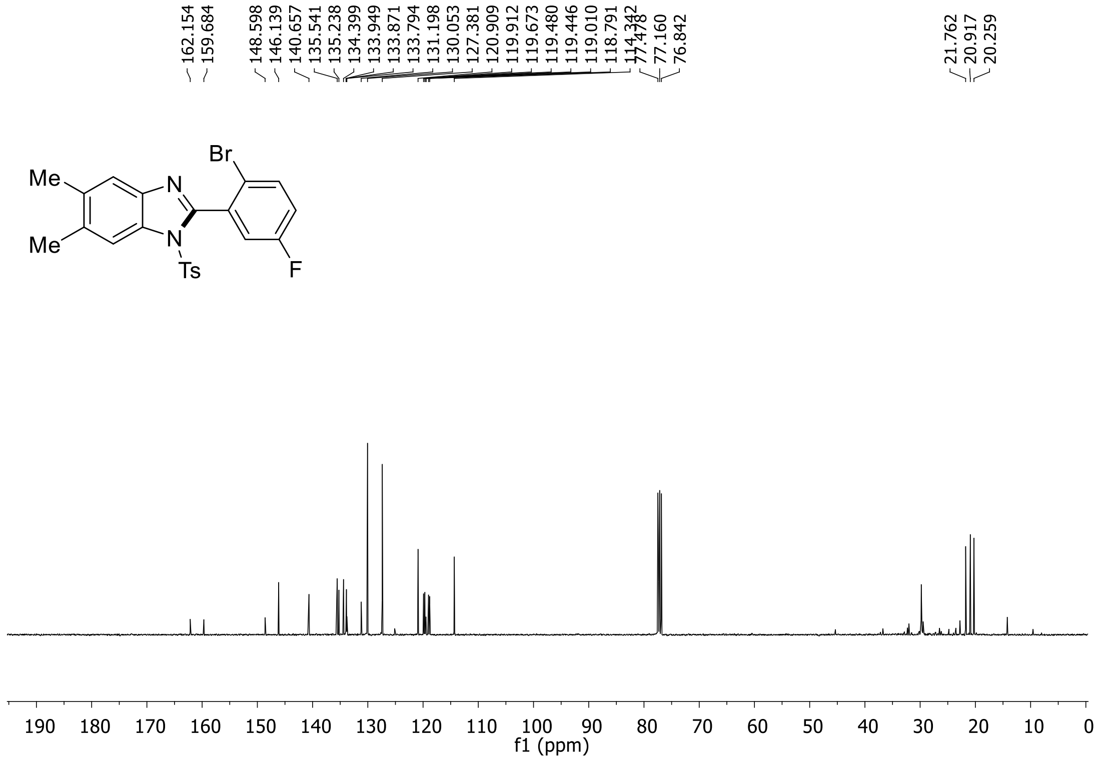

Figure S23. ^{13}C NMR of 2-(2-bromo-5-fluorophenyl)-5,6-dimethyl-1-tosyl-1*H*-benzo[*d*]imidazole **(2k)**

^{1}H NMR (400 MHz, DMSO-d_6)

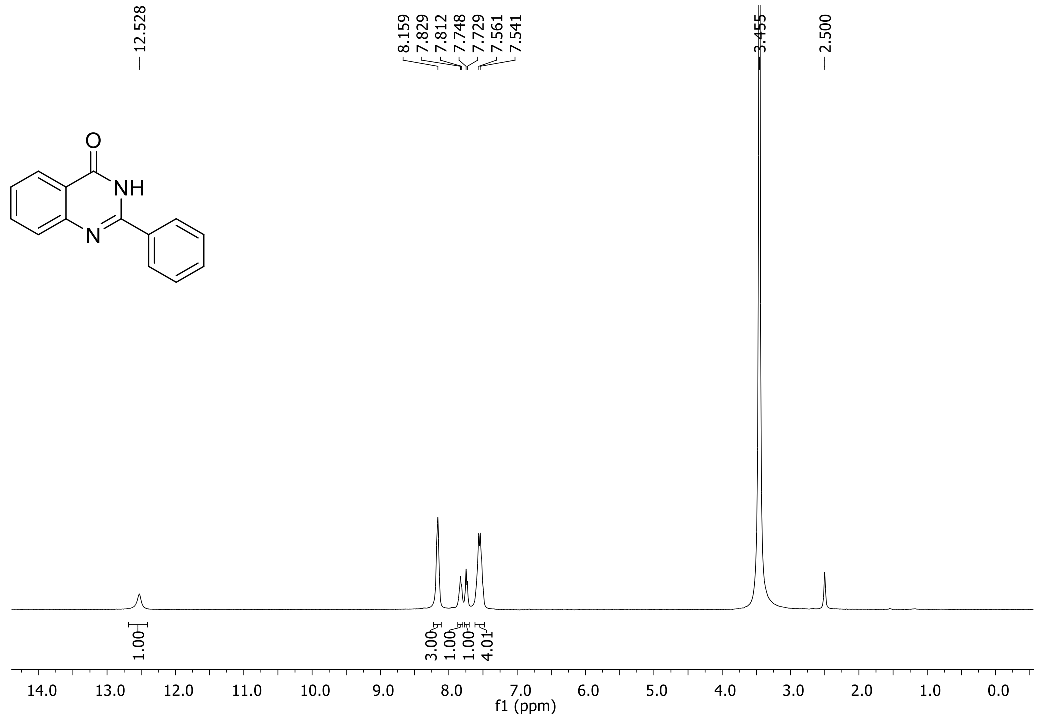

Figure S24. ^{1}H NMR of 2-phenylquinazolin-4(3*H*)-one **(5a)**

^{13}C NMR (100 MHz, DMSO-d_6)

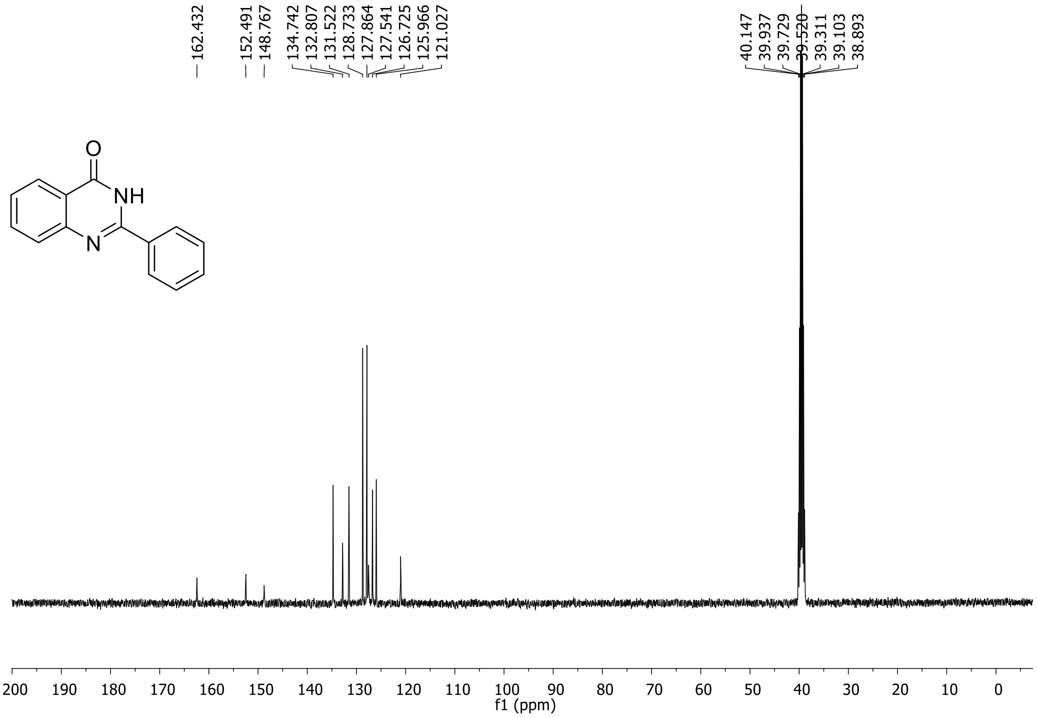

Figure S25. ^{13}C NMR of 2-phenylquinazolin-4(3*H*)-one **(5a)**

^{1}H NMR (400 MHz, DMSO-d$_6$)

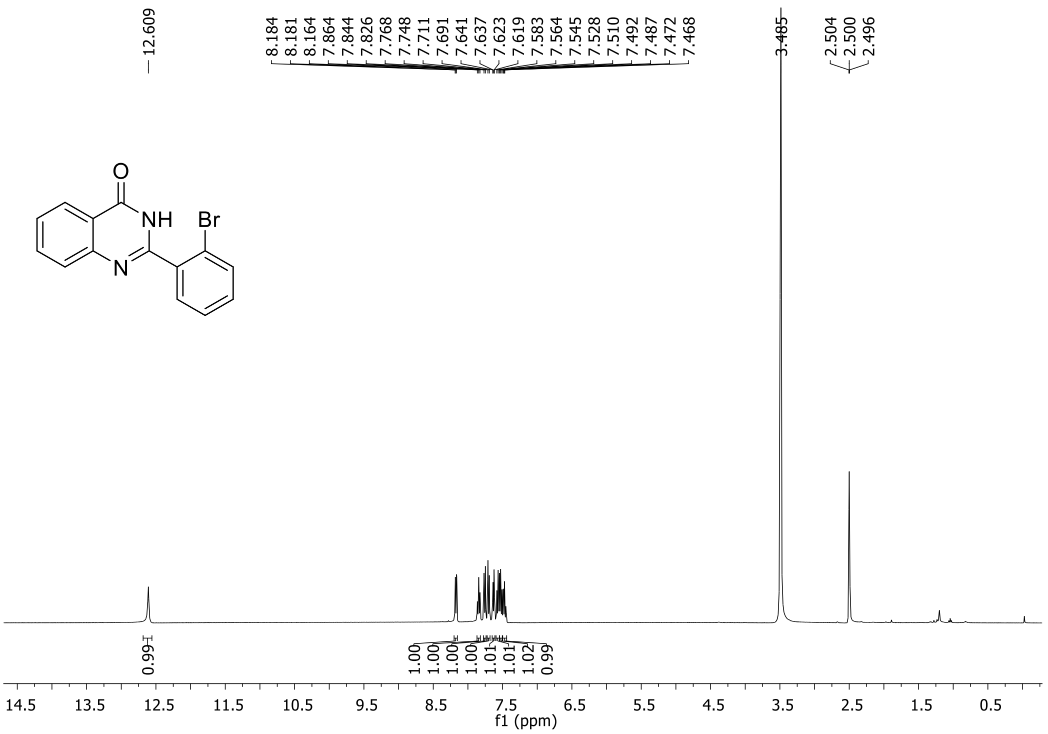

Figure S26. ^{1}H NMR of 2-(2-bromophenyl)quinazolin-4(3*H*)-one **(5b)**

^{13}C NMR (100 MHz, DMSO-d$_6$)

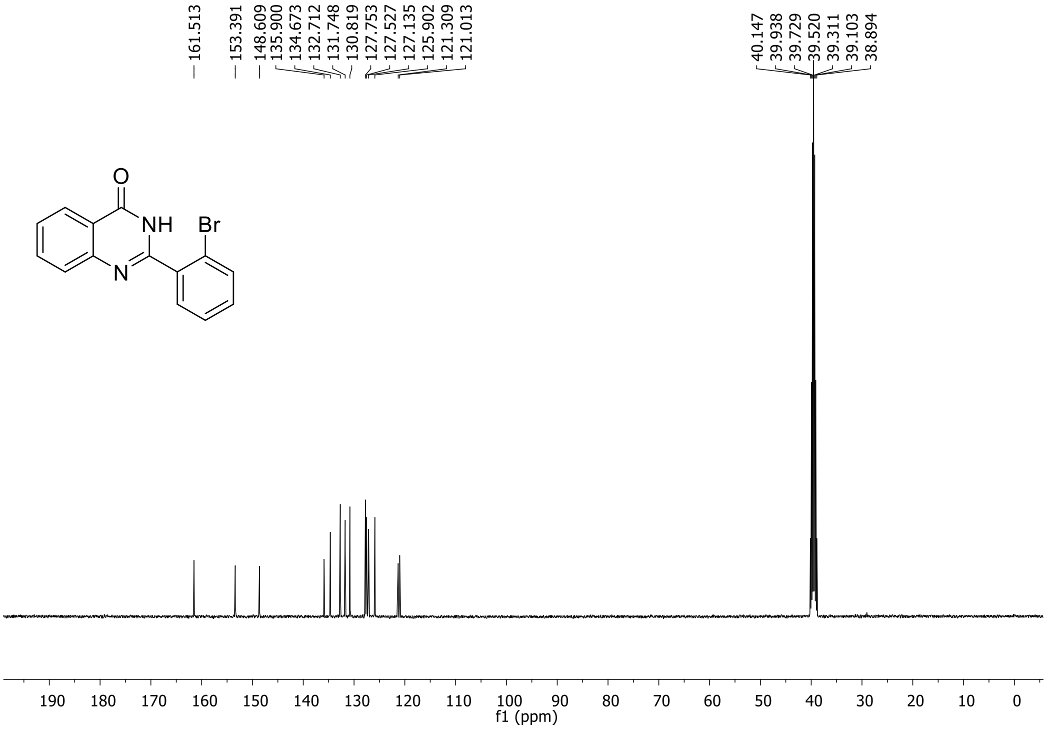

Figure S27. ^{13}C NMR of 2-(2-bromophenyl)quinazolin-4(3*H*)-one **(5b)**

^{1}H NMR (400 MHz, DMSO-d$_6$)

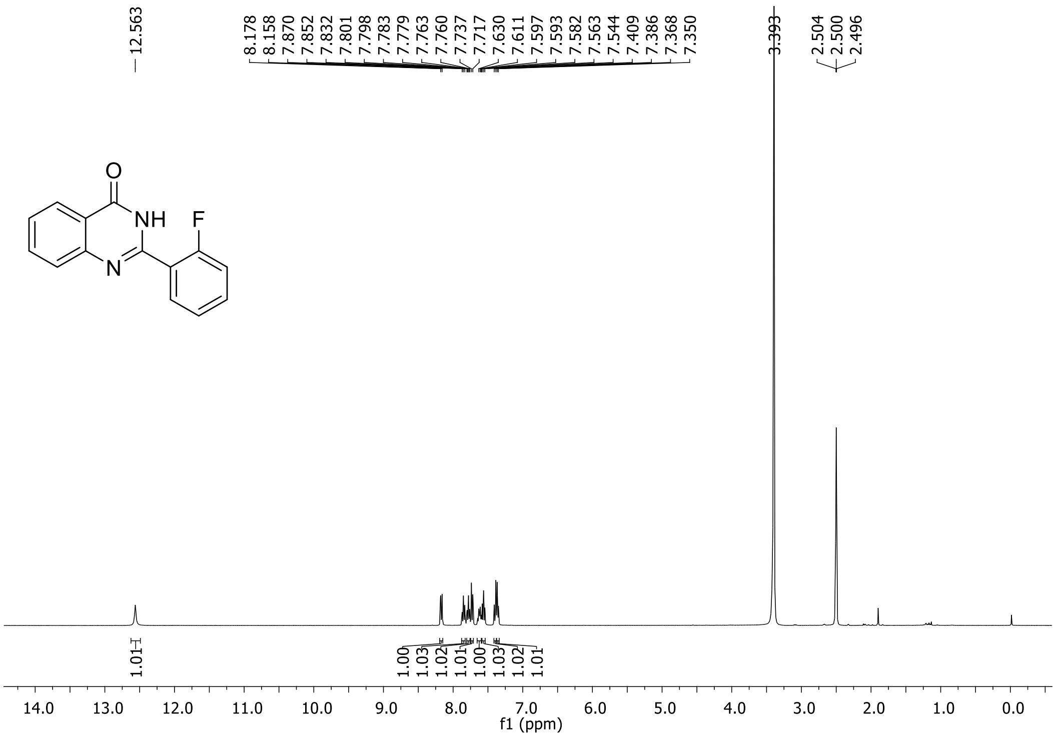

Figure S28. ^{1}H NMR of 2-(2-fluorophenyl)quinazolin-4(3*H*)-one **(5c)**

^{13}C NMR (100 MHz, DMSO-d$_6$)

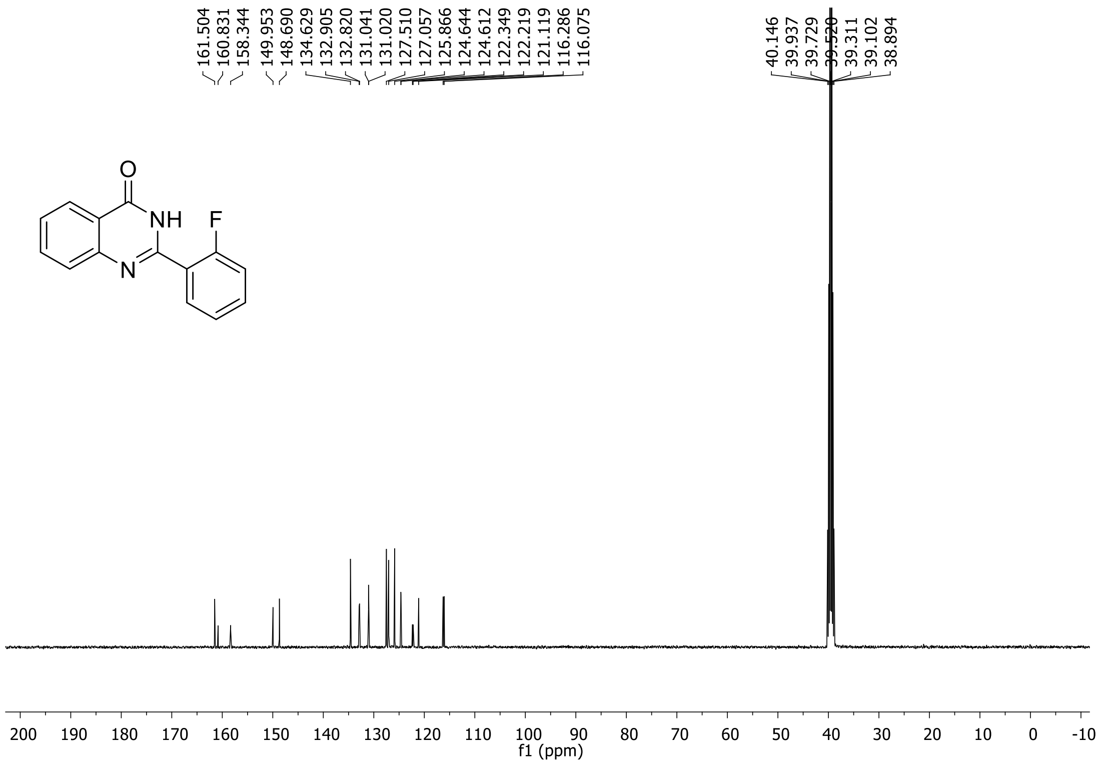

Figure S29. ^{13}C NMR of 2-(2-fluorophenyl)quinazolin-4(3*H*)-one **(5c)**

^{1}H NMR (400 MHz, DMSO-d$_6$)

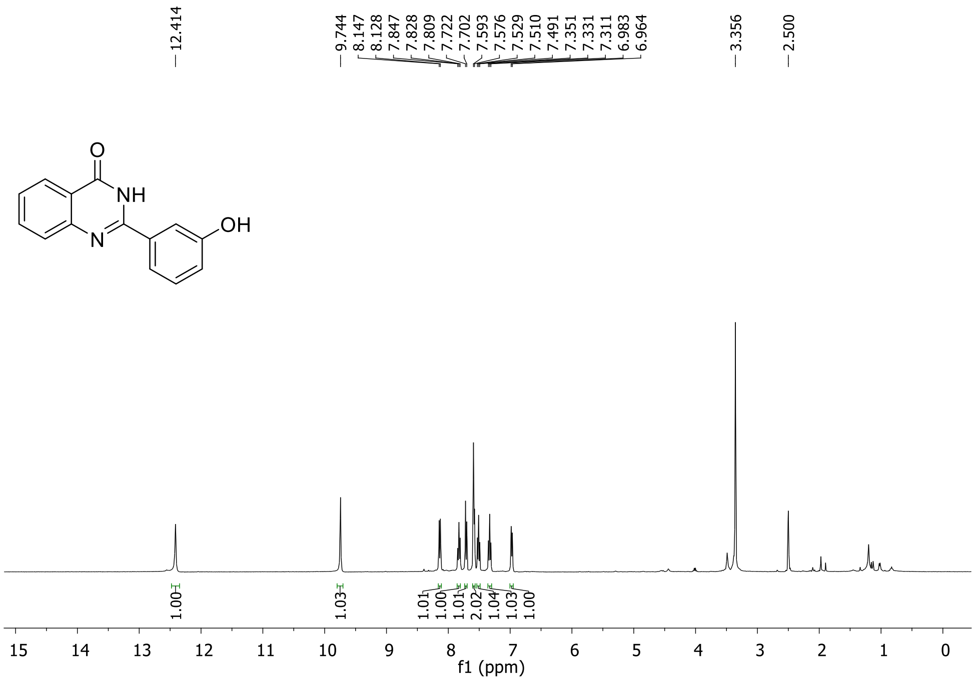

Figure S30. ^{1}H NMR of 2-(3-hydroxyphenyl)quinazolin-4(3*H*)-one **(5d)**

^{13}C NMR (100 MHz, DMSO-d$_6$)

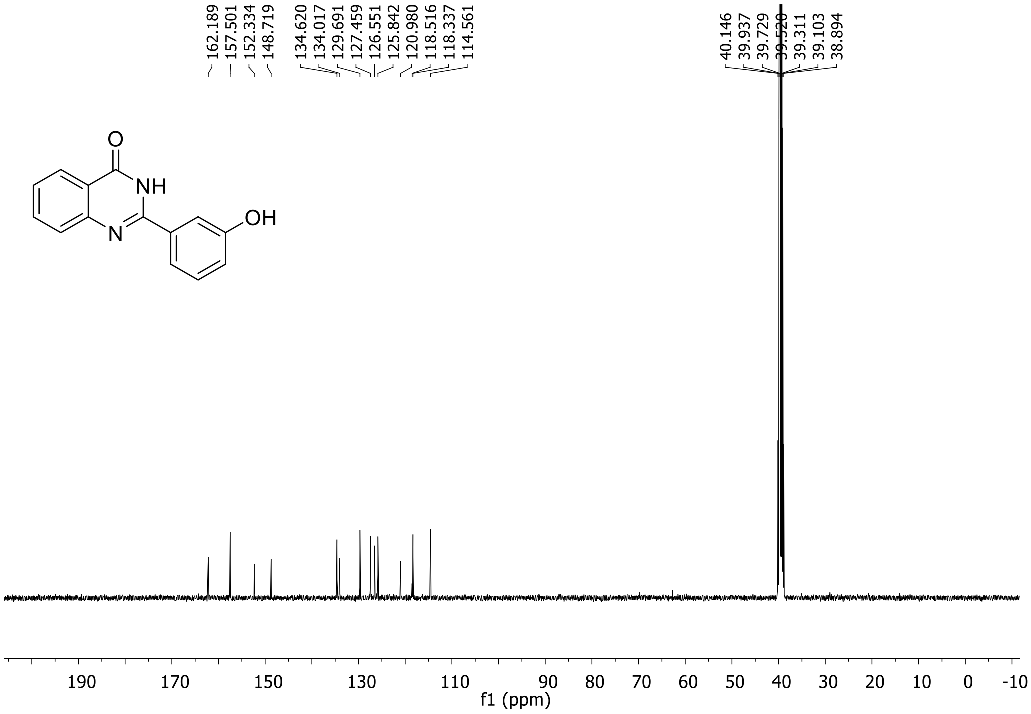

Figure S31. ^{13}C NMR of 2-(3-hydroxyphenyl)quinazolin-4(3*H*)-one **(5d)**

^{1}H NMR (400 MHz, DMSO-d$_6$)

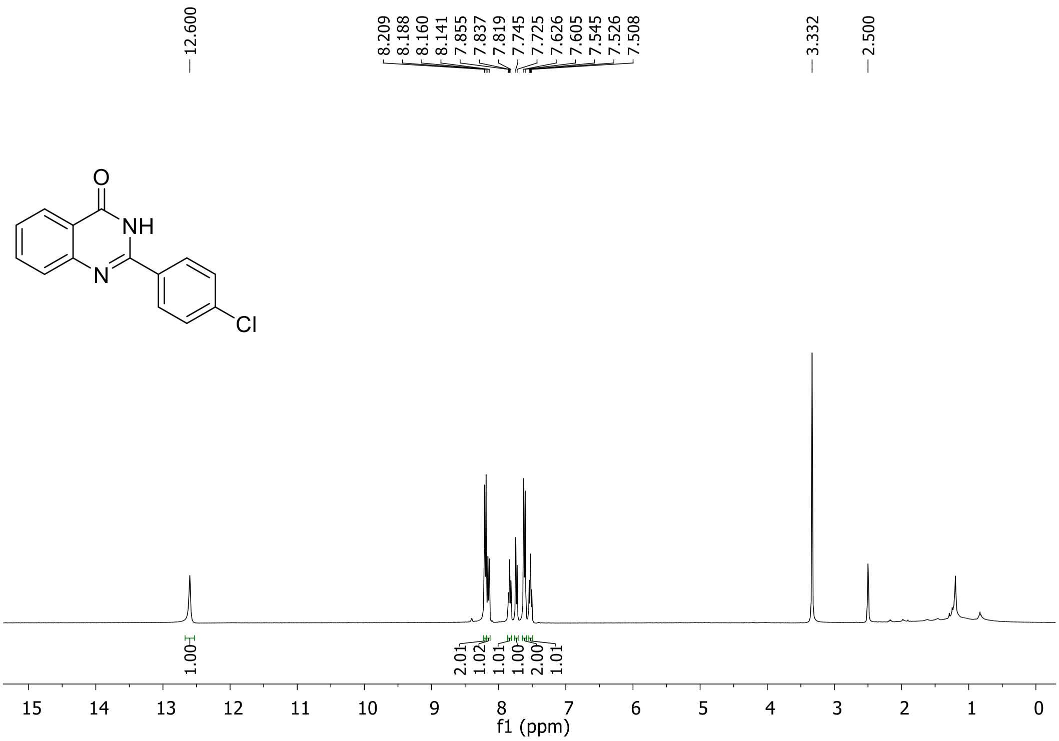

Figure S32. ^{1}H NMR of 2-(4-chlorophenyl)quinazolin-4(3*H*)-one **(5e)**

^{13}C NMR (100 MHz, DMSO-d$_6$)

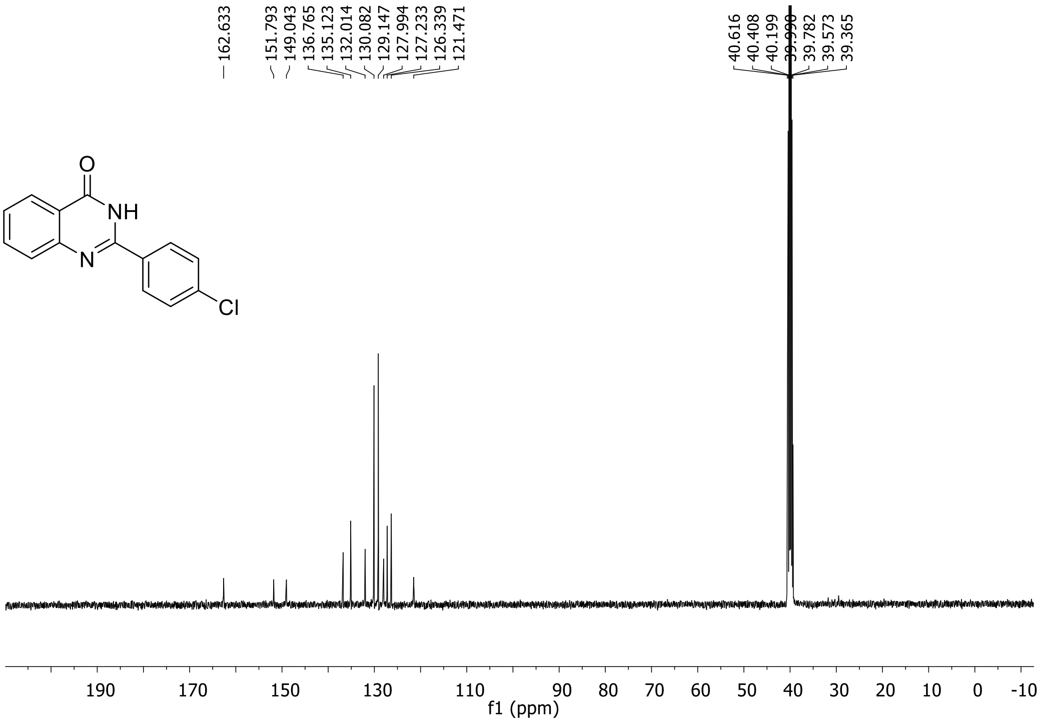

Figure S33. ^{13}C NMR of 2-(4-chlorophenyl)quinazolin-4(3*H*)-one **(5e)**

^{1}H NMR (400 MHz, DMSO-d$_6$)

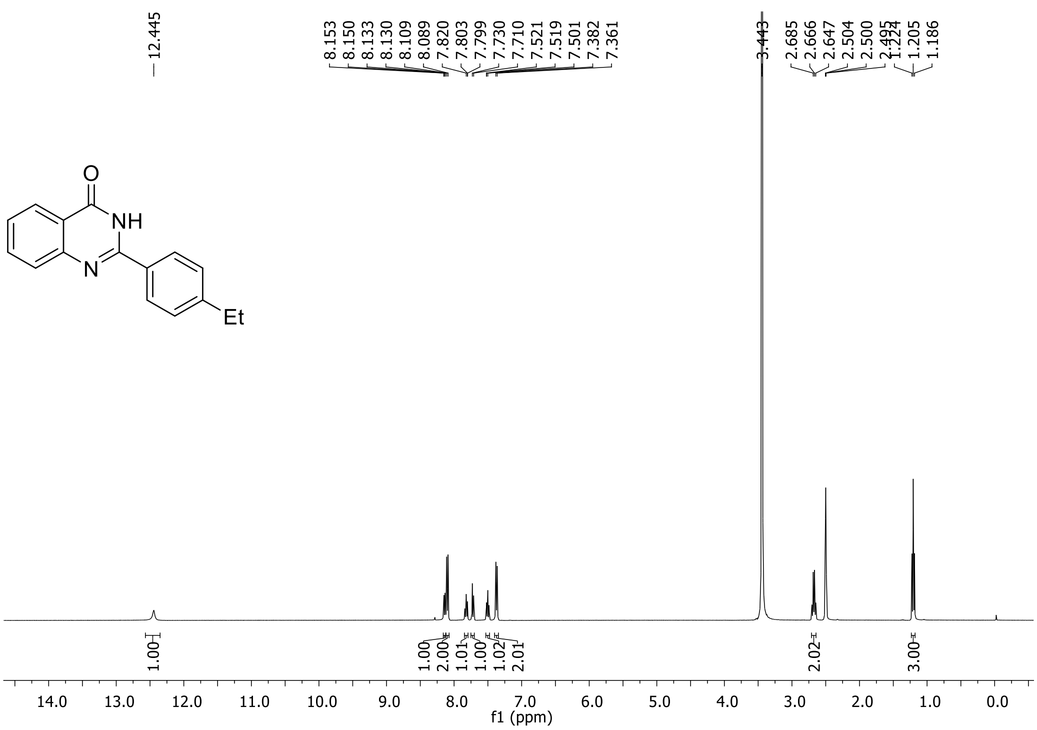

Figure S34. ^{1}H NMR of 2-(4-ethylphenyl)quinazolin-4(3*H*)-one **(5f)**

^{13}C NMR (100 MHz, DMSO-d$_6$)

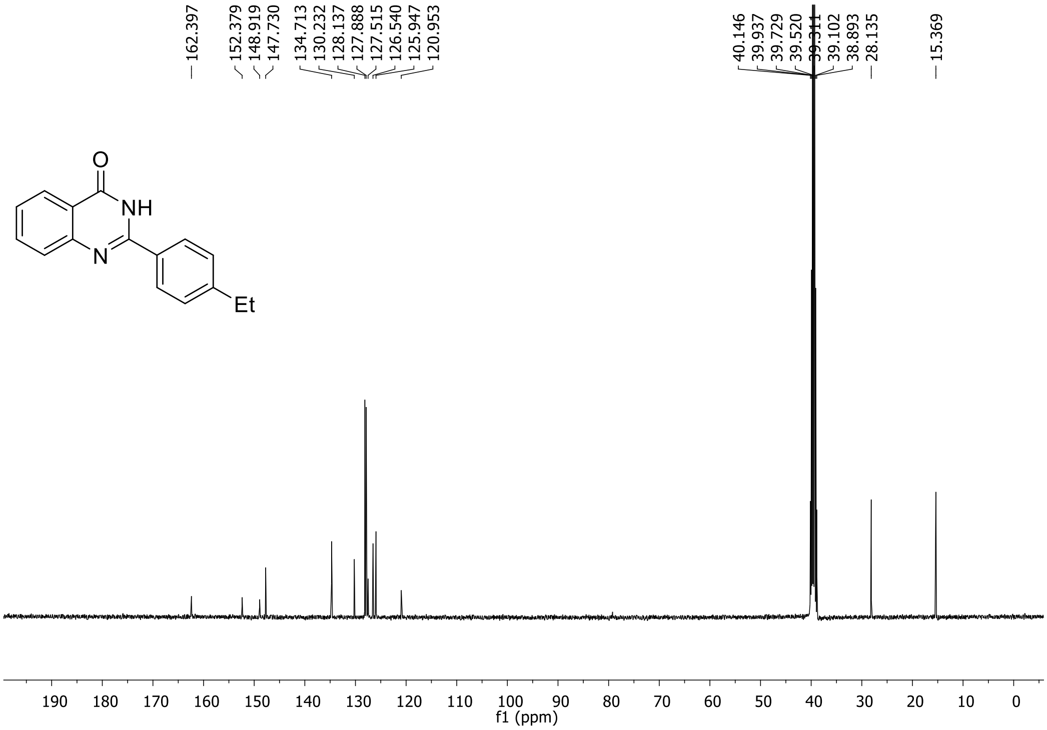

Figure S35. ^{13}C NMR of 2-(4-ethylphenyl)quinazolin-4(3*H*)-one **(5f)**

^{1}H NMR (400 MHz, CDCl$_3$)

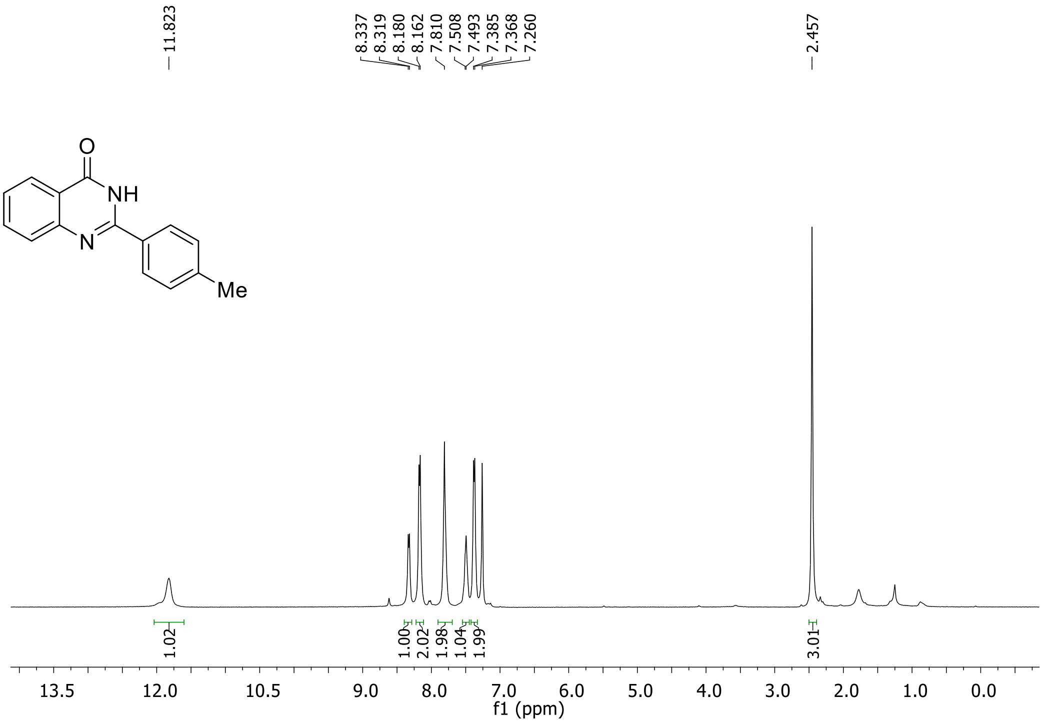

Figure S36. ^{1}H NMR of 2-(*p*-tolyl)quinazolin-4(3*H*)-one **(5g)**

^{13}C NMR (100 MHz, CDCl$_3$)

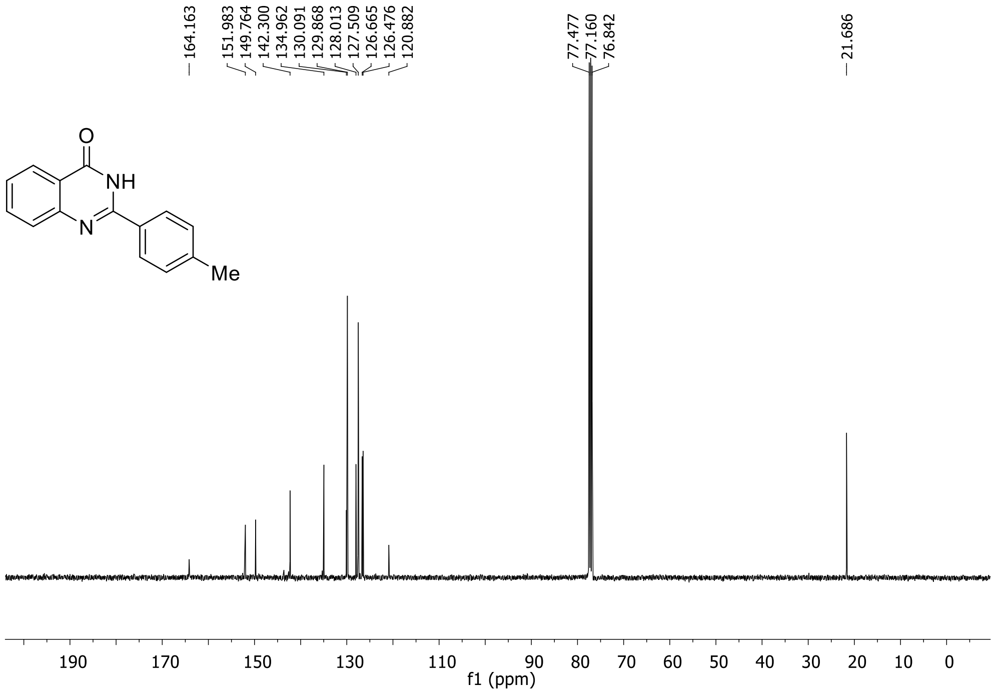

Figure S37. ^{13}C NMR of 2-(*p*-tolyl)quinazolin-4(3*H*)-one **(5g)**

^{1}H NMR (400 MHz, $CDCl_3$)

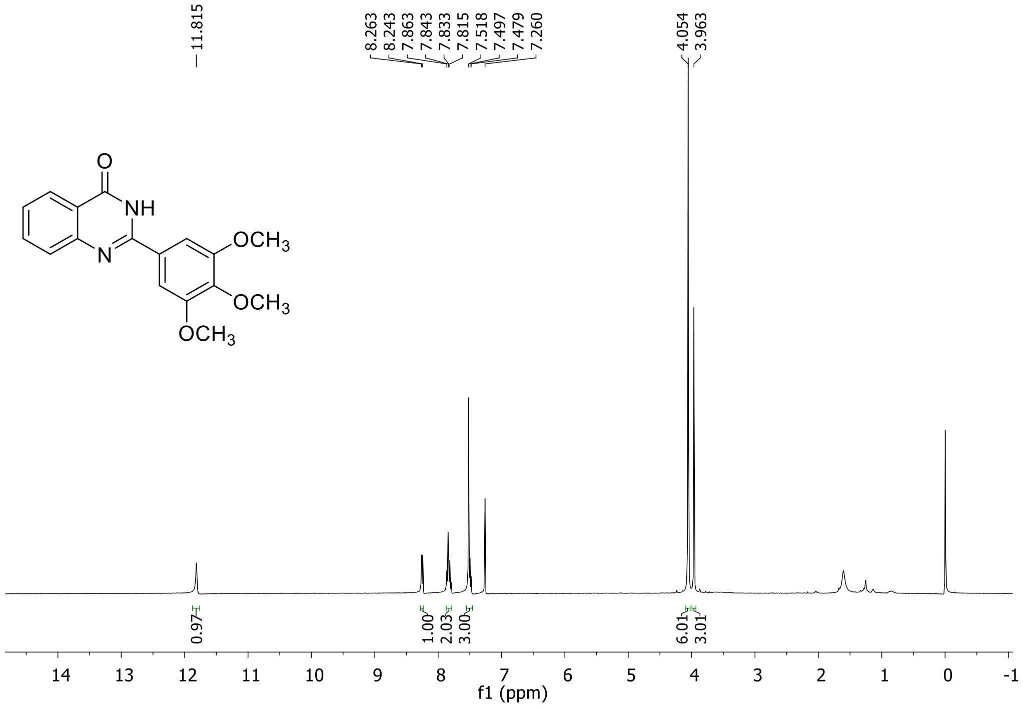

Figure S38. ^{1}H NMR of 2-(3,4,5-trimethoxyphenyl)quinazolin-4(3*H*)-one **(5h)**

^{13}C NMR (100 MHz, $CDCl_3$)

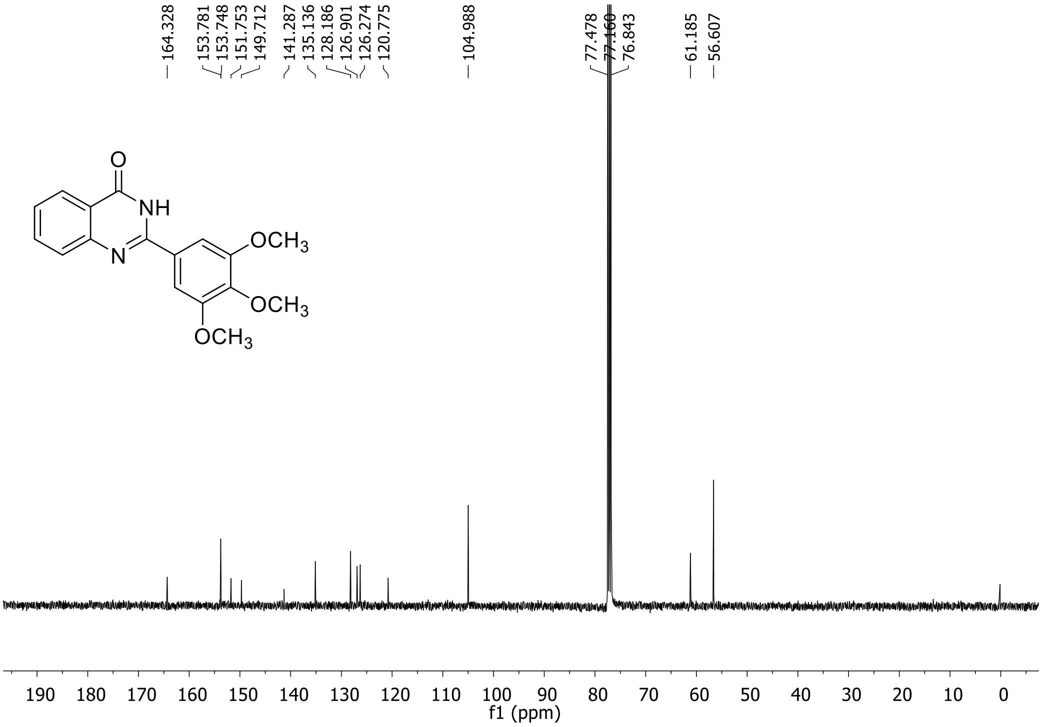

Figure S39. ^{13}C NMR of 2-(3,4,5-trimethoxyphenyl)quinazolin-4(3*H*)-one **(5h)**

^{1}H NMR (400 MHz, DMSO-d$_6$)

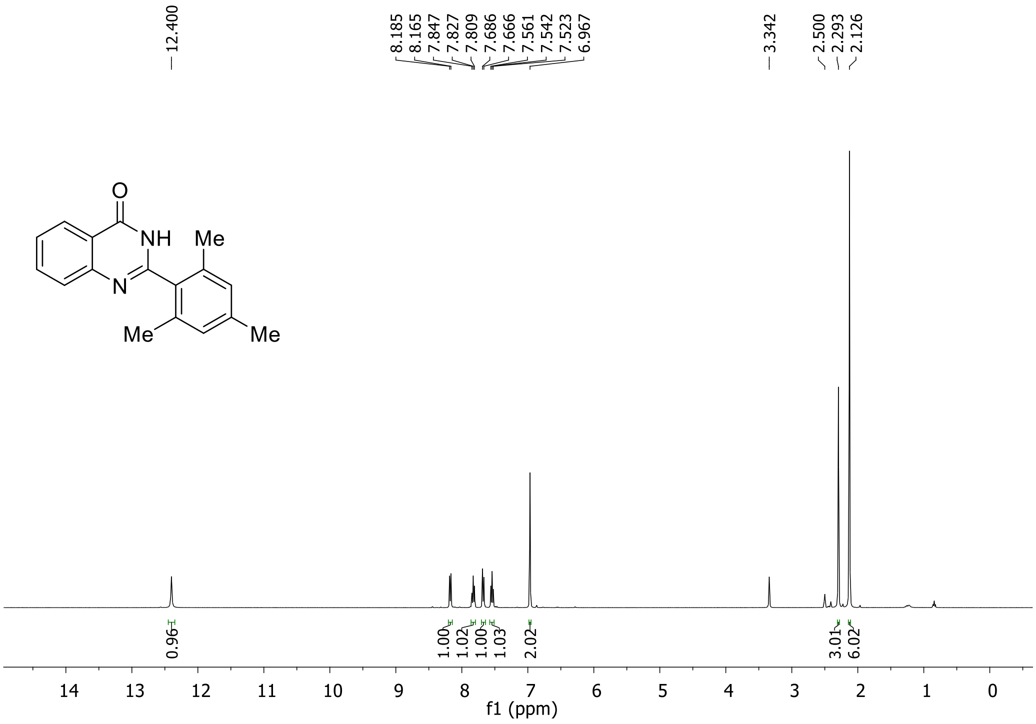

Figure S40. ^{1}H NMR of 2-mesitylquinazolin-4(3*H*)-one **(5i)**

^{13}C NMR (100 MHz, DMSO-d$_6$)

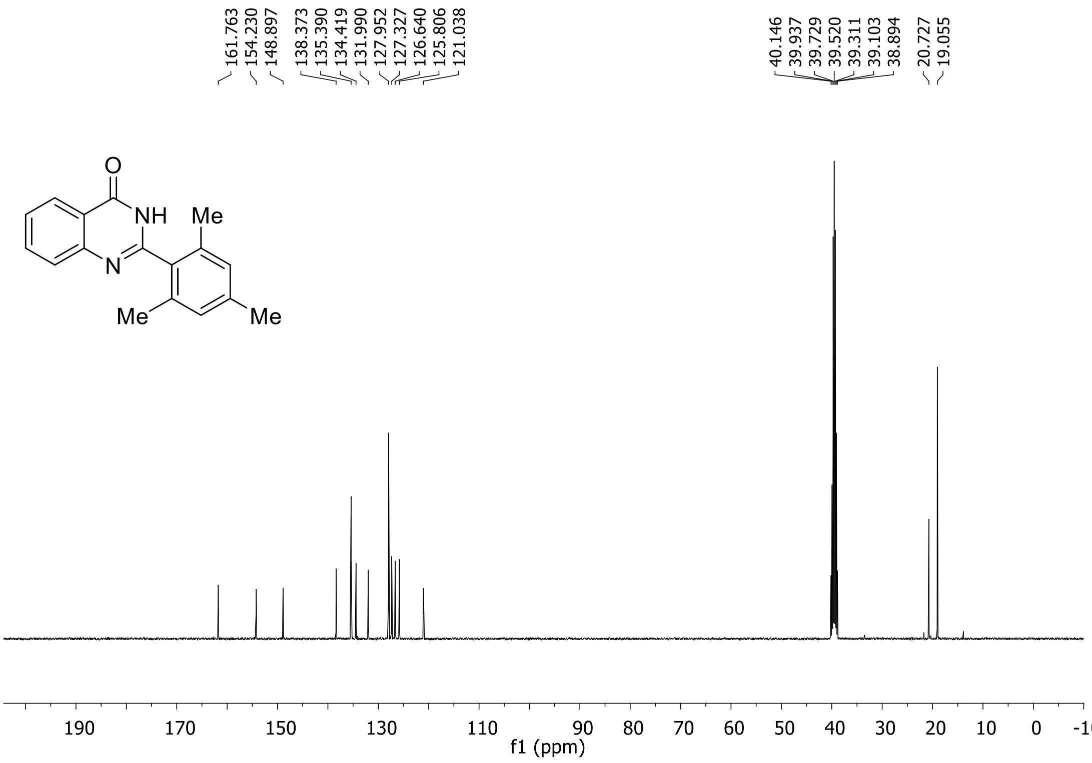

Figure S41. ^{13}C NMR of 2-mesitylquinazolin-4(3*H*)-one **(5i)**

^{1}H NMR (400 MHz, $CDCl_3$)

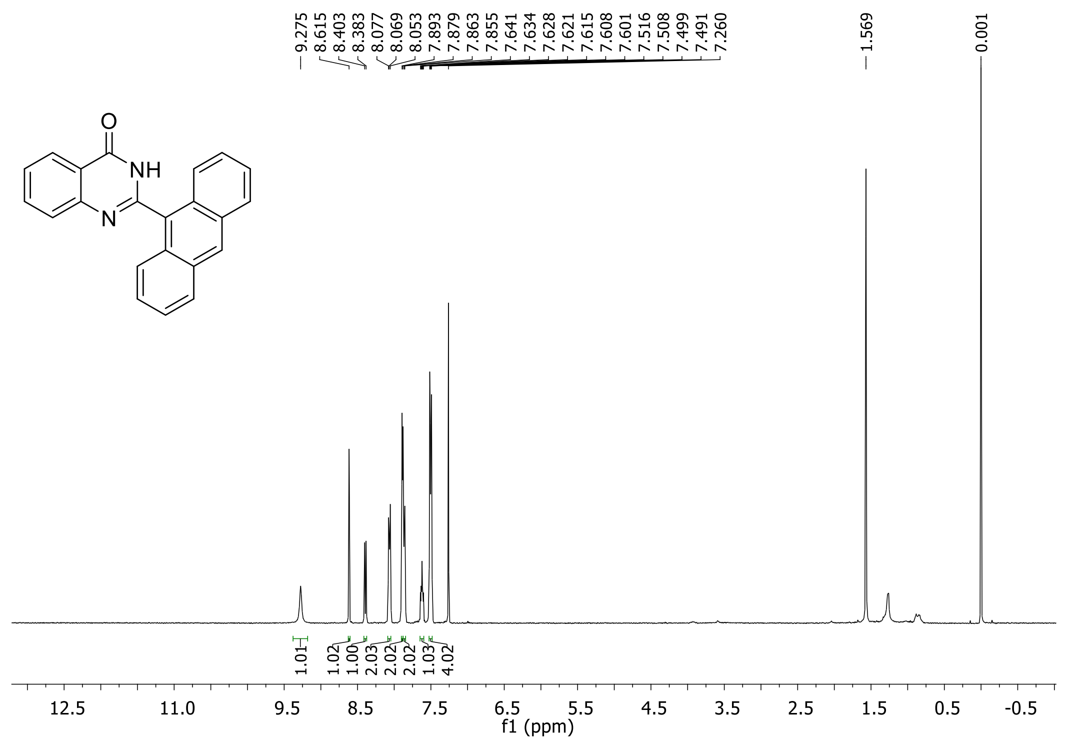

Figure S42. ^{1}H NMR of 2-(anthracen-9-yl)quinazolin-4(3*H*)-one **(5j)**

^{13}C NMR (100 MHz, $CDCl_3$)

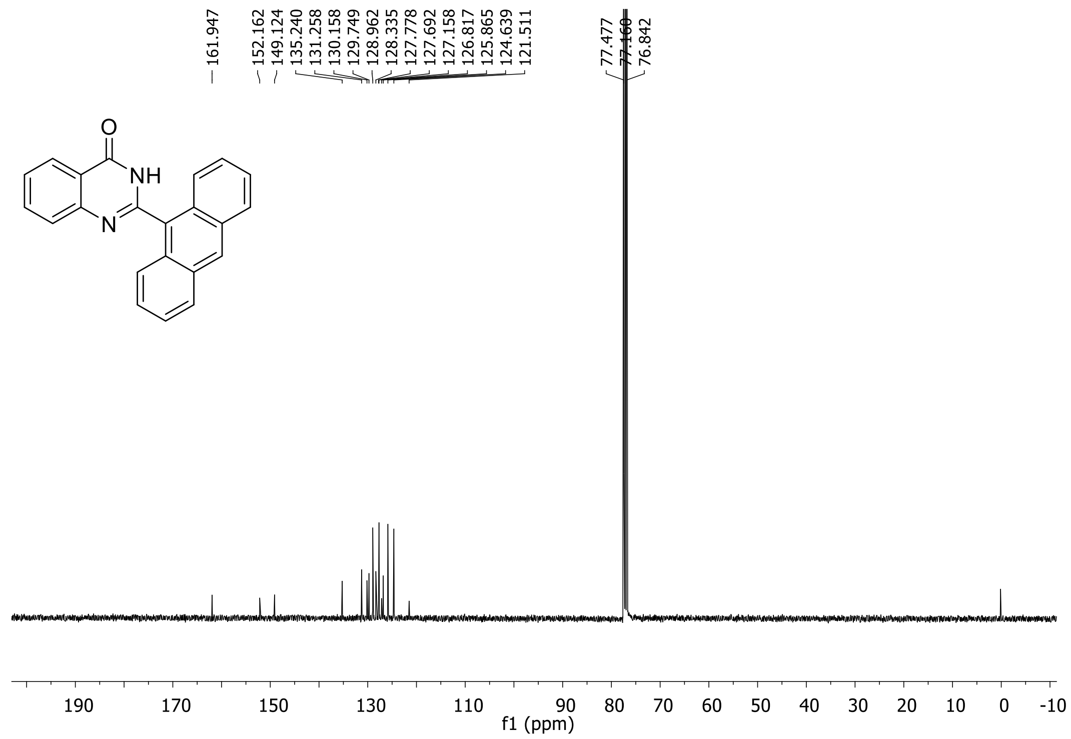

Figure S43. ^{13}C NMR of 2-(anthracen-9-yl)quinazolin-4(3*H*)-one **(5j)**

^{1}H NMR (400 MHz, DMSO-d$_6$)

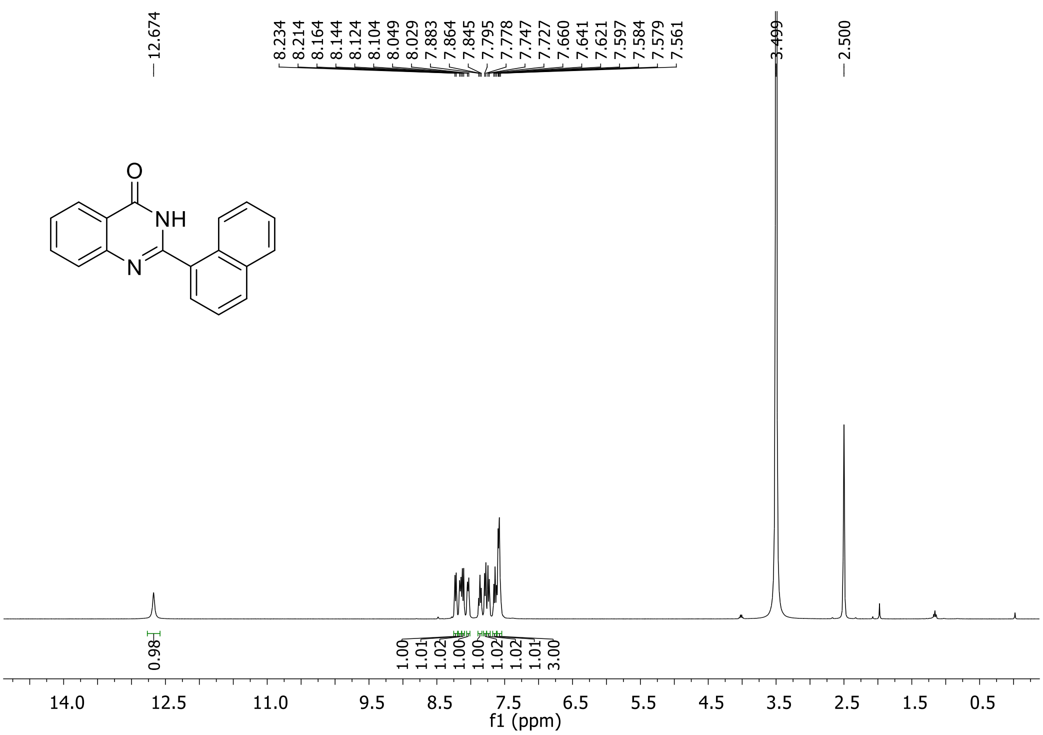

Figure S44. ^{1}H NMR of 2-(naphthalen-1-yl)quinazolin-4(3*H*)-one **(5k)**

^{13}C NMR (100 MHz, DMSO-d$_6$)

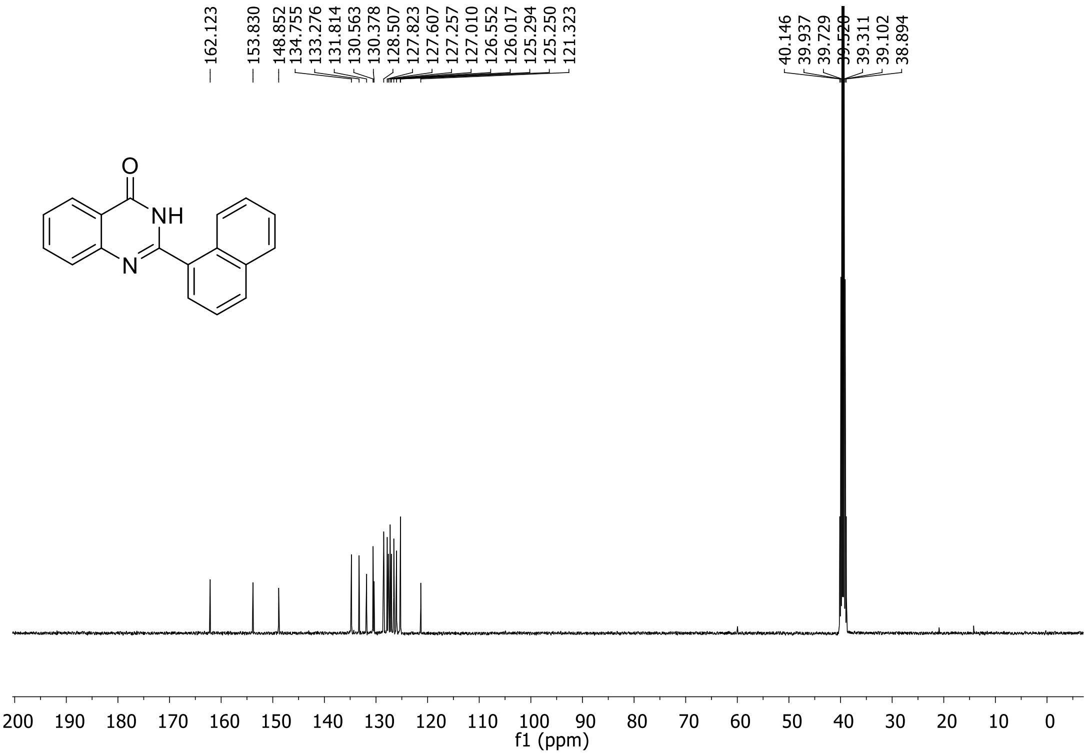

Figure S45. ^{13}C NMR of 2-(naphthalen-1-yl)quinazolin-4(3*H*)-one **(5k)**

^{1}H NMR (400 MHz, $CDCl_3$)

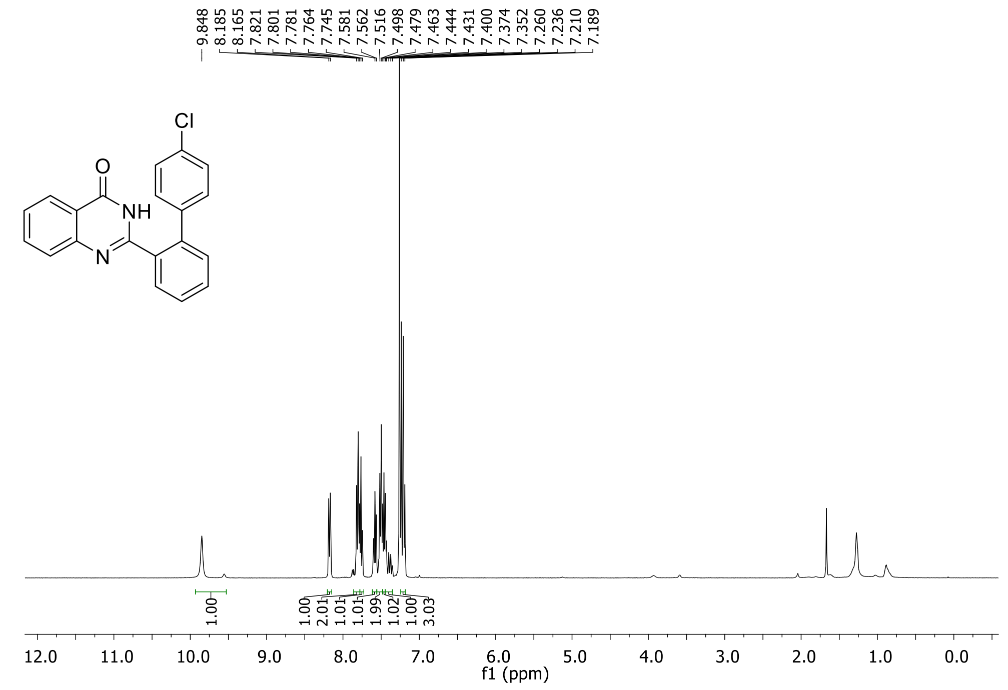

Figure S46. ^{1}H NMR of 2-(4'-chloro-[1,1'-biphenyl]-2-yl)quinazolin-4(3*H*)-one **(5l)**

^{13}C NMR (100 MHz, $CDCl_3$)

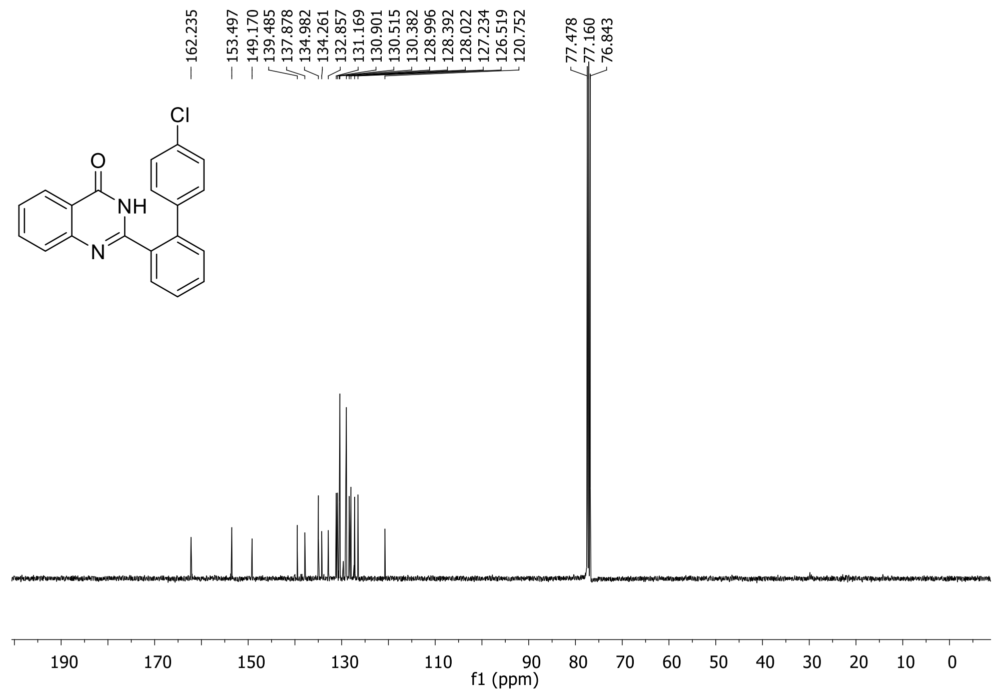

Figure S47. ^{13}C NMR of 2-(4'-chloro-[1,1'-biphenyl]-2-yl)quinazolin-4(3*H*)-one **(5l)**

^{1}H NMR (400 MHz, DMSO-d_6)

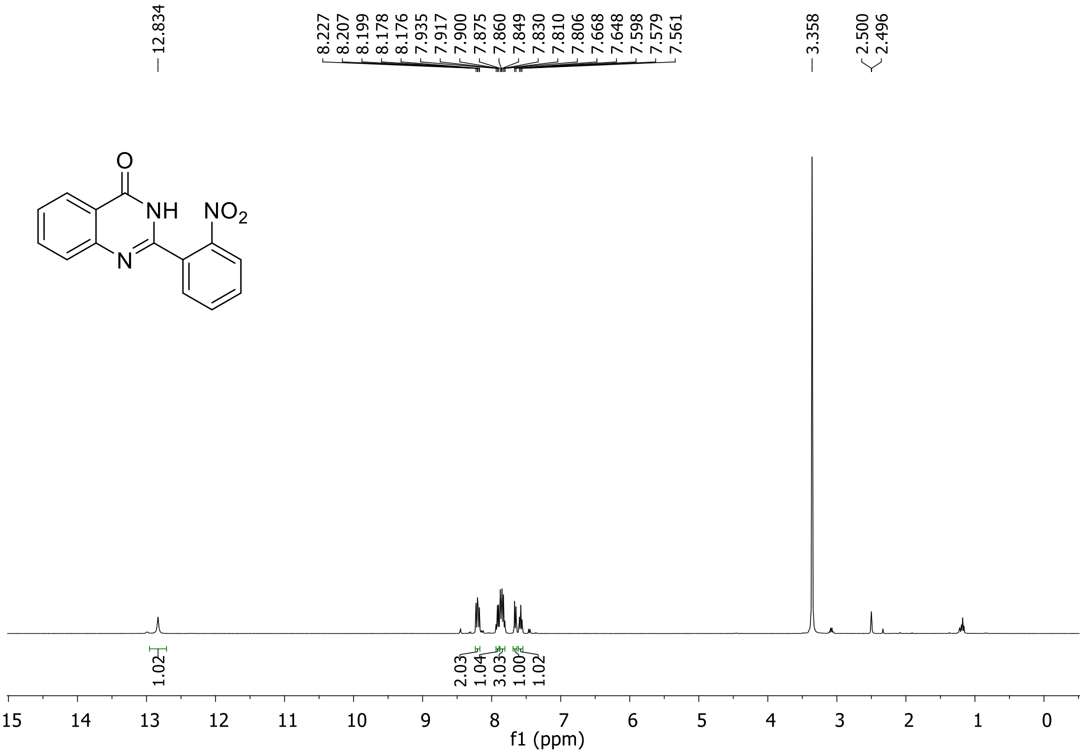

Figure S48. ^{1}H NMR of 2-(2-nitrophenyl)quinazolin-4(3*H*)-one **(5m)**

^{13}C NMR (100 MHz, DMSO-d_6)

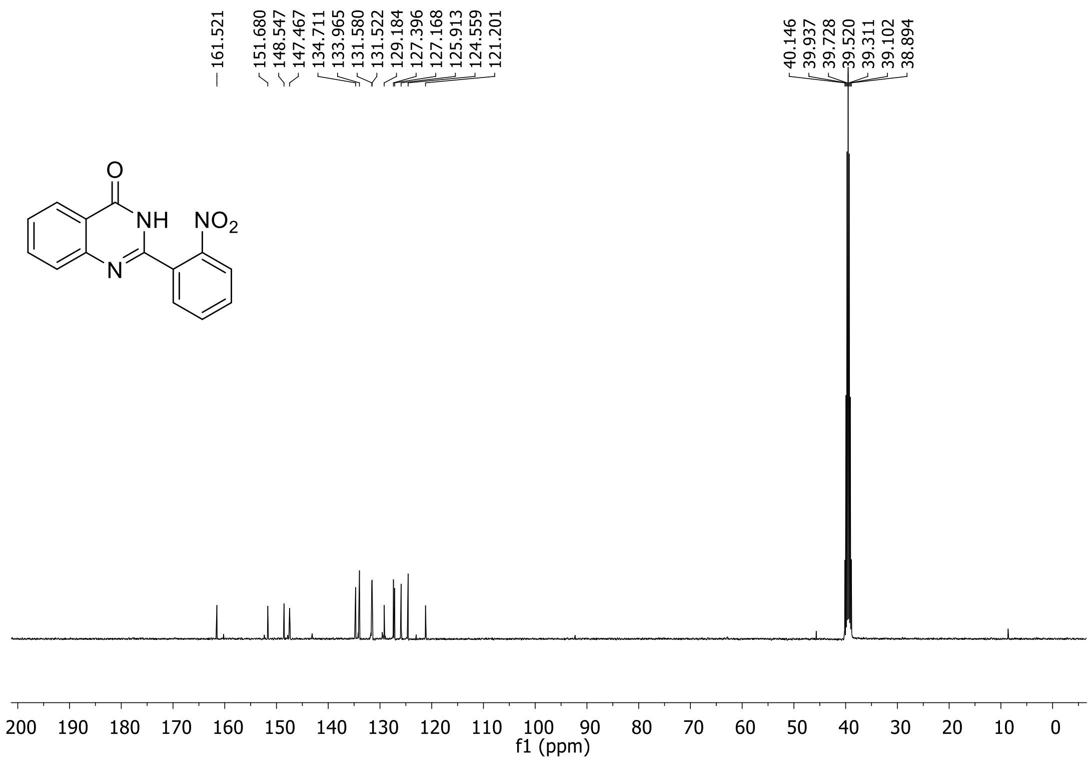

Figure S49. ^{13}C NMR of 2-(2-nitrophenyl)quinazolin-4(3*H*)-one **(5m)**

^{1}H NMR (400 MHz, $CDCl_3$)

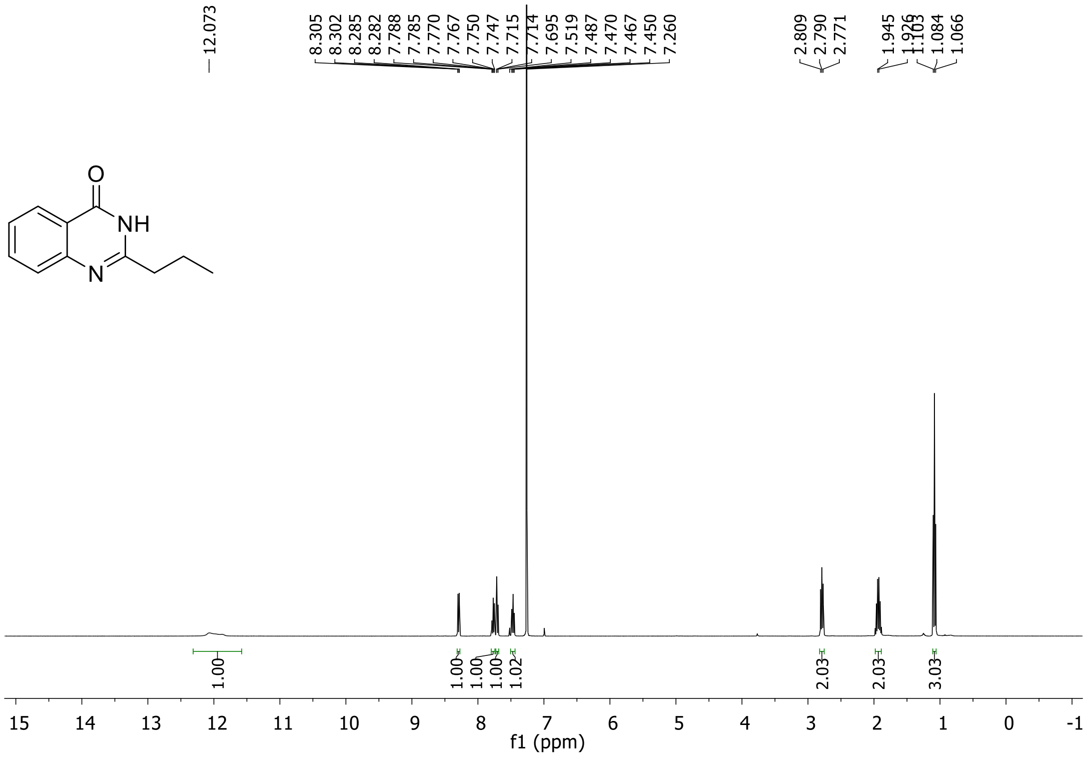

Figure S50. ^{1}H NMR of 2-propylquinazolin-4(3*H*)-one **(5n)**

^{13}C NMR (100 MHz, $CDCl_3$)

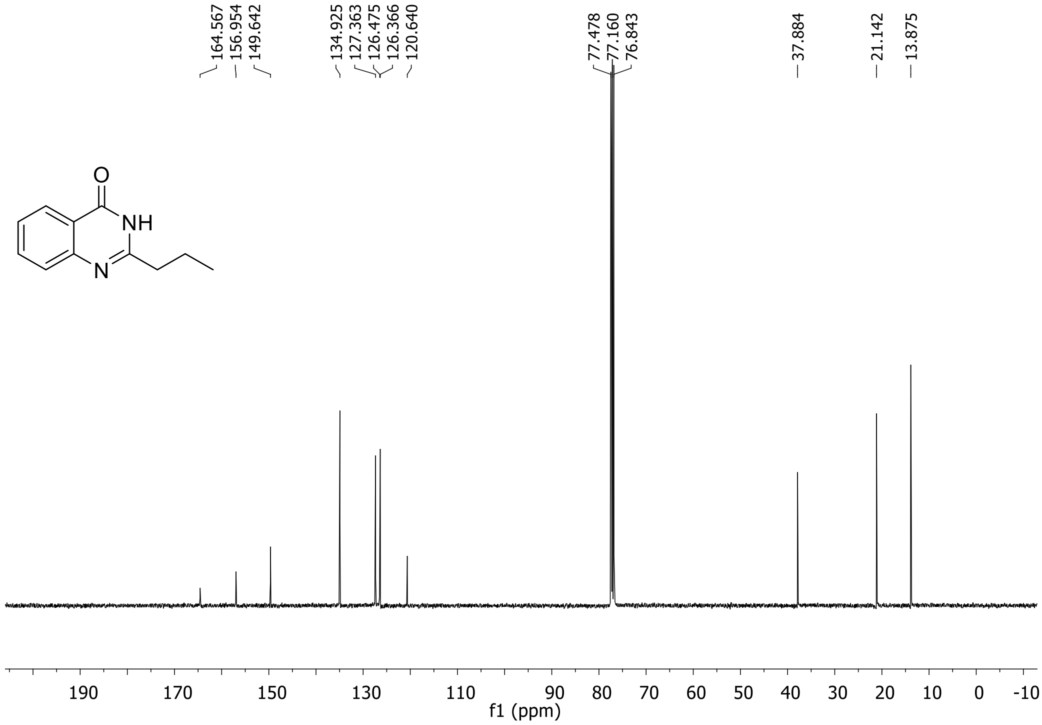

Figure S51. ^{13}C NMR of 2-propylquinazolin-4(3*H*)-one **(5n)**

^{1}H NMR (400 MHz, $CDCl_3$)

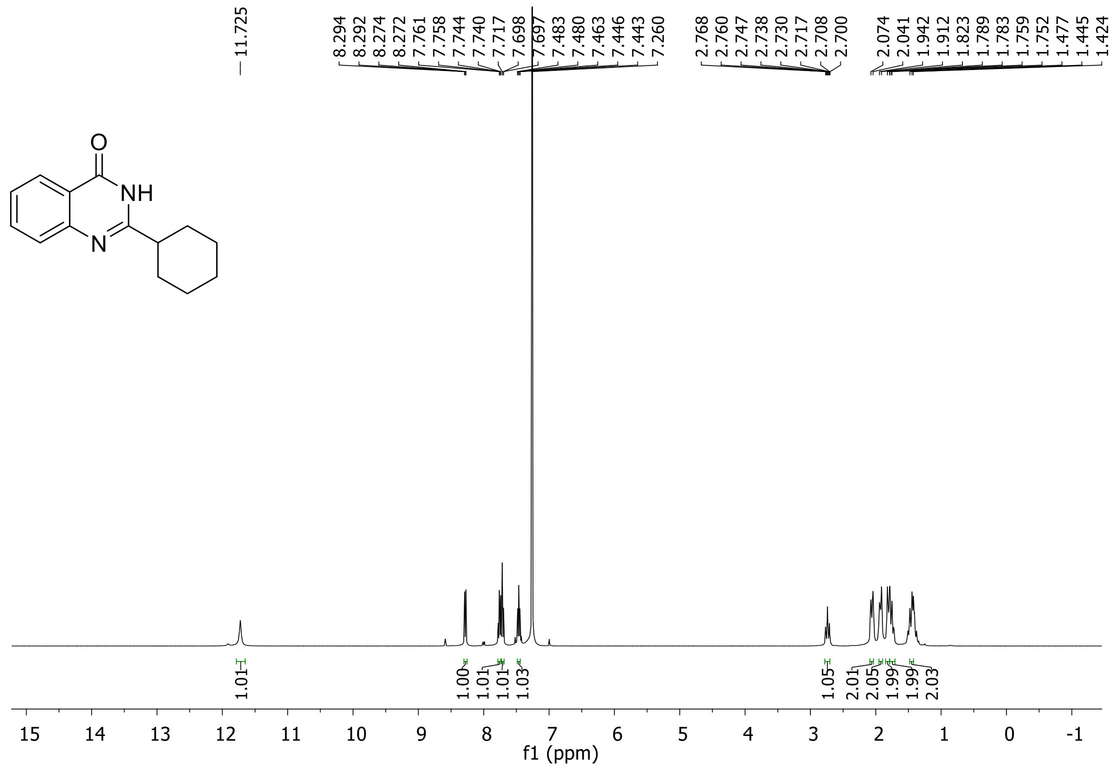

Figure S52. ^{1}H NMR of 2-cyclohexylquinazolin-4(3*H*)-one **(5o)**

^{13}C NMR (100 MHz, $CDCl_3$)

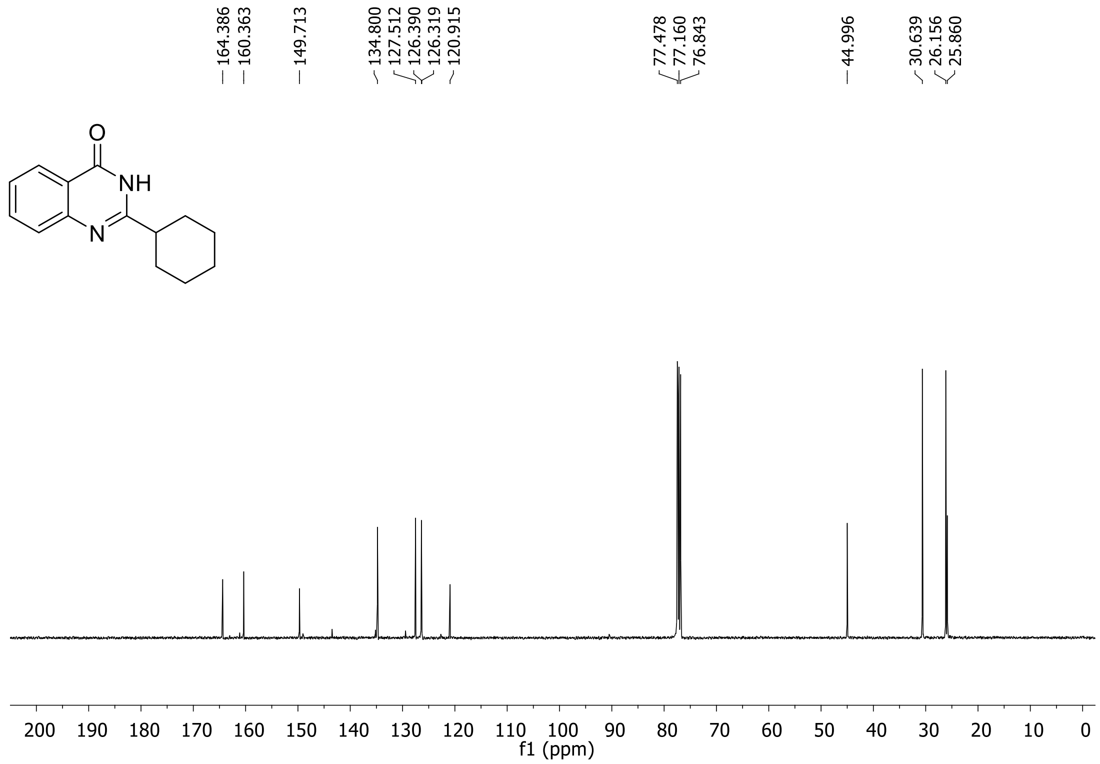

Figure S53. ^{13}C NMR of 2-cyclohexylquinazolin-4(3*H*)-one **(5o)**

^{1}H NMR (700 MHz, $CDCl_3$ + TFA-D)

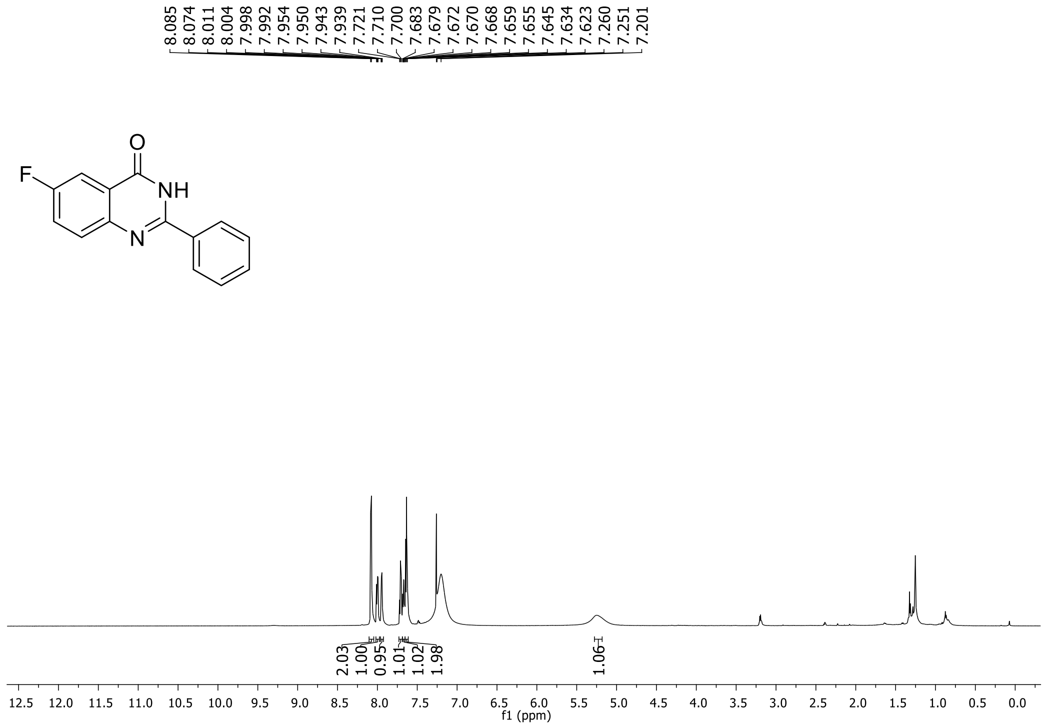

Figure S54. ^{1}H NMR of 6-fluoro-2-phenylquinazolin-4(3*H*)-one **(6a)**

^{13}C NMR (175 MHz, $CDCl_3$ + TFA-D)

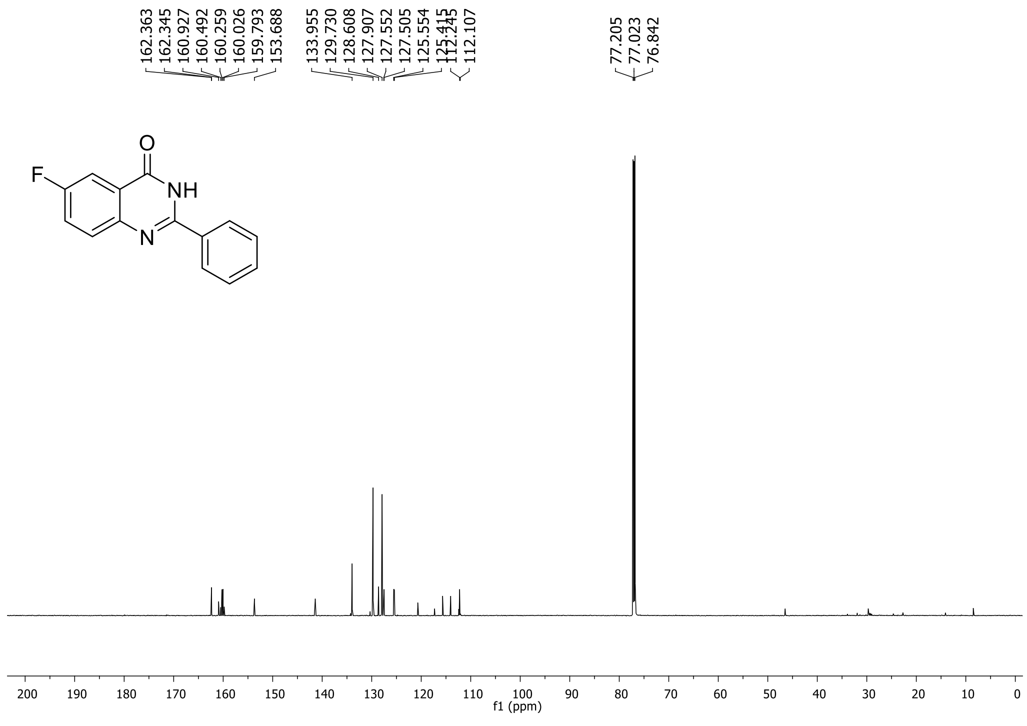

Figure S55. ^{13}C NMR of 6-fluoro-2-phenylquinazolin-4(3*H*)-one **(6a)**

^{1}H NMR (700 MHz, $CDCl_3$ + TFA-d)

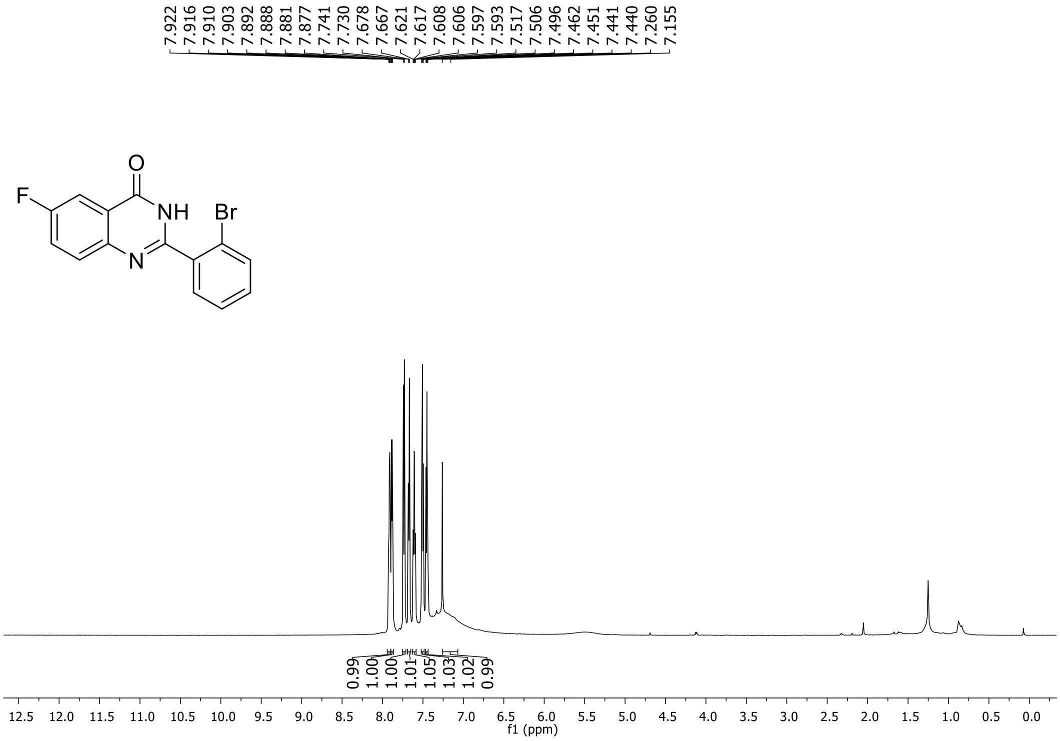

Figure S56. ^{1}H NMR of 2-(2-bromophenyl)-6-fluoroquinazolin-4(3*H*)-one **(6b)**

^{13}C NMR (175 MHz, $CDCl_3$ + TFA-d)

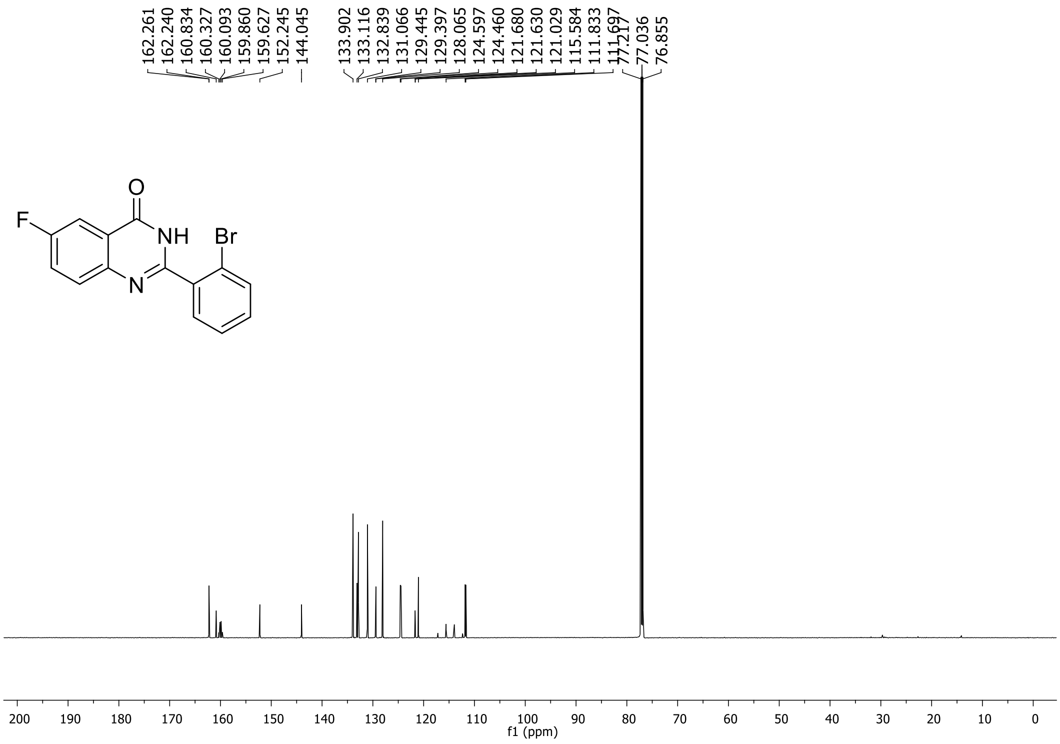

Figure S57. ^{13}C NMR of 2-(2-bromophenyl)-6-fluoroquinazolin-4(3*H*)-one **(6b)**

^{1}H NMR (400 MHz, $CDCl_3$ + TFA-d)

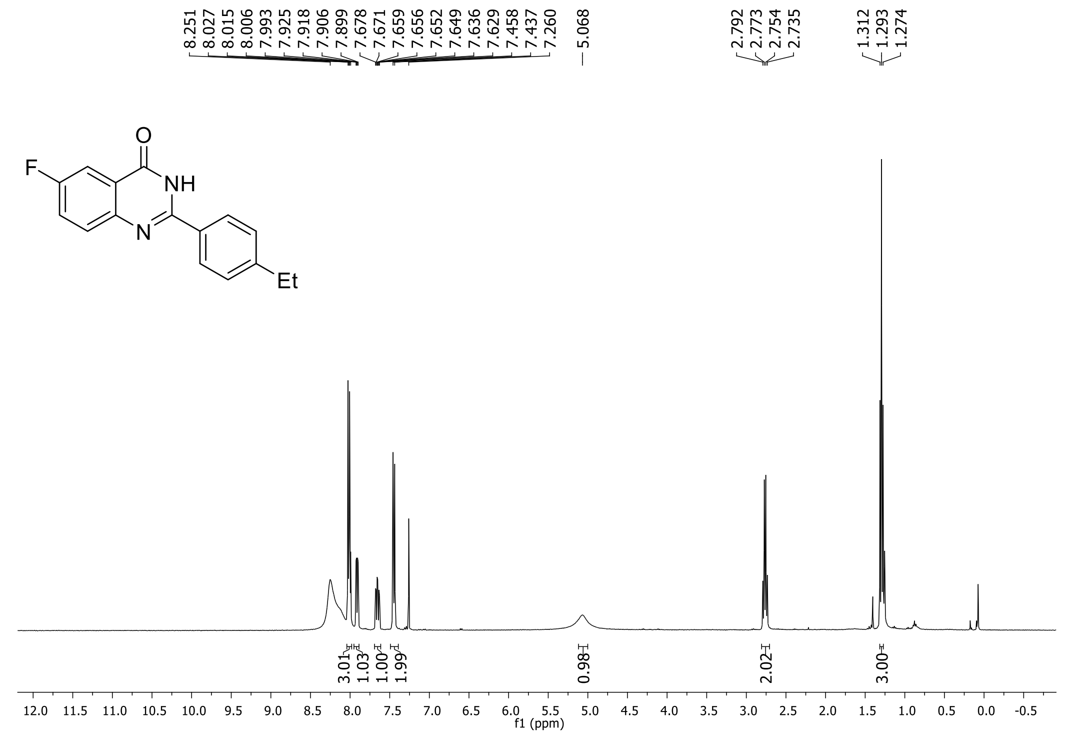

Figure S58. ^{1}H NMR of 2-(4-ethylphenyl)-6-fluoroquinazolin-4(3*H*)-one **(6c)**

^{13}C NMR (100 MHz, $CDCl_3$ + TFA-d)

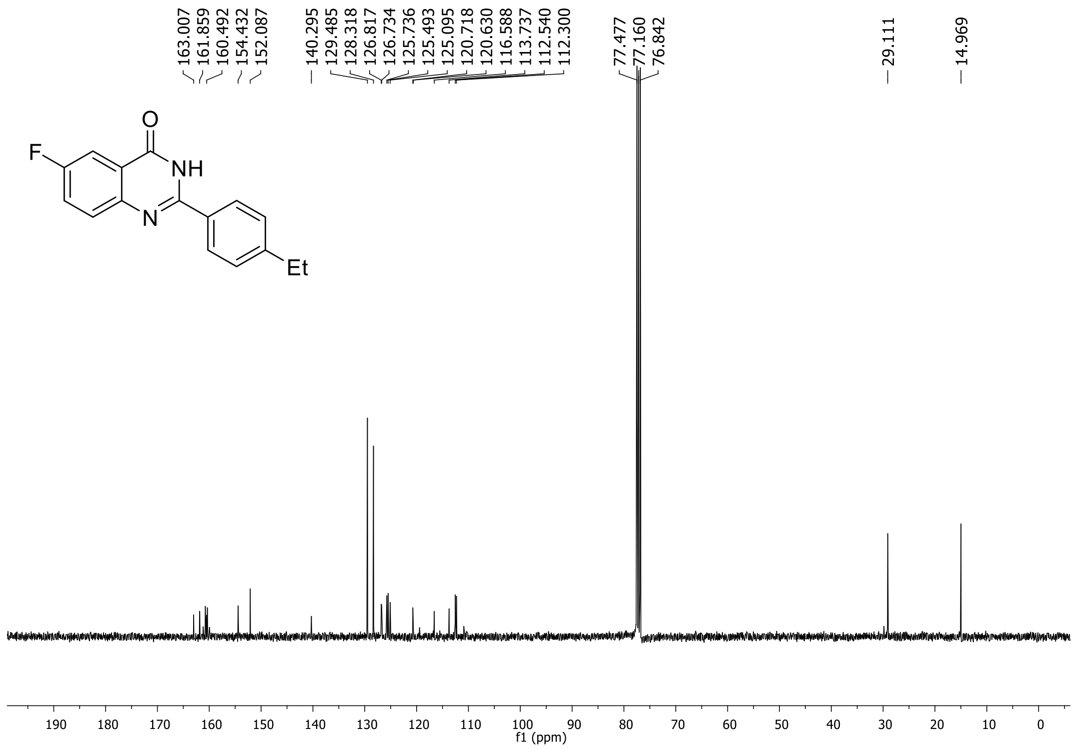

Figure S59. ^{13}C NMR of 2-(4-ethylphenyl)-6-fluoroquinazolin-4(3*H*)-one **(6c)**

^{1}H NMR (700 MHz, $CDCl_3$)

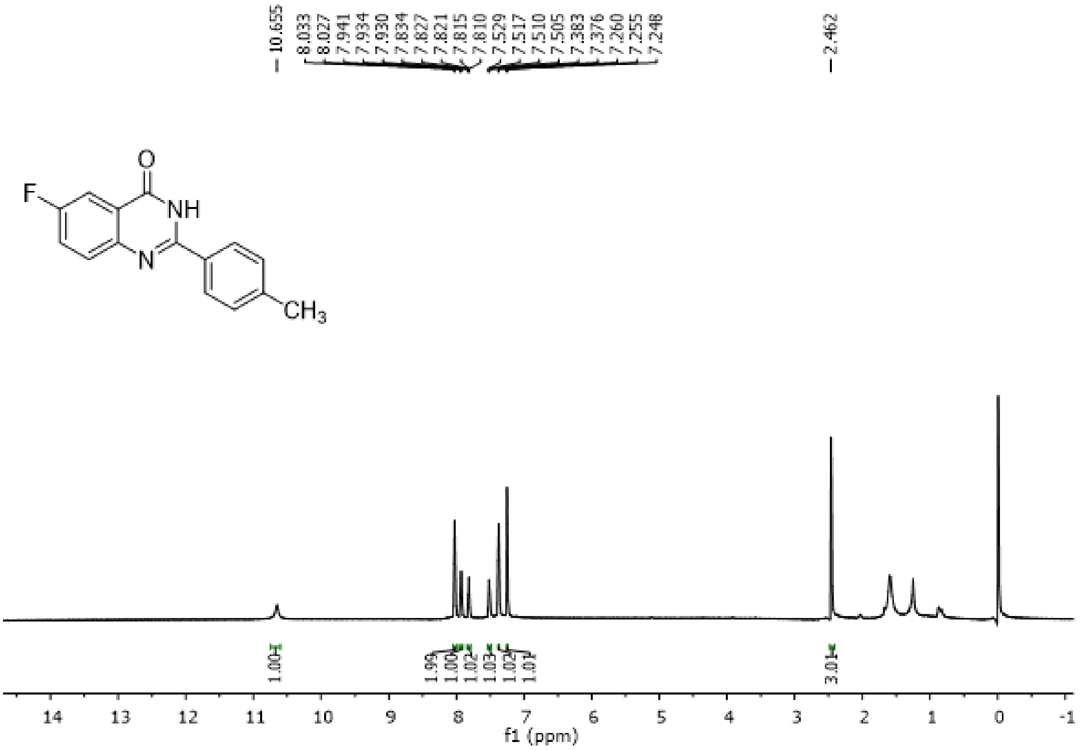

Figure S60. ^{1}H NMR of 6-fluoro-2-(*p*-tolyl)quinazolin-4(3*H*)-one **(6d)**

^{13}C NMR (175 MHz, $CDCl_3$)

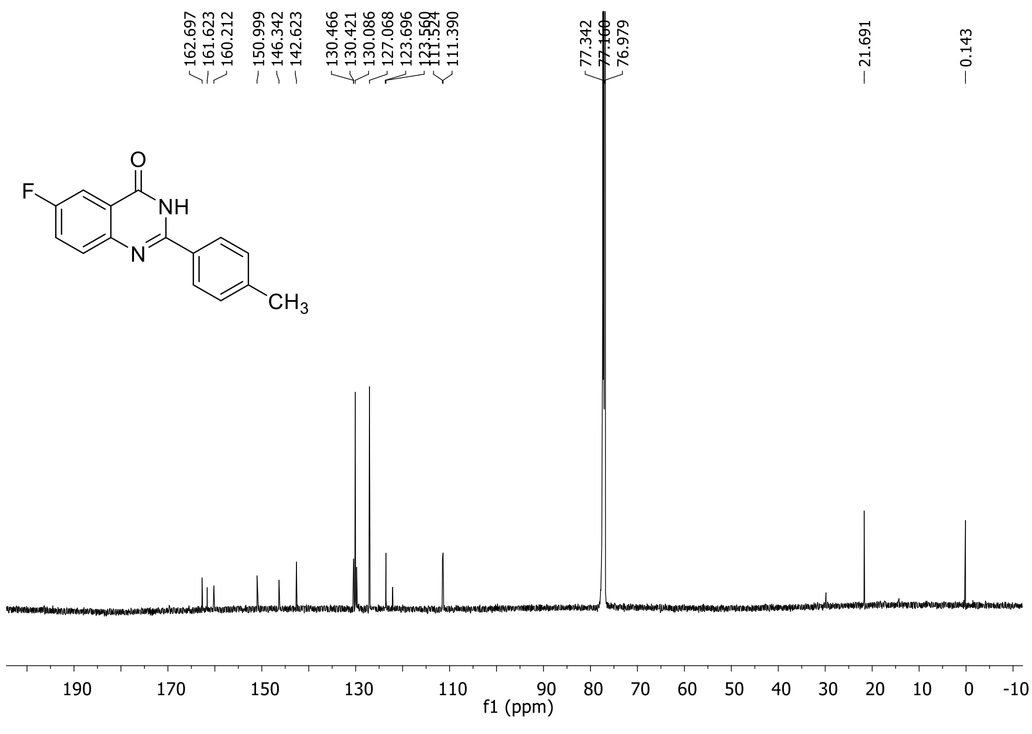

Figure S61. ^{13}C NMR of 6-fluoro-2-(*p*-tolyl)quinazolin-4(3*H*)-one **(6d)**

^{1}H NMR (400 MHz, DMSO-d$_6$)

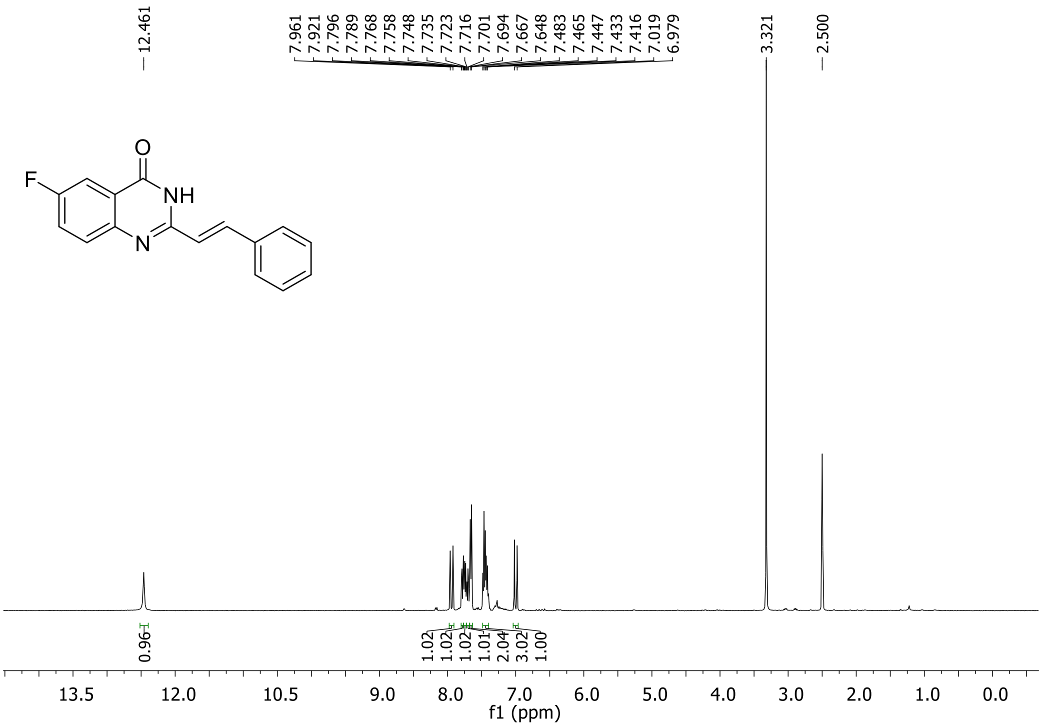

Figure S62. ^{1}H NMR of (*E*)-6-fluoro-2-styrylquinazolin-4(3*H*)-one **(6e)**

^{13}C NMR (100 MHz, DMSO-d$_6$)

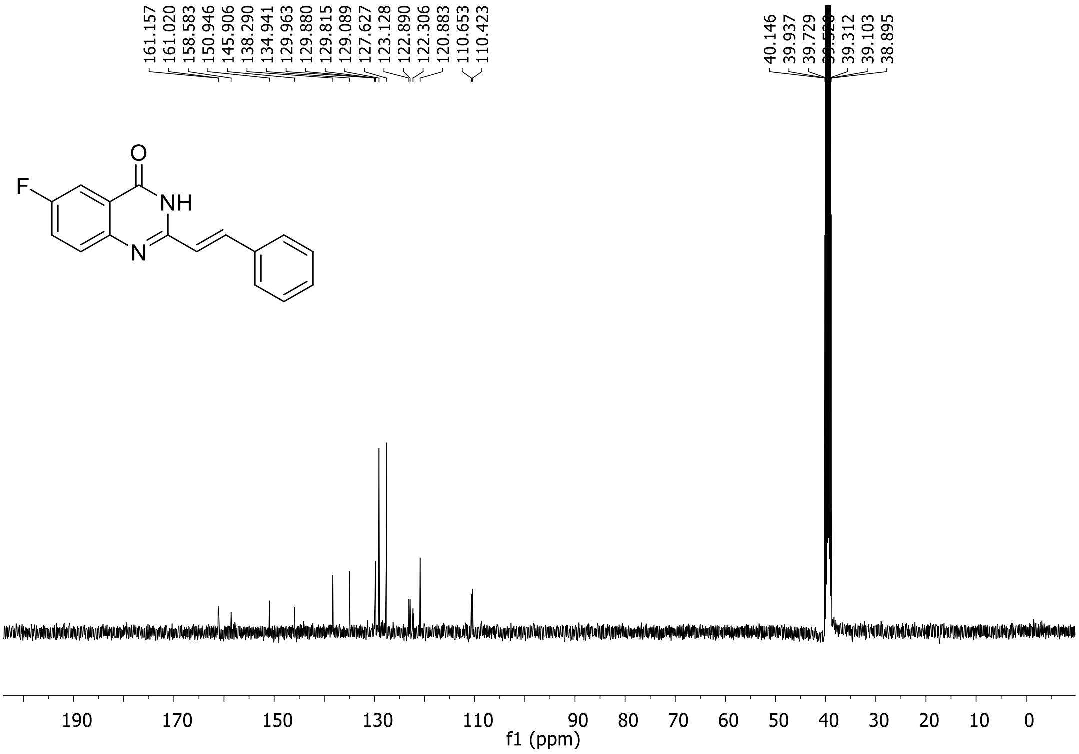

Figure S63. ^{13}C NMR of (*E*)-6-fluoro-2-styrylquinazolin-4(3*H*)-one **(6e)**

^{1}H NMR (700 MHz, DMSO-d$_6$)

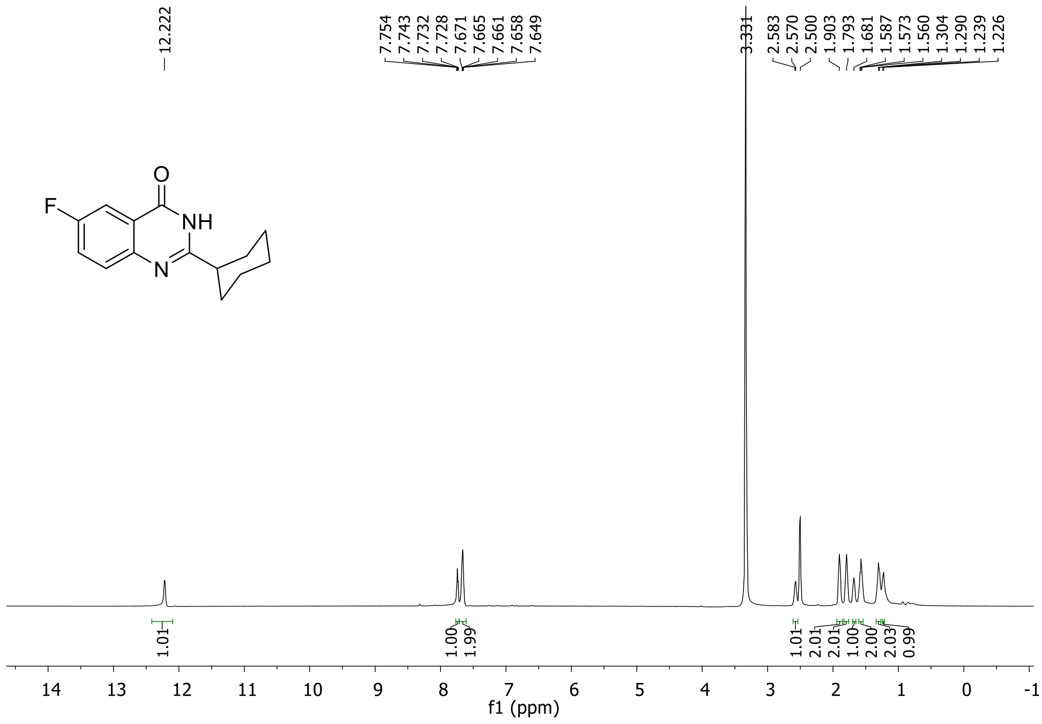

Figure S64. ^{1}H NMR of 2-cyclohexyl-6-fluoroquinazolin-4(3*H*)-one **(6f)**

^{13}C NMR (175 MHz, DMSO-d$_6$)

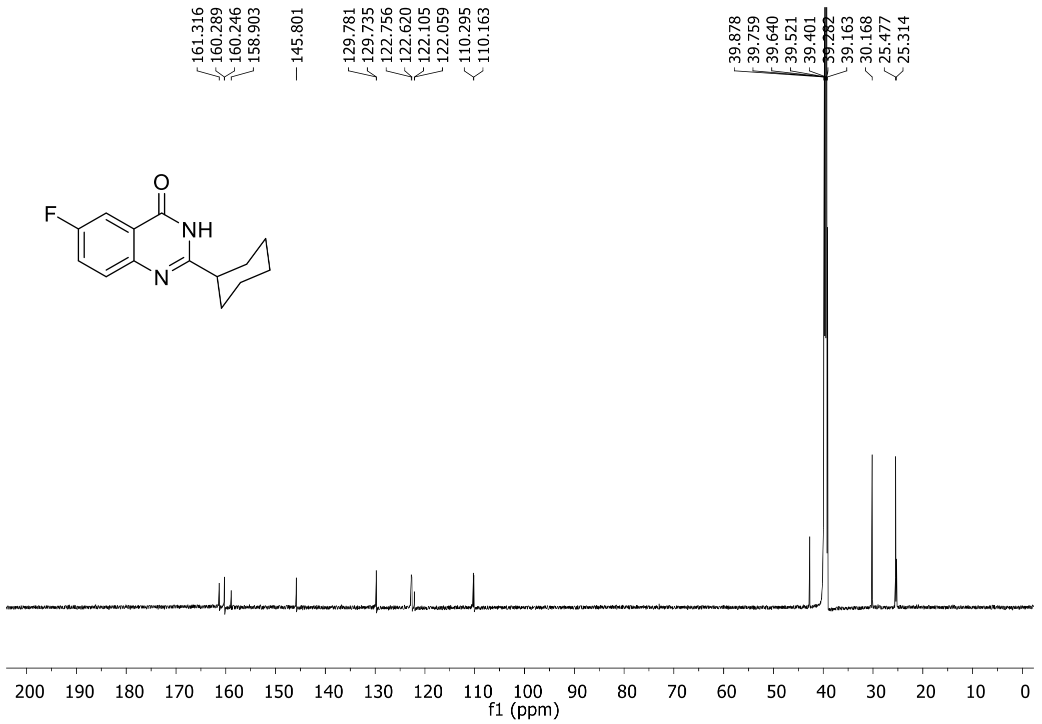

Figure S65. ^{13}C NMR of 2-cyclohexyl-6-fluoroquinazolin-4(3*H*)-one **(6f)**

^{1}H NMR (400 MHz, $CDCl_3$)

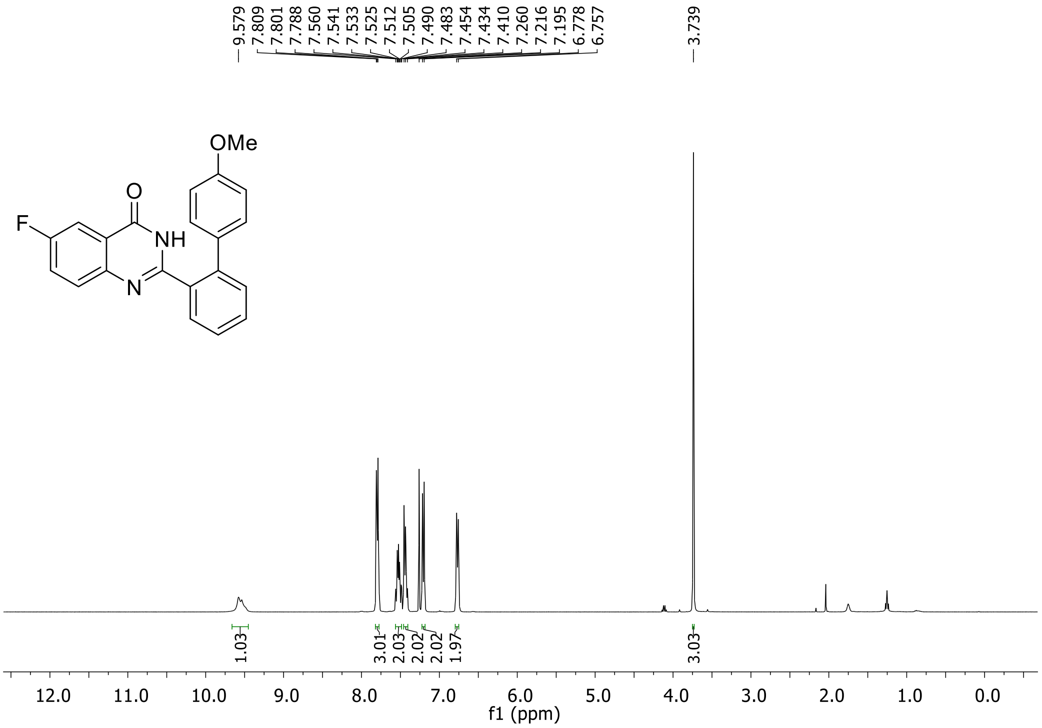

Figure S66. ^{1}H NMR of 6-fluoro-2-(4'-methoxy-[1,1'-biphenyl]-2-yl)quinazolin-4(3*H*)-one **(6g)**

^{13}C NMR (100 MHz, $CDCl_3$)

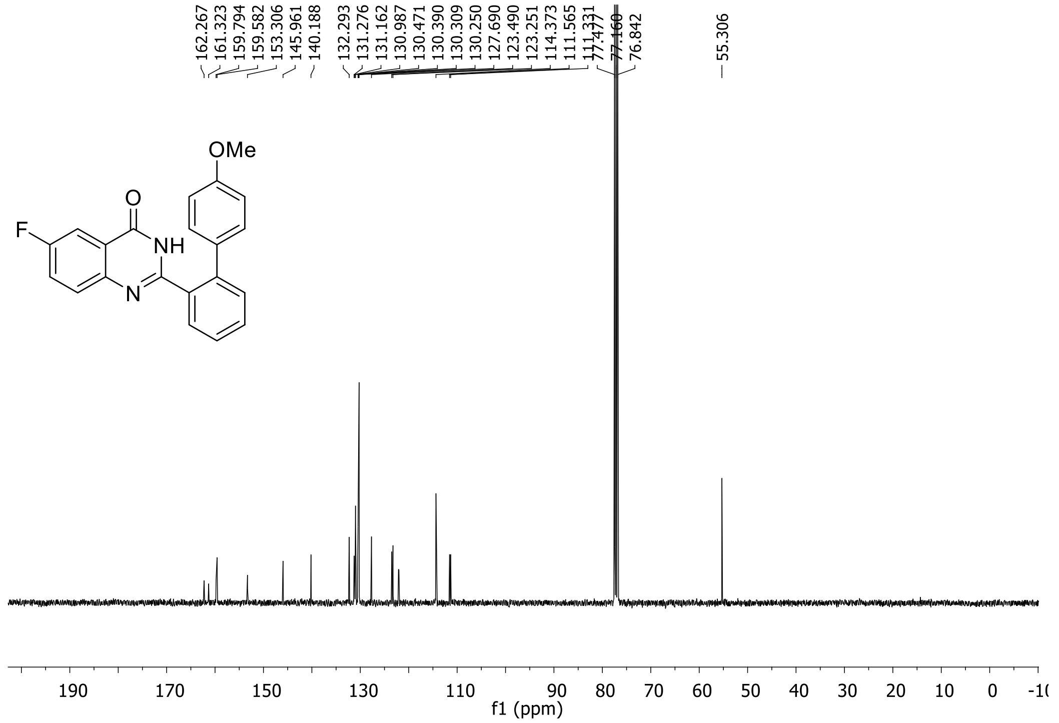

Figure S67. ^{13}C NMR of 6-fluoro-2-(4'-methoxy-[1,1'-biphenyl]-2-yl)quinazolin-4(3*H*)-one **(6g)**

^{1}H NMR (400 MHz, CDCl$_3$)

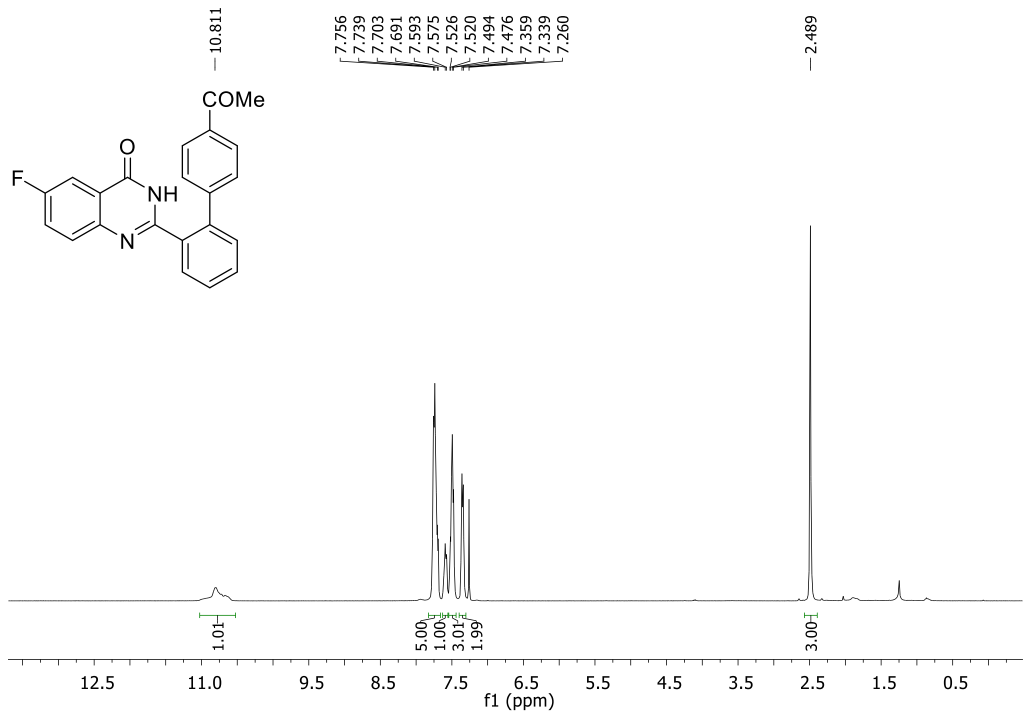

Figure S68. ^{1}H NMR of 2-(4'-acetyl-[1,1'-biphenyl]-2-yl)-6-fluoroquinazolin-4(3*H*)-one **(6h)**

^{13}C NMR (100 MHz, CDCl$_3$)

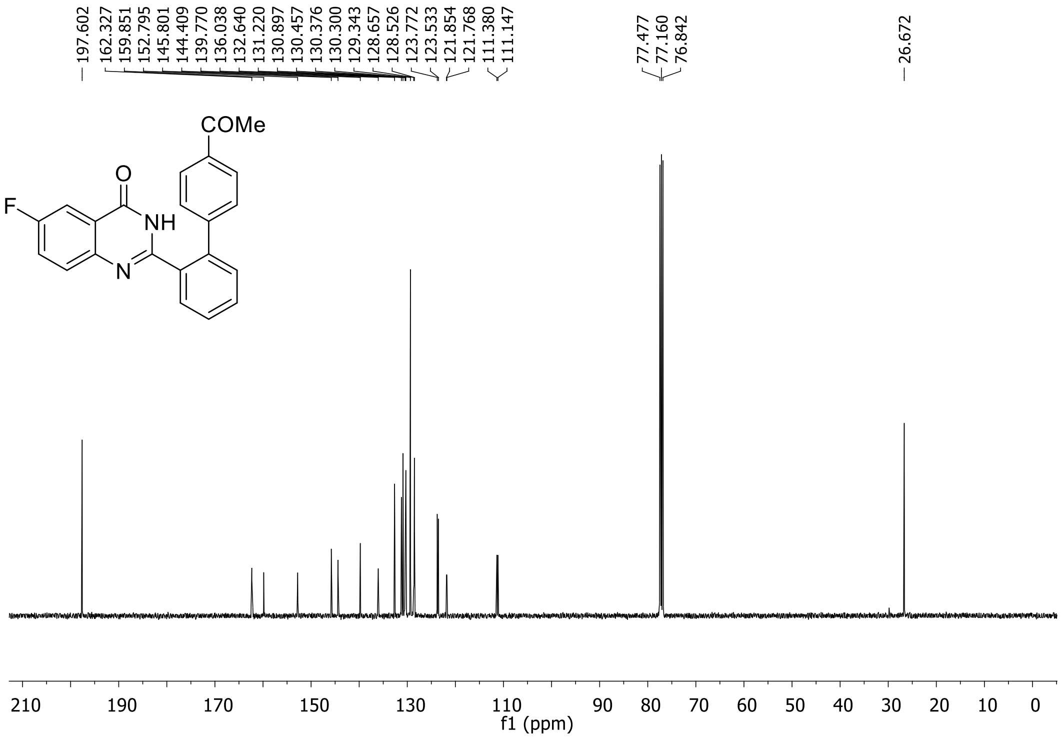

Figure S69. ^{13}C NMR of 2-(4'-acetyl-[1,1'-biphenyl]-2-yl)-6-fluoroquinazolin-4(3*H*)-one **(6h)**

^{1}H NMR (700 MHz, DMSO-d$_6$)

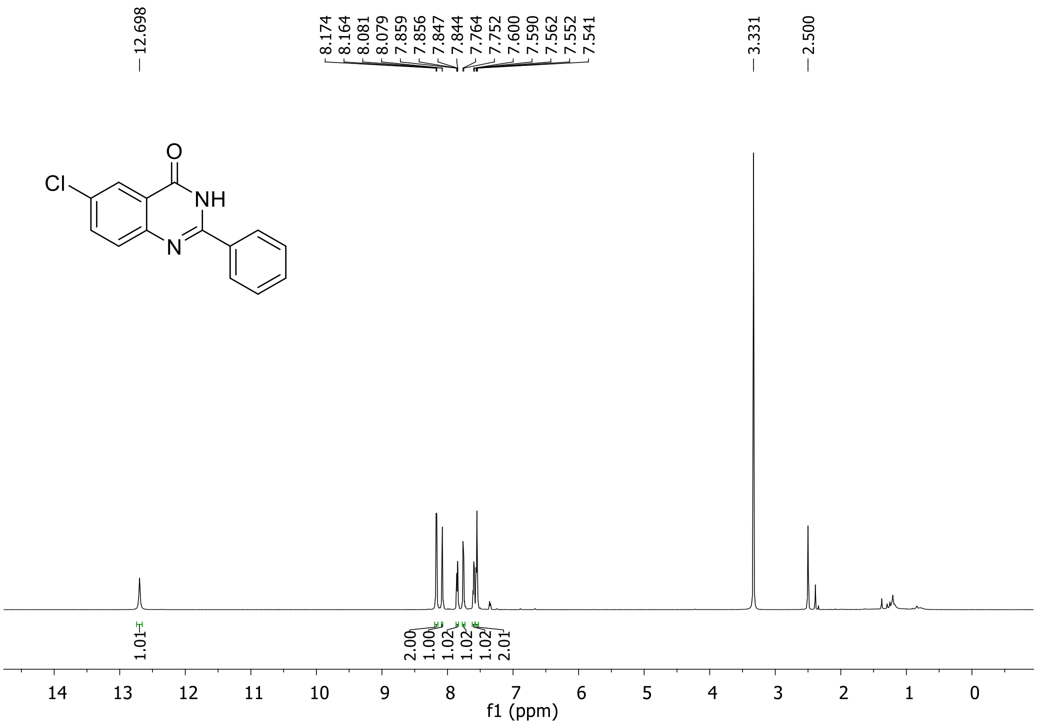

Figure S70. ^{1}H NMR of 6-chloro-2-phenylquinazolin-4(3*H*)-one **(6i)**

^{13}C NMR (175 MHz, DMSO-d$_6$)

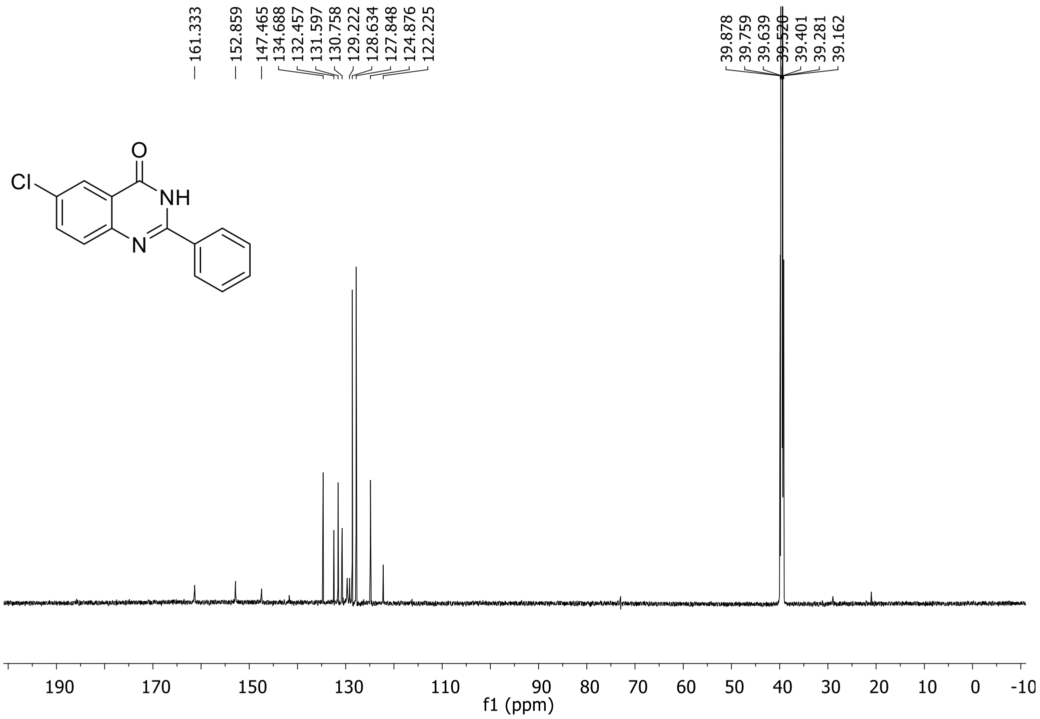

Figure S71. ^{13}C NMR of 6-chloro-2-phenylquinazolin-4(3*H*)-one **(6i)**

^{1}H NMR (400 MHz, $CDCl_3$ + DMSO-d_6)

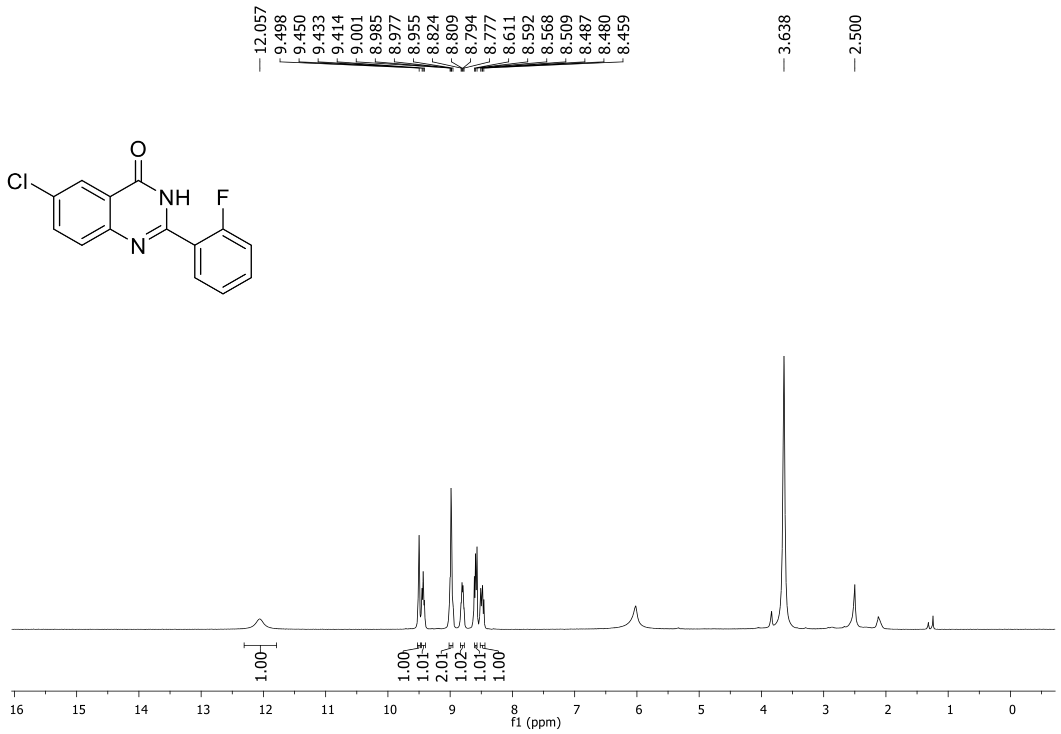

Figure S72. ^{1}H NMR of 6-chloro-2-(2-fluorophenyl)quinazolin-4(3*H*)-one **(6j)**

^{13}C NMR (100 MHz, $CDCl_3$ + DMSO-d_6)

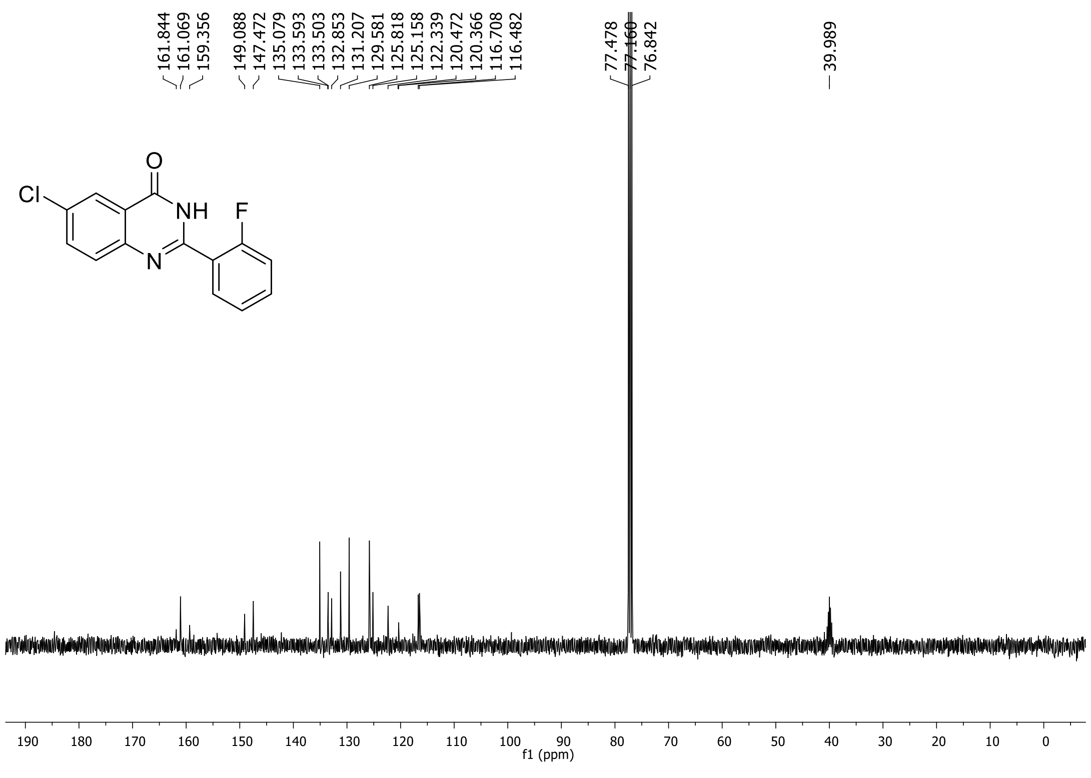

Figure S73. ^{13}C NMR of 6-chloro-2-(2-fluorophenyl)quinazolin-4(3*H*)-one **(6j)**

^{1}H NMR (400 MHz, $CDCl_3$ + DMSO-d_6)

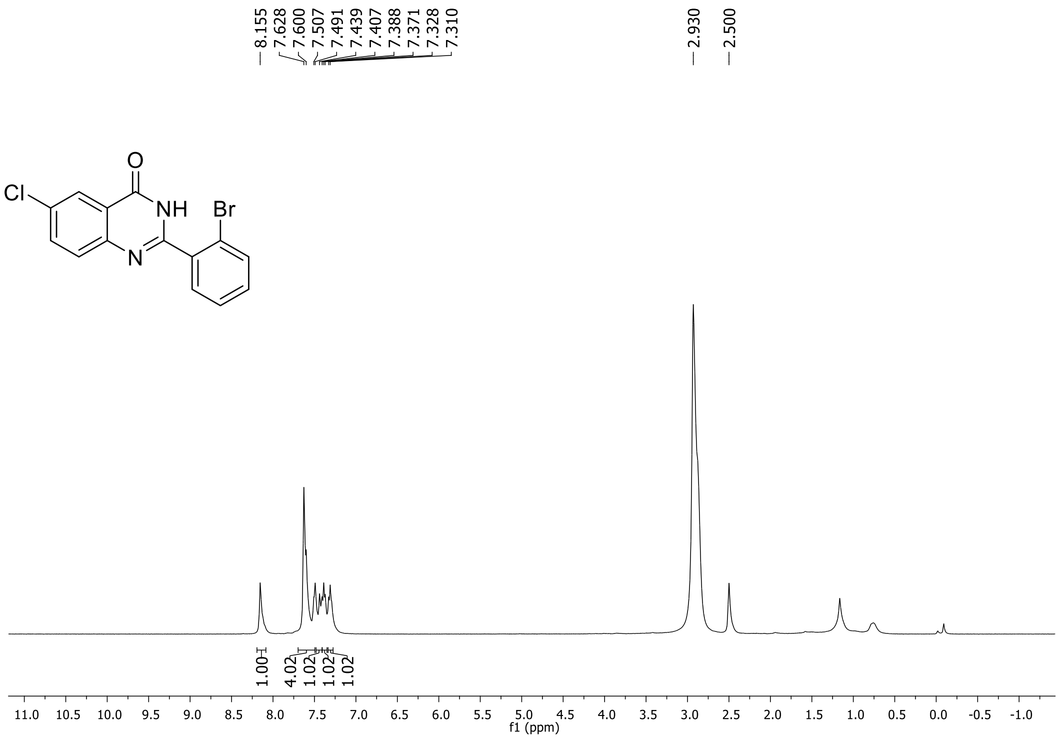

Figure S74. ^{1}H NMR of 2-(2-bromophenyl)-6-chloroquinazolin-4(3*H*)-one **(6k).**

^{13}C NMR (100 MHz, $CDCl_3$ + DMSO-d_6)

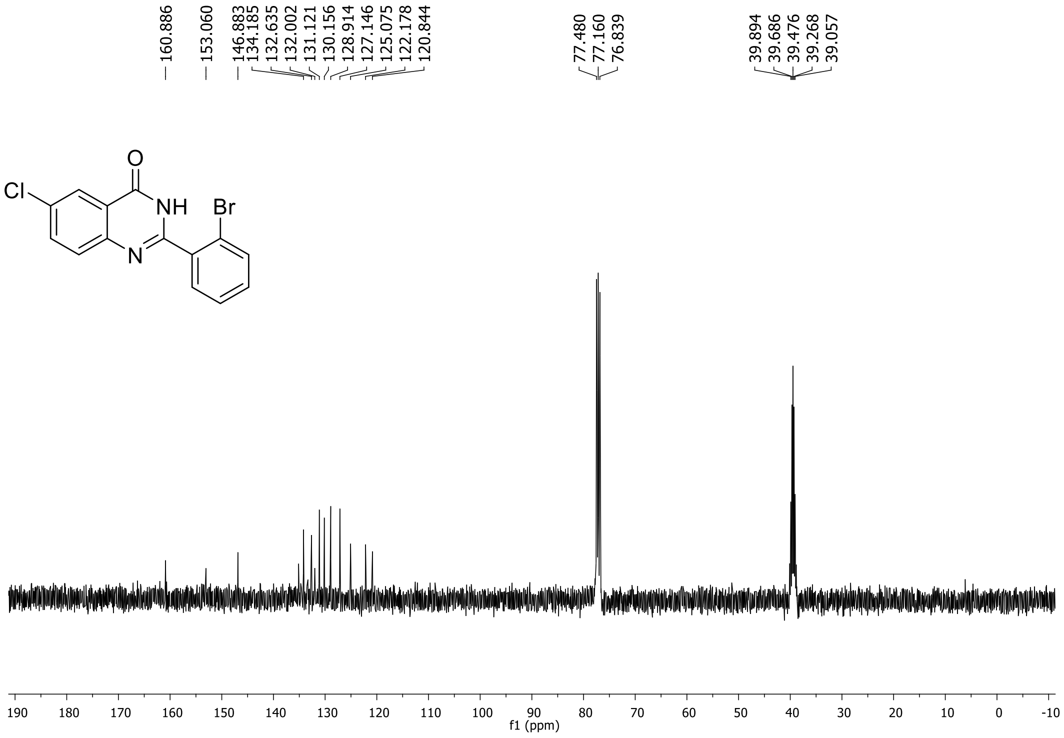

Figure S75. ^{13}C NMR of 2-(2-bromophenyl)-6-chloroquinazolin-4(3*H*)-one **(6k).**

^{1}H NMR (400 MHz, DMSO-d$_6$)

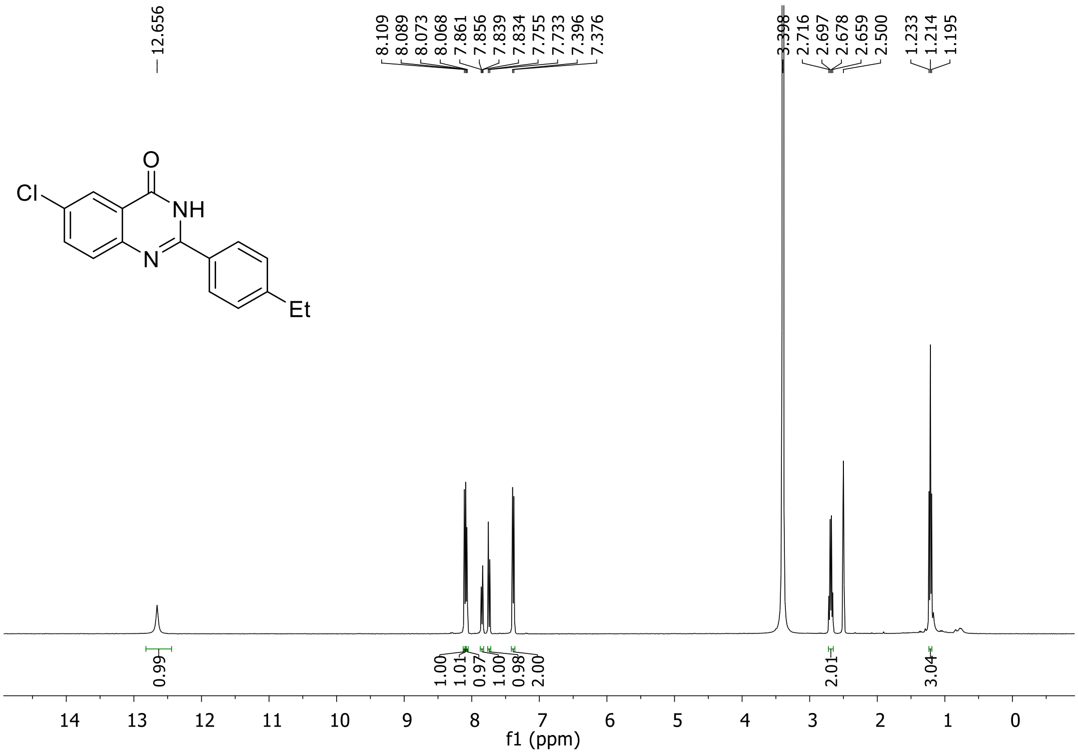

Figure S76. ^{1}H NMR of 6-chloro-2-(4-ethylphenyl)quinazolin-4(3*H*)-one (**6l)**

^{13}C NMR (101 MHz, DMSO-d$_6$)

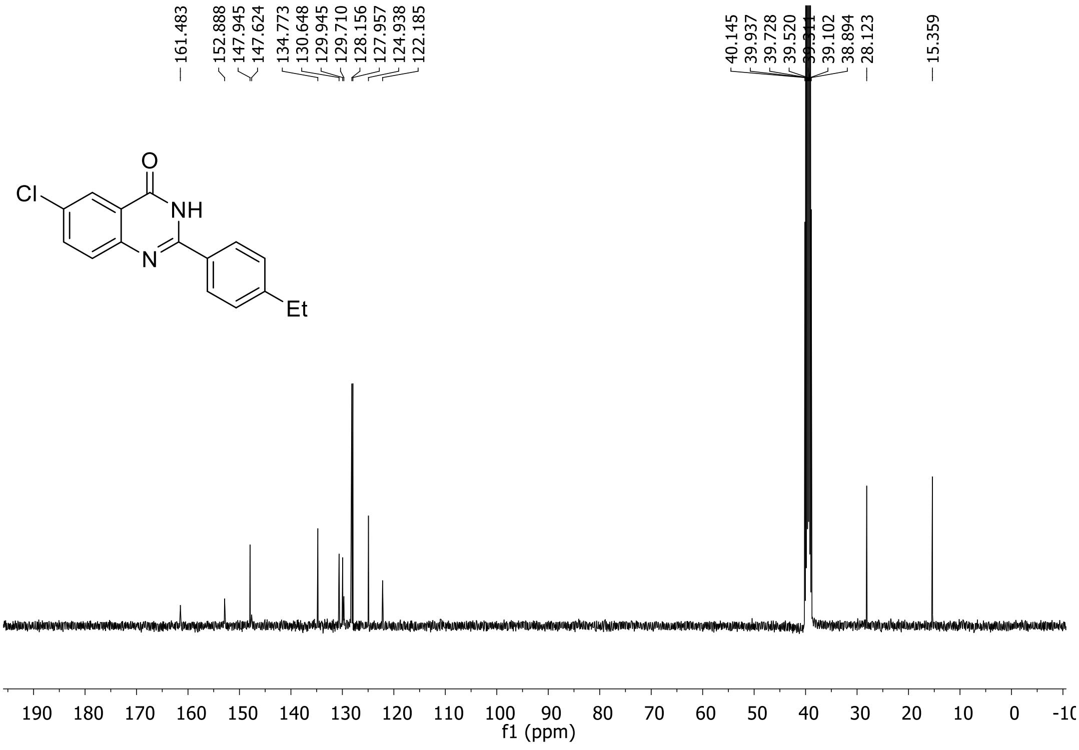

Figure S77. ^{13}C NMR of 6-chloro-2-(4-ethylphenyl)quinazolin-4(3*H*)-one (**6l)**

^{1}H NMR (400 MHz, DMSO-d_6)

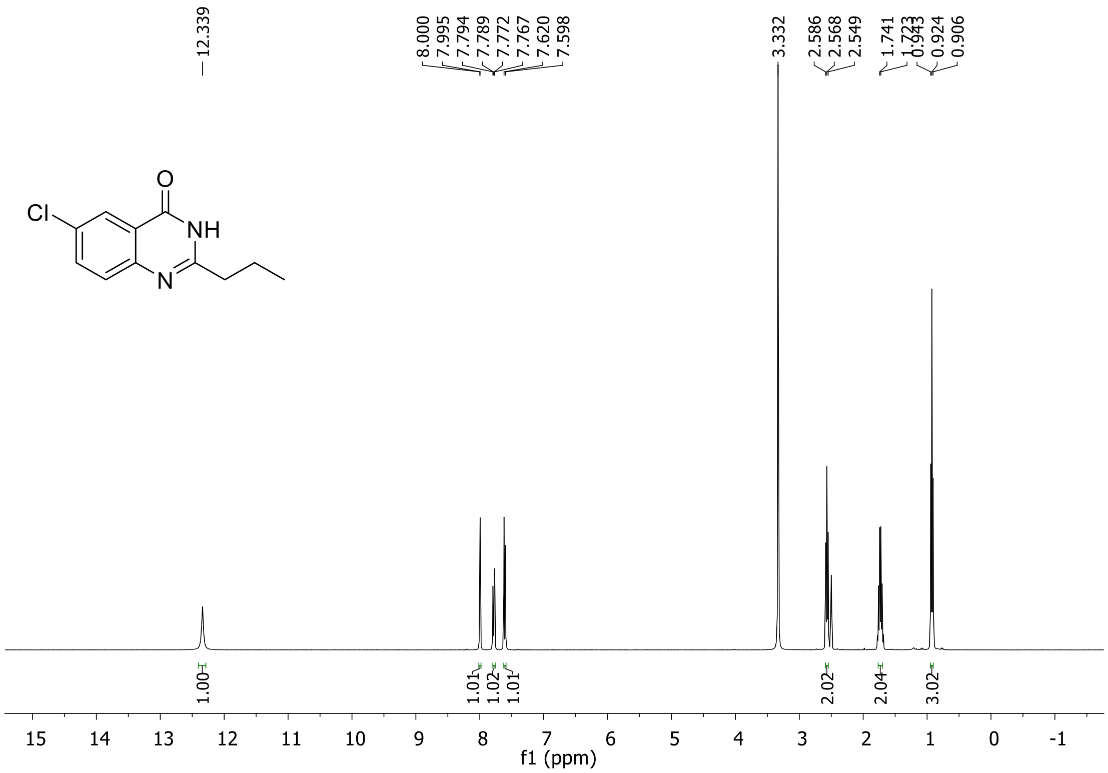

Figure S78. ^{1}H NMR of 6-chloro-2-propylquinazolin-4(3*H*)-one **(6m)**

^{13}C NMR (100 MHz, DMSO-d_6)

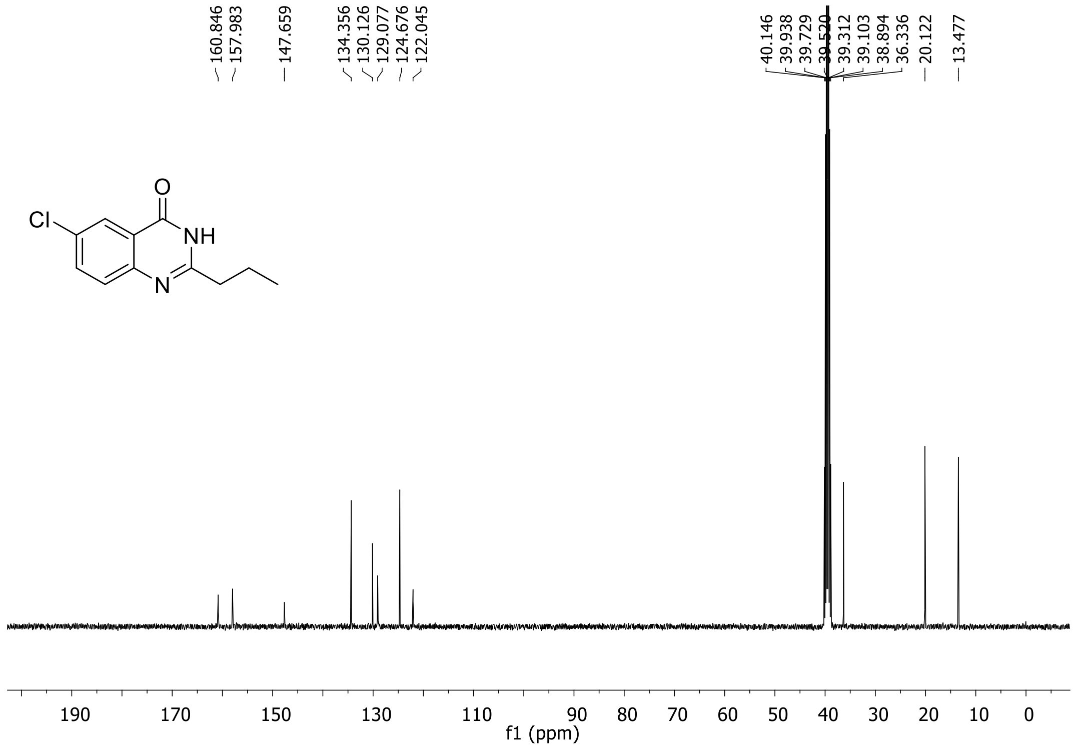

Figure S79. ^{13}C NMR of 6-chloro-2-propylquinazolin-4(3*H*)-one **(6m)**

^{1}H NMR (400 MHz, CDCl$_3$)

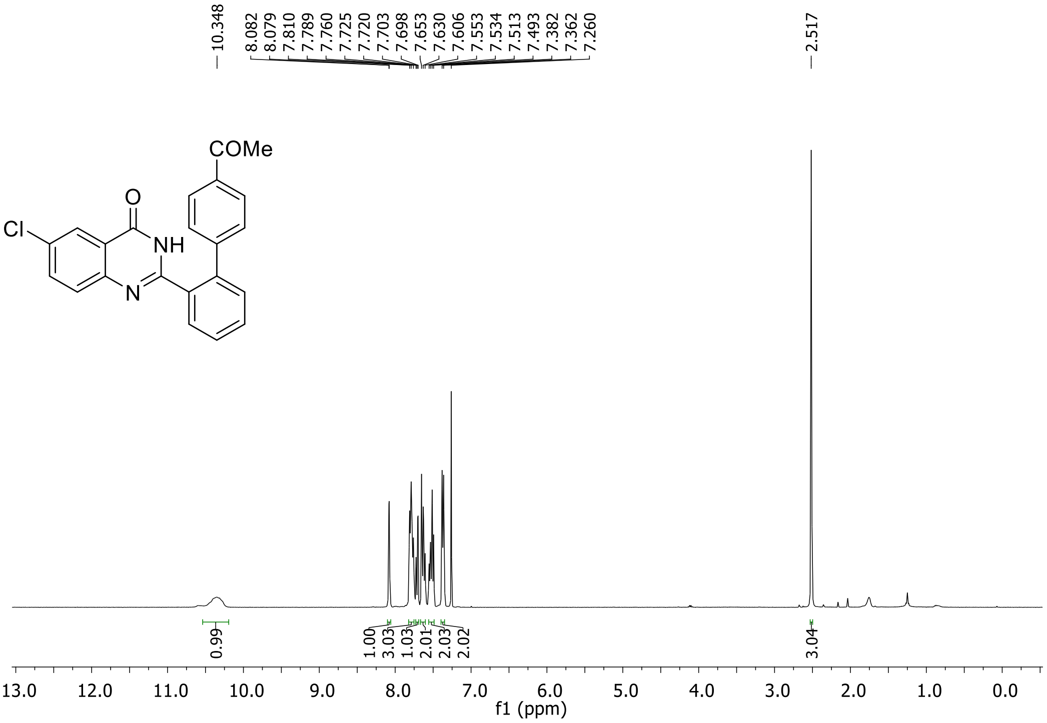

Figure S80. ^{1}H NMR of 2-(4'-acetyl-[1,1'-biphenyl]-2-yl)-6-chloroquinazolin-4(3*H*)-one **(6n)**

^{13}C NMR (100 MHz, CDCl$_3$)

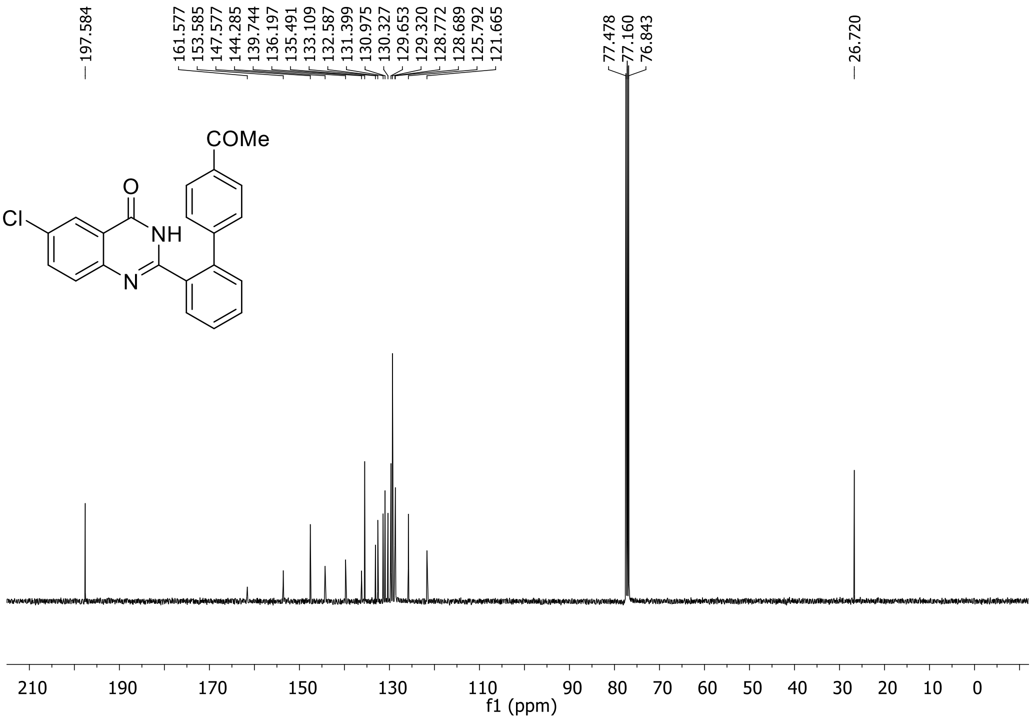

Figure S81. ^{13}C NMR of 2-(4'-acetyl-[1,1'-biphenyl]-2-yl)-6-chloroquinazolin-4(3*H*)-one **(6n)**

^{1}H NMR (400 MHz, CDCl$_3$)

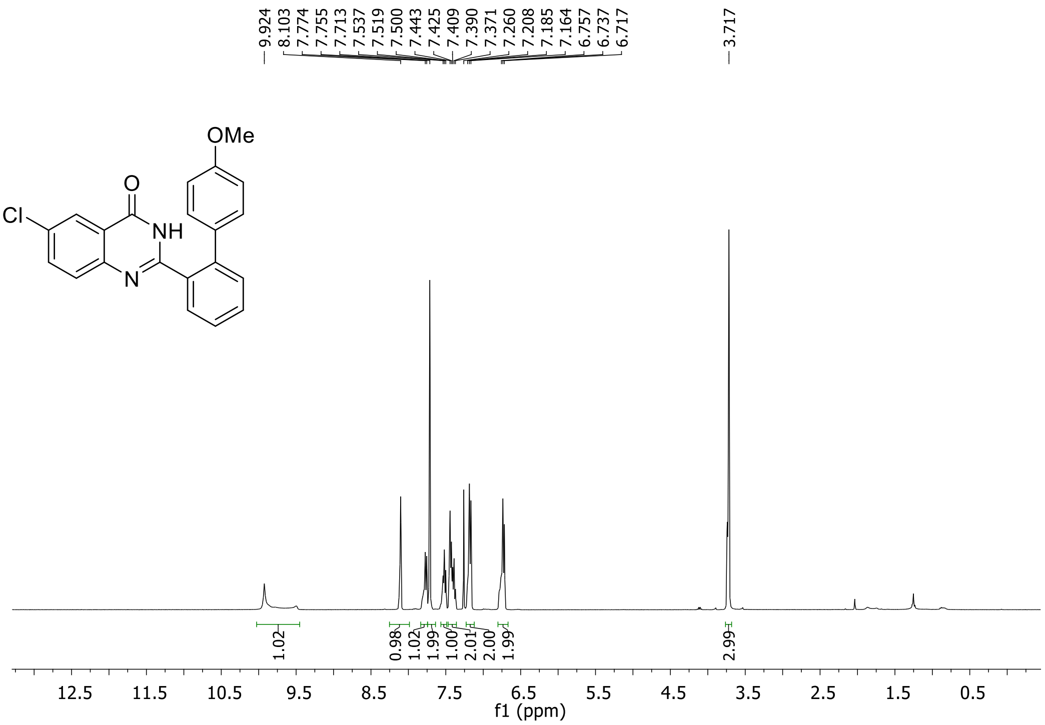

Figure S82. ^{1}H NMR of 6-chloro-2-(4'-methoxy-[1,1'-biphenyl]-2-yl)quinazolin-4(3*H*)-one **(6o)**

^{13}C NMR (100 MHz, CDCl$_3$)

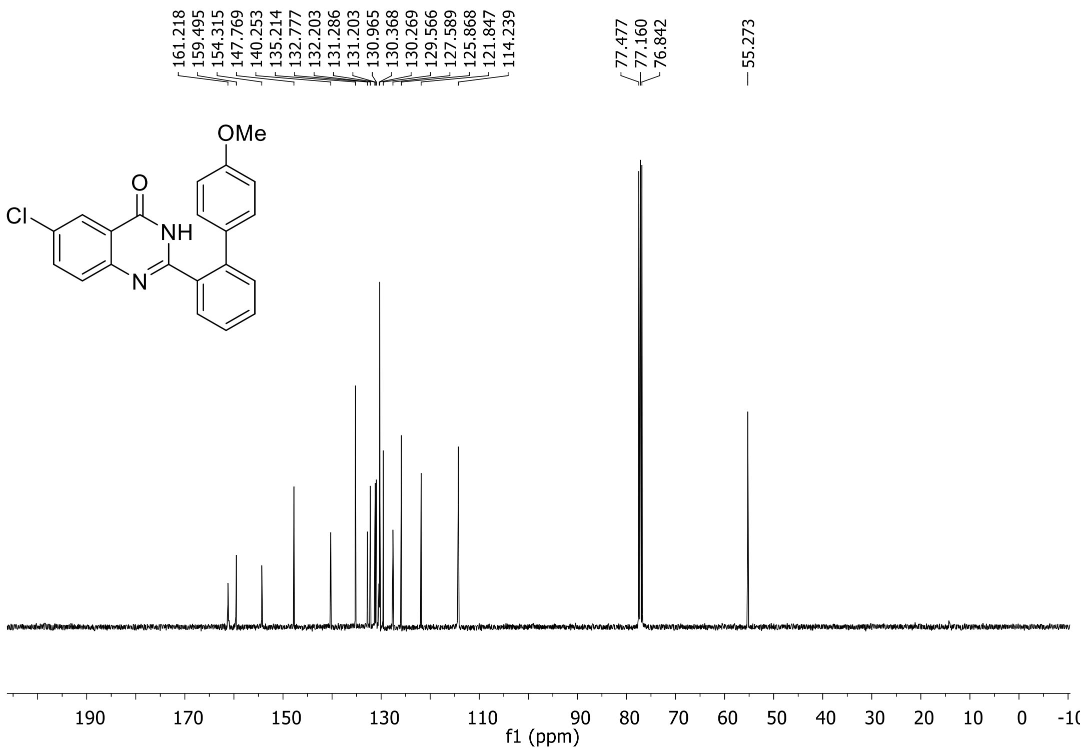

Figure S83. ^{13}C NMR of 6-chloro-2-(4'-methoxy-[1,1'-biphenyl]-2-yl)quinazolin-4(3*H*)-one **(6o)**

^{1}H NMR (400 MHz, CDCl$_3$)

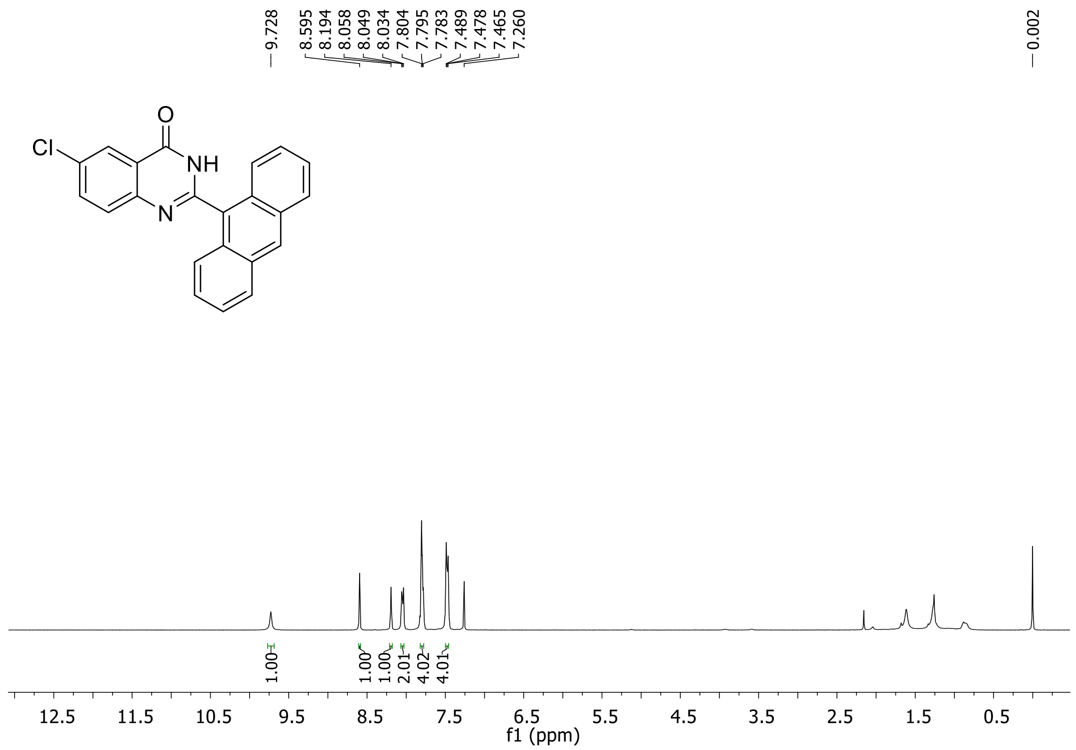

Figure S84. ^{1}H NMR of 2-(anthracen-9-yl)-6-chloroquinazolin-4(3*H*)-one **(6p)**

^{13}C NMR (100 MHz, CDCl$_3$)

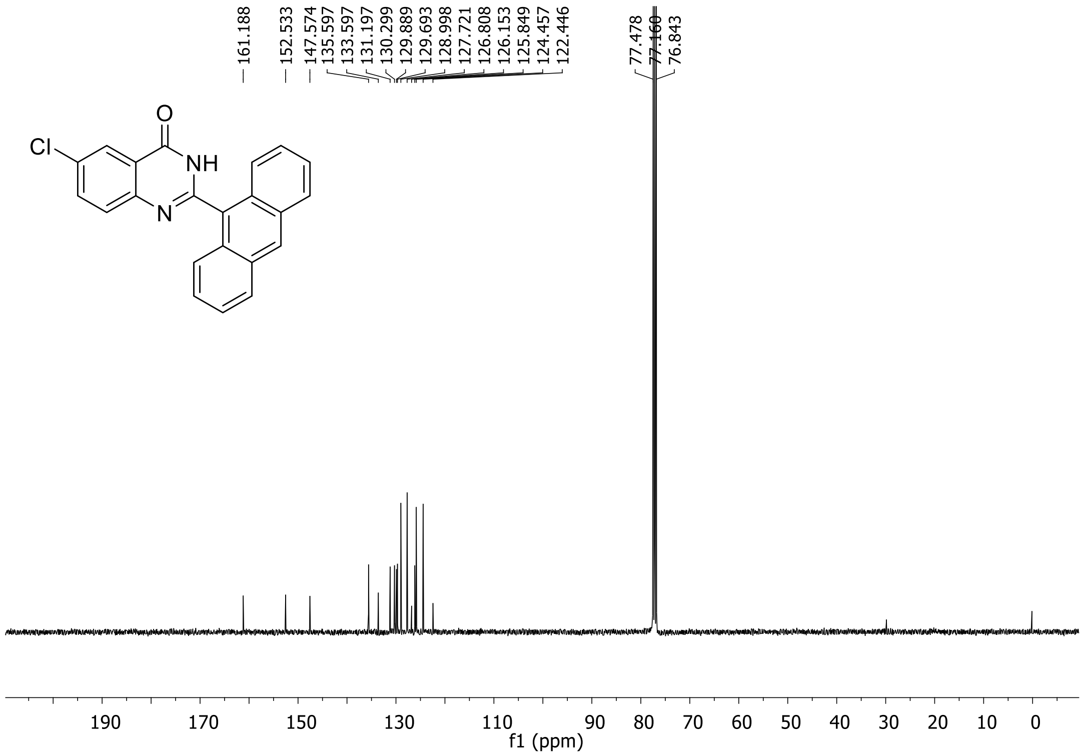

Figure S85. ^{13}C NMR of 2-(anthracen-9-yl)-6-chloroquinazolin-4(3*H*)-one **(6p)**

^{1}H NMR (400 MHz, DMSO-d$_6$)

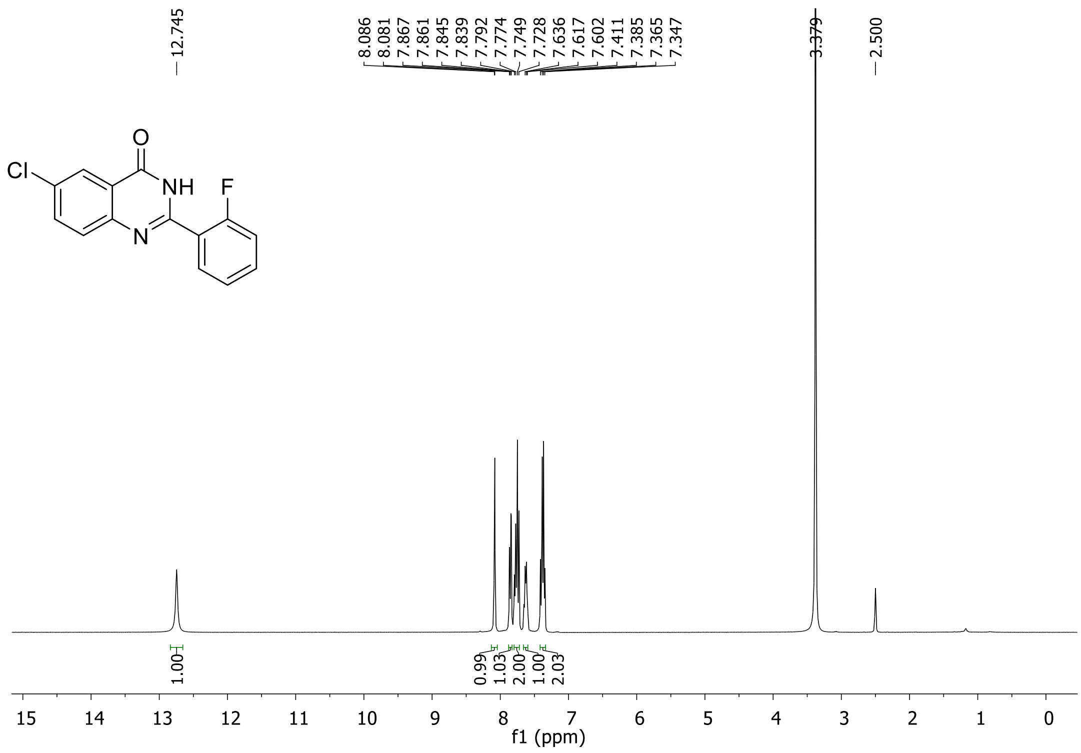

Figure S86. ^{1}H NMR of 6-chloro-2-(2-fluorophenyl)quinazolin-4(3*H*)-one **(6q)**

^{13}C NMR (100 MHz, DMSO-d$_6$)

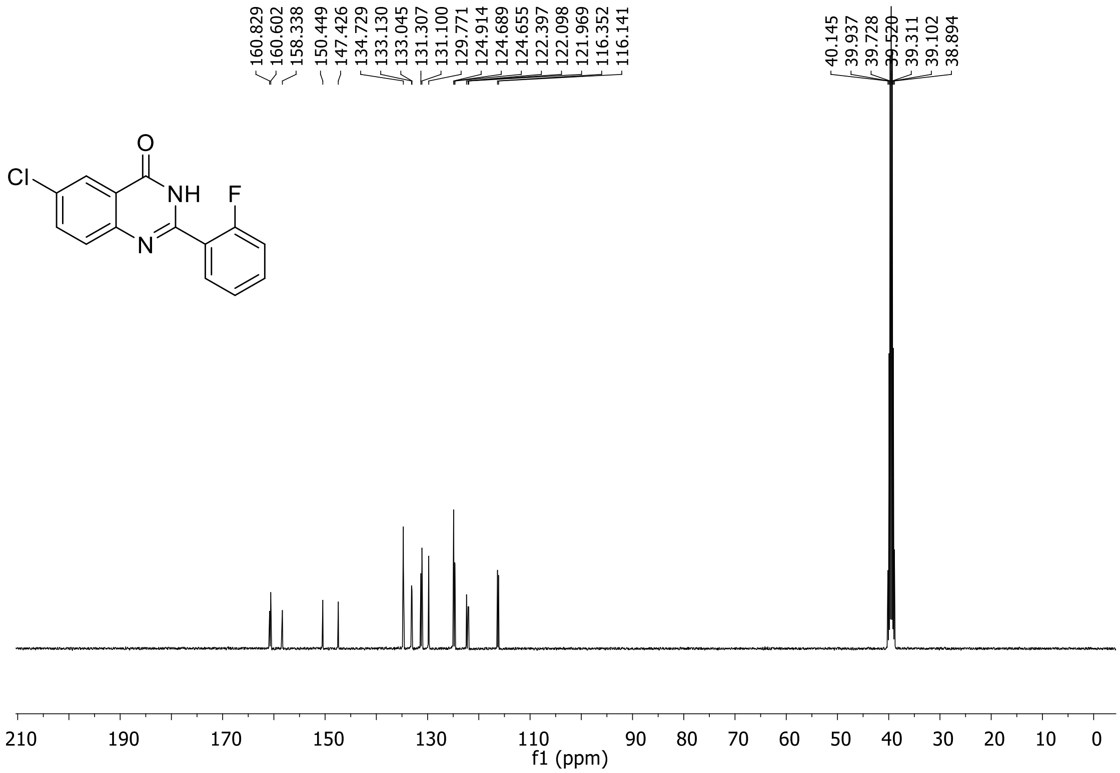

Figure S87. ^{13}C NMR of 6-chloro-2-(2-fluorophenyl)quinazolin-4(3*H*)-one **(6q)**

^{1}H NMR (400 MHz, DMSO-d_6)

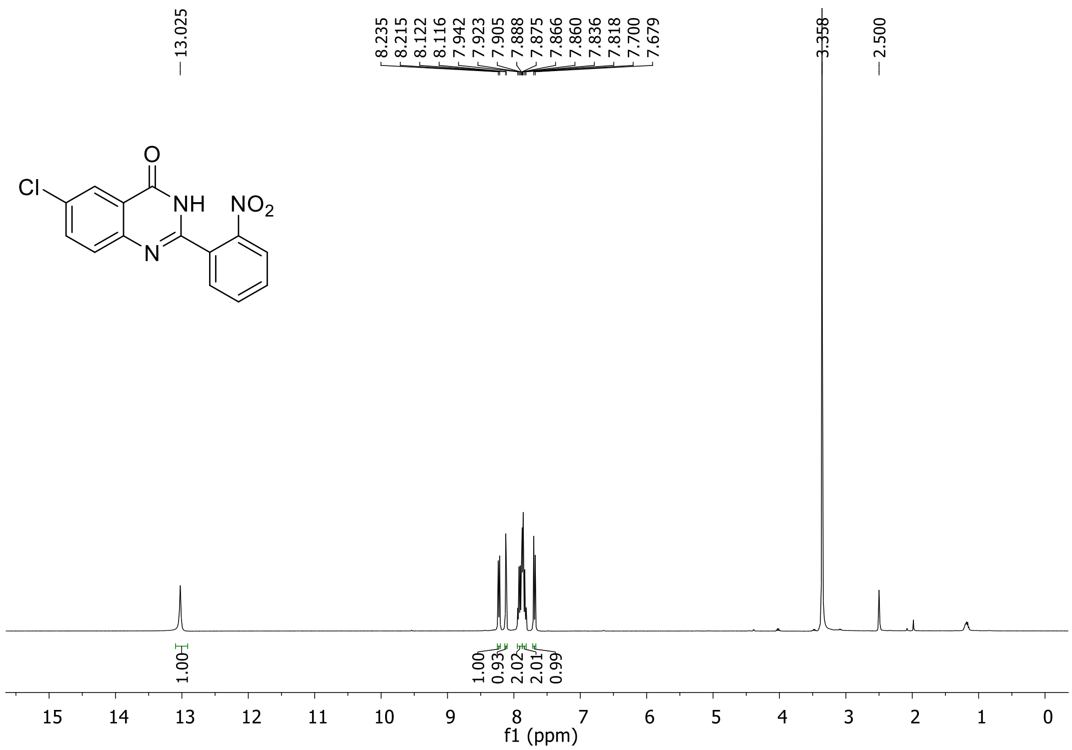

Figure S88. ^{1}H NMR of 6-chloro-2-(2-nitrophenyl)quinazolin-4(3*H*)-one **(6r)**

^{13}C NMR (100 MHz, DMSO-d_6)

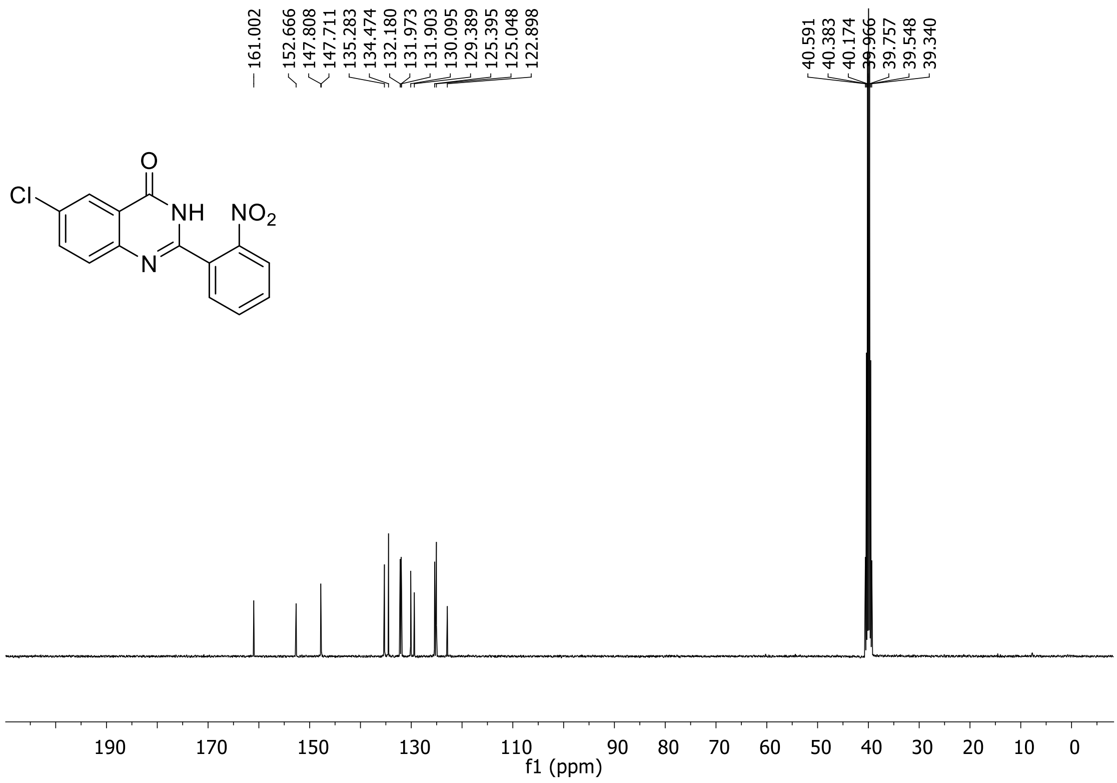

Figure S89. ^{13}C NMR of 6-chloro-2-(2-nitrophenyl)quinazolin-4(3*H*)-one **(6r)**

^{1}H NMR (400 MHz, DMSO-d$_6$)

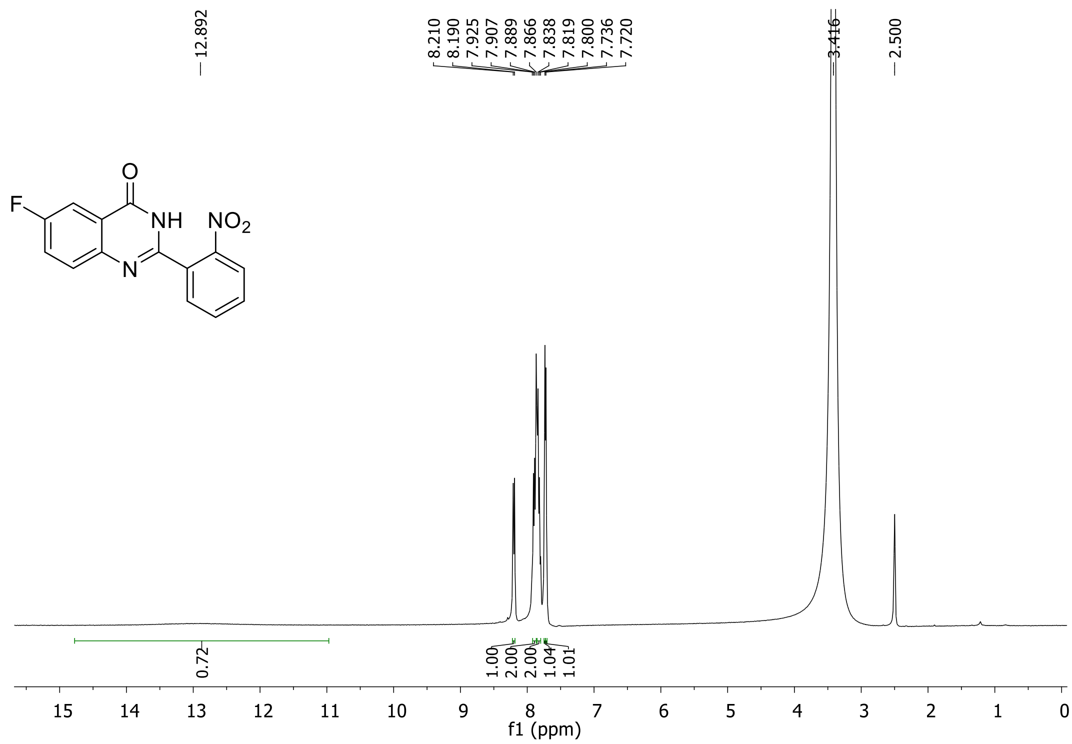

Figure S90. ^{1}H NMR of 6-fluoro-2-(2-nitrophenyl)quinazolin-4(3*H*)-one **(6s)**

^{13}C NMR (100 MHz, DMSO-d$_6$)

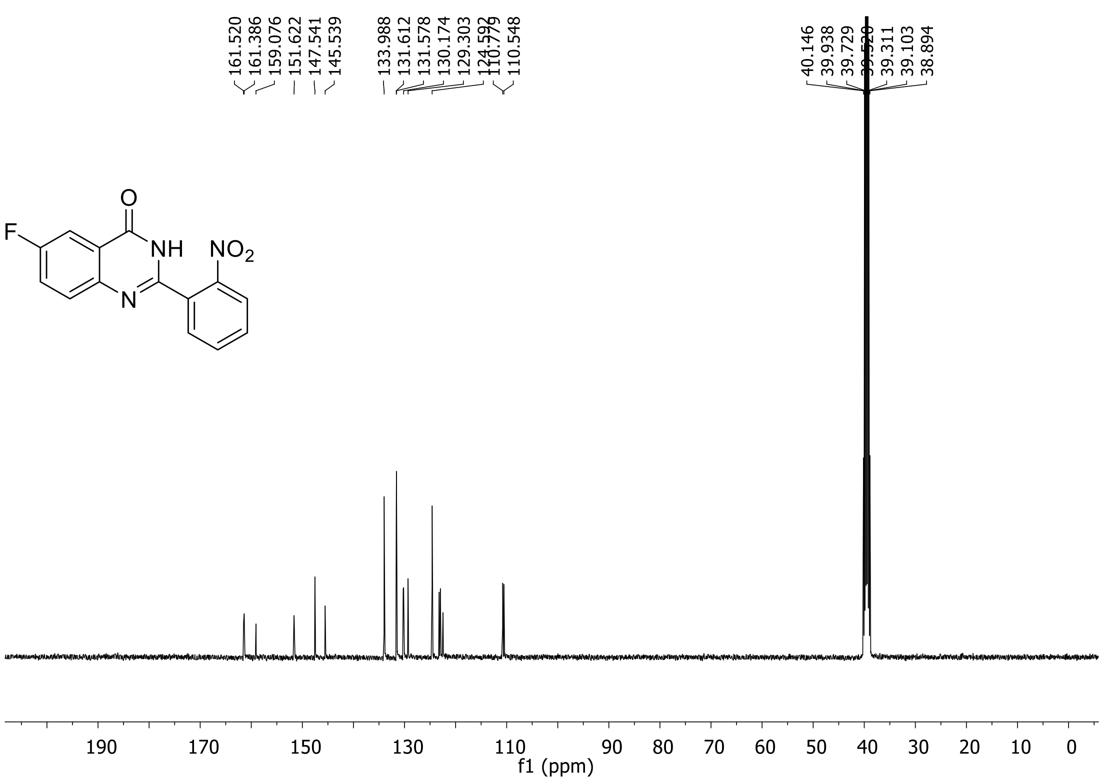

Figure S91. ^{13}C NMR of 6-fluoro-2-(2-nitrophenyl)quinazolin-4(3*H*)-one **(6s)**

^{1}H NMR (400 MHz, DMSO-d_6)

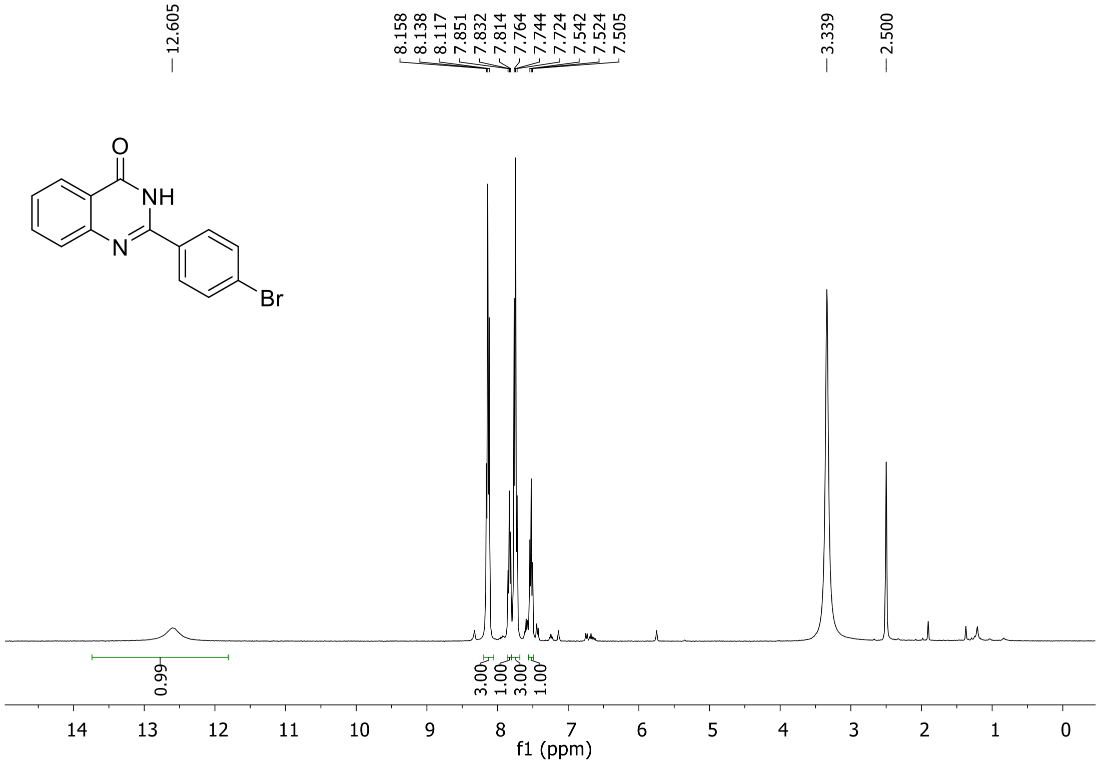

Figure S92. ^{1}H NMR of 2-(4-bromophenyl)quinazolin-4(3*H*)-one **(5m)**

^{13}C NMR (100 MHz, DMSO-d_6)

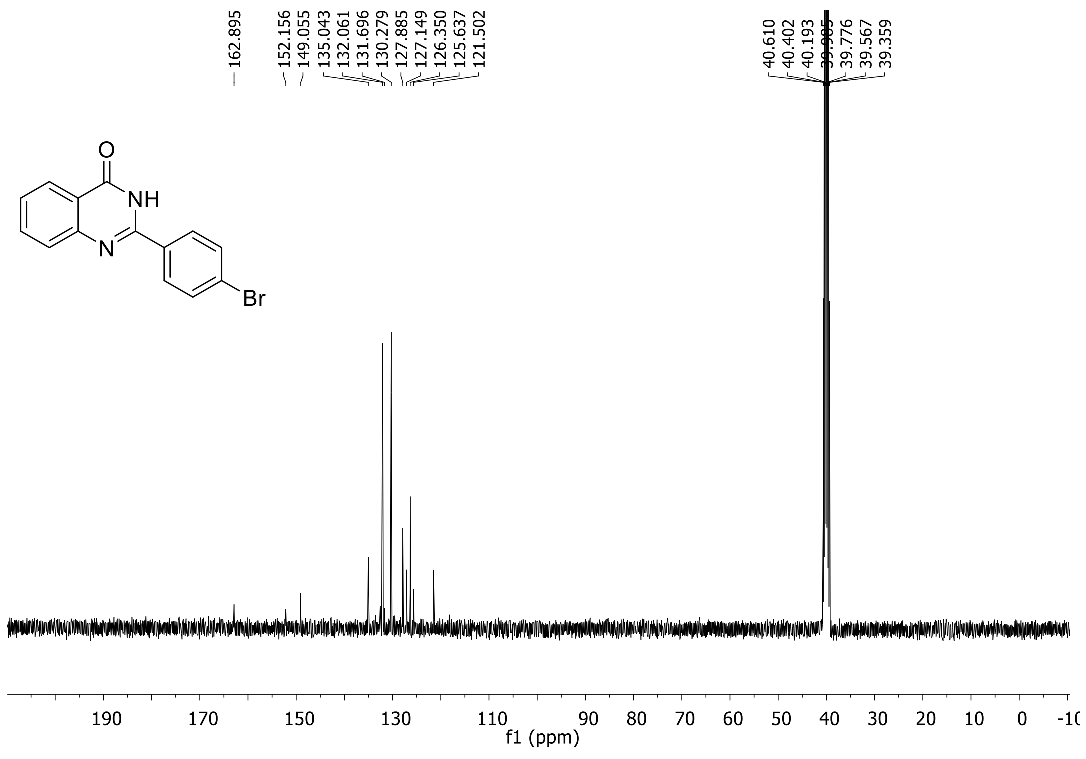

Figure S93. ^{13}C NMR of 2-(4-bromophenyl)quinazolin-4(3*H*)-one **(5m)**
